# Self-splicing introns in genes of *Bastillevirinae* bacteriophages

**DOI:** 10.1093/nar/gkaf121

**Published:** 2025-02-27

**Authors:** Martyna Węglewska, Joanna Gracz-Bernaciak, Sophia Bałdysz, Grzegorz Nowicki, Jakub Barylski

**Affiliations:** Department of Molecular Virology, Faculty of Biology, Adam Mickiewicz University, 61-614 Poznań, Poland; Department of Molecular Virology, Faculty of Biology, Adam Mickiewicz University, 61-614 Poznań, Poland; Department of Molecular Virology, Faculty of Biology, Adam Mickiewicz University, 61-614 Poznań, Poland; genXone, Inc., 62-002 Złotniki, Poland; Department of Molecular Virology, Faculty of Biology, Adam Mickiewicz University, 61-614 Poznań, Poland

## Abstract

Group I introns are self-splicing ribozymes that can be found in eukaryotes, prokaryotes, and quite often in their viruses. The distribution, structure, and splicing of group I introns in genes of some phage taxa like the *Tevenvirinae* or *Twortwirinae* was extensively studied. On the other hand, the prevalence of intervening sequences in most other clades of bacterial viruses remains mostly unexplored. In this paper, we describe group I autocatalytic introns in genes of phages from the *Bastillevirinae* subfamily. This taxon belongs to the *Herelleviridae* family and consists of 15 genera and 37 species, including viruses with strong antimicrobial potential. A bioinformatic search for intron-related RNA structures revealed the presence of 45 intervening sequences within 37 genes that belong to four gene families. Eight of the nine genes selected for experimental validation were spliced—four only in an infected bacteria but additional four self-spliced *in vitro*. Interestingly, one of the studied genes undergoes alternative splicing. To sum up, our findings expand the knowledge on the distribution and diversity of group I introns and shed new light on this neglected aspect of phage transcriptomics. Additionally, in the course of our study, we demonstrated the effectiveness of nanopore sequencing in elucidating prokaryotic splicing mechanisms.

## Graphical Abstract

**Graphical Abstract:**
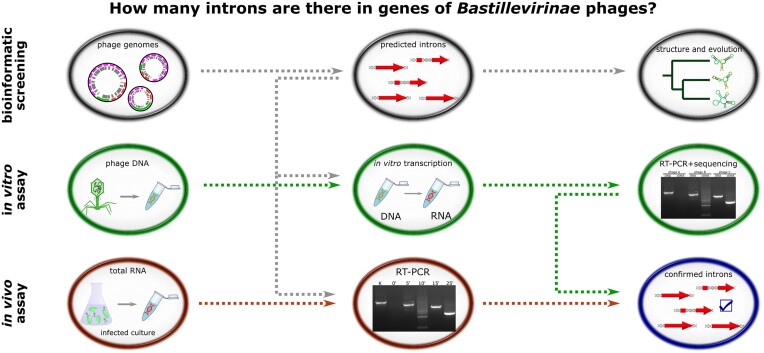

## Introduction

From the moment of their discovery, introns were considered to be a hallmark of eukaryotic organisms. This assumption was challenged in the 1980s when intron-interrupted genes were found in archeons [1], bacteria, and bacteriophages [2–5]. Unfortunately, these discoveries never really made it to molecular biology handbooks or the common imaginarium of scientists. Nevertheless, there is a growing consensus that introns, particularly those from the autocatalytic group I (gpI), may be common in some families of bacteriophages [6–8].

The gpI introns have been identified in bacterial and archaeal genomes as well as nuclei mitochondria or plastids of eukaryotes (e.g. fungi, plants, algae, and amoebae) and notably in viruses. These elements often reside in ribosomal RNA and transfer RNA genes, but can also be found within protein-coding sequences, especially in bacteriophage genomes [9]. After transcription, they form a ribozyme structure that catalyzes splicing of the parent RNA molecule using guanosine as a cofactor. Many gpI introns are mobile and can insert into an homologous intronless gene through a process known as homing. The invasion into the target site is initiated by the introduction of a double-strand break by the homing endonuclease. Then, the cleavage is repaired via homologous recombination using an intron-containing sequence as a template [10]. Homing nucleases are usually encoded within the loop—a part of the intron that is not directly involved in the ribozyme activity.

The function of introns in phage genes remains largely elusive. Up to date, several competing hypotheses have been proposed, but none have been conclusively proven. For example, there are some indications that gpI introns may act as environmental sensors by splicing only in certain conditions and halting the expression of the gene until these conditions are met [11]. This would be in line with the observation that they tend to invade specific genes involved in nucleotide or DNA metabolism, presumably to regulate their function. On the other hand, mobile introns are often considered selfish elements that do not provide any fitness benefit. In such scenario, they integrate into essential genes only to make themself harder to eliminate [11].

Up to date, over 100 intron sequences were found in phages representing nearly 30 genera that belong to 6 different families and display very different genome organizations (see Supplementary Table S1). Among them, we can find both phage SPO1—an exemplar of the *Herelleviridae* family that encompasses bastilleviruses and the prototype of the *Bastillevirinae* family—*Bacillus* phage Bastille [12]. Genomes of *Herelleviridae* phages seem to be relatively rich in gpI introns [12]. The distribution of intervening sequences among members of the *Twortvirinae* subfamily that infect *Staphylococcus* (e.g. Twort, K, G1, ISP, Sb-1, A5W, Staph1N, A3R, Fi200W, 676Z, P4W, MSA6, Romulus, and Remus) is well documented and reviewed in [6, 13], or [14]. Curiously, we could not find a comprehensive study describing introns in phages from the related *Bacillus*-infecting *Bastillevirine*. The only papers we were able to find describe individual introns in HoodyT, Cam0003, Megatron, or Bastille genomes [15]. Hence, the existing knowledge seems scattered and fragmentary, especially considering that the subfamily includes 37 species grouped into 15 distinct genera [16]. Some of these viruses infect and kill deadly pathogens like *B. anthracis*, or microorganisms involved in food spoilage and food poisoning such as *B. cereus* or *B. coagulans* [17–19]. Other *Bastillevirinae* phages can be a threat to industrial fermentations that rely on *B. subtilis* strains [18, 20]. Hence, bastilleviruses are a large, economically significant group of phages and we have strong evidence to suspect that introns can be easily found in their genomes, but we know very little about the prevalence, structure, and splicing of these sequences.

To address this knowledge gap, we analyzed the distribution and splicing of gpI introns in genomes of phages from the *Bastillevirinae* subfamily.

Our results expand the catalog of known gpI introns and expose their taxonomic and genomic distribution in the *Bastillevirinae* subfamily. We also demonstrate splicing of diverse introns that may affect the expression of core phage genes. Finally, we confirm that nanopore sequencing of complementary DNA (cDNA) can be effectively applied to study splicing of phage introns.

## Materials and methods

### Bioinformatic screening for gpI introns in genomes of *Bastillevirinae* phages

We retrieved a collection of 52 phage genomes classified as *Bastillevirinae* from the RefSeq database on 23 June 2023 (see Supplementary Table S2) and searched them against RFAM (general database of structural RNAs, version 14.9 [21]) and GISSD (specialized gpI intron database [22], last downloadable version 5.03.2014) using the software InfeRNAl v1.1.4. After the initial search, hits embedded in other higher scoring alignments were removed (the procedure similar to BLAST with “culling_limit” option set to 1) and we identified intron-related matches—hits to records RF00028 and RF00029 representing gpI and gpII autocatalytic introns from RFAM and all models from GISSD. A close inspection revealed that some of these hits represent only partial matches to conserved structural motifs of the gpI ribozyme and are separated from the rest of the intron by a long unstructured loop that often contains a homing endonuclease. Thus, we extracted 2500-bp-long regions flanking hits to intron-related models (merging any overlapping sequences) and aligned them to protein families from the PHROG database (version 4 [23]) to resolve the structure of the split genes. Briefly, the neighborhood of each discovered intron was translated in three forward reading frames and the resulting amino acids sequences were scanned for similarity to the PHROG HMMs (Hidden Markov Models) with HMMer3 3.1b2. Results were parsed to find the top-scoring split alignment between a region and protein family, estimate positions of exons and introns based on its borders, and locate intron-embedded CDSs (protein Coding Sequences). The intron search pipeline described above is available as “find_introns” script in the Zenodo repository (see “Data availability” section). Re-annotation of CDSs with InterPro domains was performed in the Geneious 2024.0.7 environment.

### Evolutionary and structural analysis of the predicted introns

To capture *Bastillevirinae* introns with any exon parts involved in splicing (e.g. sequences binding the internal guide or conserved integration sites), we extracted the entire intervening sequences with 15-nucleotide flanking regions upstream and downstream. Such an approach mirrored the methodology used for GISSD—a database that we used as a reference [22]. However, we found that the database is highly redundant and biased toward certain intron types, with subgroup C constituting 67% of the sequences. To address this, we dereplicated the dataset using CD-HIT [24] by collapsing the sequences with identity and length similarity above 99% into a single representative sequence.

Then, we constructed an evolutionary tree of intron catalytic cores. Nonredundant introns (*n* = 1399) were aligned to the RFAM gpI model (RF00028) using the Infernal cmalign tool with the “–matchonly” option to exclude variable insertion sequences (e.g. homing nuclease genes) and focus on conserved RNA structures. Poorly aligned sequences (i.e. columns with >50% gaps, and then sequences with <50 aligned bases) were pruned. For each aligned sequence, a coverage of the reference model was calculated as a ratio between number of nucleotides aligned by cmalign (matches) and consensus length of the RF00028 covariance model (CLEN = 251). The resulting alignment of 1224 introns was used to construct a maximum-likelihood tree with IQ-tree v2.3.6 [25].

To predict the secondary structures of the introns from *Bastillevirinae*-associated tree branches, we used LinearTurboFold, a tool optimized for simultaneously aligning and folding long RNAs that was previously tested on gpI introns [26]. Predicted structures were visualized using the R2DT workflow [27] in a template-free model.

Scripts and commands used to perform the above analyses are available in the Zenodo repository associated with the paper, while the alignments, phylip-formatted trees, and predicted structures are deposited in a Figshare archive.

### Biological material

We selected eight intron-invaded genes from four viruses classified into different genera (see Table 1) and two additional phages with previously confirmed spliced introns as a positive control for further experiments. The first of them was the bacteriophage Bastille—the exemplar of the subfamily, with a gpI intron identified in the DNA polymerase gene [28]. The second was the *Bacillus* phage SPO1, the archetype of the entire *Herelleviridae* family, which is not a bastillevirus per se but like Bastille has an intron in the DNA polymerase gene [29]. Intron-carrying genes selected for the laboratory tests and phages that encode them are presented in Table 1.

**Table 1.** The analyzed introns, phages, and their propagation hosts

No.	Phage	Genus	Propagation host	Gene with intron
1	phiAGATE	*Agatevirus*	*Bacillus pumilus* GL1	rNDPL, Pol, TerL
2	phiNIT1	*Nitunavirus*	*Bacillus subtilis* NATTO (NAFM5)	rNDPL, Pol
3	SIOphi	*Siophivirus*	*Bacillus subtilis* 10 (University of Wroclaw, Faculty of Biotechnology)	Pol, TerL
4	W.Ph.	*Wphvirus*	*Bacillus thuringiensis* Berliner 1915 (ATCC 10792, DSM 2046)	Pol
5	Bastille	*Bastillevirus*	*Bacillus thuringiensis* subsp. Kurstaki	Pol
6	SPO1	*Okubovirus*	*Bacillus subtilis* B3/27	Pol

rNDPL, ribonucleoside-diphosphate reductase large subunit; TerL, terminase large subunit; Pol, polymerase.

### Bacteria and phage propagation

Bacterial strains were cultured in trypticase soy broth (TSB) or on tryptone soy agar plates and grown at 30°C.

To produce the phage lysates, an overnight culture of host bacteria was diluted in fresh, warm (30°C) TSB medium to reach OD_600_ ∼0.3 and incubated at 30°C with vigorous shaking (∼200 rpm/Eppendorf ThermoMixer^®^) to reach OD_600_ ∼0.5. Then, the culture was infected with 0.5–1 ml of phage suspension (viruses isolated from a single plaque) and incubated at 30°C with vigorous shaking to propagate the virus until the culture was clear (complete lysis of host cells).

Bacteriophage virions were purified by CsCl gradient centrifugation as described in [30]. Briefly, particles present in the lysate were precipitated by the addition of 100 g of PEG 8000 and 58.4 g NaCl per liter of lysate. After overnight incubation at 4°C (∼18 h), samples were centrifuged (15 000 × *g*/15 min/4°C/Sigma 3-K30 centrifuge) and the pellet was resuspended in a lambda diluent [20 mM Tris–HCl, 10 mM MgSO_4_, 0.1 M NaCl (pH 7.5)]. The resulting suspension was purified by chloroform extraction and mixed with CsCl (0.75 g per ml of the original lysate). The mixture was centrifuged (155 000 × *g*/21 h/4°C/Beckman Optima™ L-90 K Ultracentrifuge). Finally, the phage-containing band was carefully collected and dialyzed in Slide-A-Lyzer G2 10K MWCO Cassettes (Thermo Scientific™) for 18 h at 4°C against an SM buffer with increased salinity [1 M NaCl, 8 mM MgSO_4_, 25 mM Tris–HCl (pH 7.5)] and then for 3 h in room temperature against a standard SM buffer (0.1 M NaCl). Finally, the concentrated phage solution was filtered through syringe filter (Millex™, Millipore) with 0.45 μm pore size and stored at 4°C for further experiments.

### Purification of bacteriophage DNA

DNA from purified phage particles was isolated by phenol–chloroform extraction. Briefly, virions were precipitated by mixing the phage lysate with 20% PEG in 2.5 M NaCl (1:1) and incubating the mixture for 30 min at 4°C. Each sample was centrifuged (18 830 × *g/*15 min/4°C/Hettich Universal 32R) and the pellet was resuspended in 100 μl of the lambda diluent. Phage suspension was treated with equal volume of the phenol:chloroform solution, vigorously vortexed to emulsify (twice) and centrifuged (18 830 × *g/*1 min/20°C/Hettich Universal 32R). The aqueous phase was then transferred to a sterile tube, where the DNA was precipitated by the addition of an ice-cold mixture of 96% (v/v) ethanol and 3M sodium acetate (25:1). After incubation (30 min at −20°C), the sample was centrifuged (21 910 × *g/*40 min/ 4°C/Hettich Universal 32R) and the precipitate was washed with 200 μl of ice-cold ethanol [70% (v/v)]. After another round of centrifugation (21 910 × *g/*5 min/4°C/Hettich Universal 32R), the DNA pellet was dried out by 5–10 min incubation in 37°C, and resuspended in nuclease-free water.

### PCR amplification of putative intron sequences

To amplify the selected introns together with flanking exon fragments, we designed primer pairs that included a forward primer with an RNA polymerase T7 promoter necessary for *in vitro* transcription (listed in Supplementary Table S3). To amplify the sequence of interest, phage DNA was diluted to 5 ng/μl using nuclease-free water. Then, 10 ng of DNA was mixed with a Master Mix solution containing polymerase (NZYTaq II 2× Colourless Master Mix, NZTtech), 0.5 μM of forward and reverse primers and the reaction was adjusted to 50 μl with nuclease-free water. The amplification was conducted under the following cycling conditions: 5 min, 95°C (initial denaturation), 35 cycles of 30 s, 95°C (denaturation), 30 s, 55°C (primers annealing), 30 s, 72°C (primers extension), and 5 min, 72°C (final extension) in T100 Thermal Cycler (Bio-Rad). The PCR products were assessed by electrophoresis in a 1% agarose gel.

### *In vitro* splicing assay

To confirm the ability of the predicted introns to self-splice, we synthesized transcripts containing the intron and fragments of both flanking exons by *in vitro* transcription.

The PCR-amplified intron-carrying fragments with the T7 promoter at the 5′ end (see above) were used as templates for the transcription procedure. The reaction was performed using the MEGAscript™ T7 Transcription Kit (Invitrogen) according to the manufacturer’s instructions. The mixture was incubated at 37°C for 3 h to synthesize RNA and allow the transcript to mature. Samples were then treated with 1 μl Turbo DNase™ (15 min, 37°C) to remove the template DNA. RNA was stored at −20°C for further analysis. In order to check the size of the obtained RNA products, the transcribed samples were resolved on an agarose gel.

The RNA generated by the *in vitro* transcription (∼500 ng) was reverse-transcribed into cDNA with a gene-specific reverse primer (listed in Supplementary Table S3) and the QuantiTect Reverse Transcription Kit (QIAGEN) according to the manufacturer’s protocol. Potentially spliced sequences were re-amplified (as described above). Then, the PCR products were resolved on a agarose gel and sequenced to determine the exact positions of the splice sites. cDNA libraries were prepared using a Ligation Sequencing Kit (SQK-LSK109) according to the manufacturer’s protocol. The sequencing was performed on the GridION X5 device with R9.4.1 flow cell (Oxford Nanopore Technologies, Oxford, UK) under the control of MinKnow v3.6.5, and basecalling was executed by Guppy v3.2.10 with a “high accuracy” model. The sequencing procedure was commissioned to genXone, Złotniki, Poland.

To find the boundaries of introns and exons, the obtained reads were mapped to genomic sequences with the “confirm_introns” script from the Zenodo repository (see “Data availability” section). Briefly, the script ran a modified version of Porechop (adjusted to facilitate the import of custom adapters and barcodes) to trim and demultiplex data based on the primer sequences present at both ends of the reads. Then each library was filtered using Filtlong to remove reads with a mean expected error rate above 10% and/or length below 500. Clean reads were mapped to the genomic sequences with Minimap2 v2.28 [31] (spliced alignment mode with no preferred splice flanks) and the resulting alignments were filtered in samtools to keep only primary alignments with overall mapping quality above 30. Finally, the script ran spliced_bam2gff from the Pinfish suite v0.1.0 (ONT) to map the potential splice sites and extracted introns supported by at least 10 mapped reads that correspond to >10% of the total mapping sequences. For more readable visualization of the intron support, we randomly subsampled ∼50 reads from each library using seqkit, mapped these reads to the genome using the Geneious RNA mapper (maximum gap size 2000, other settings as in “high sensitivity” preset), and visualized the resulting alignment in the Geneious environment [32].

### *In vivo* splicing dynamics

To observe splicing in the course of phage replication, we collected RNA samples from different stages of infection and examined them with a reverse transcriptase–polymerase chain reaction (RT–PCR) assay similar to that used to detect *in vitro* splicing.

Overnight cultures of the host bacterium were diluted in fresh, pre-warmed TSB medium to reach OD_600_ < 0.1. Flasks with bacteria were incubated at 30°C with vigorous shaking (∼200 rpm, Corning^®^ LSE™ Shaking Incubator) until OD_600_ = 0.35–0.4 was reached (corresponding to ∼1×10^7^–1×10^8^ CFU/ml depending on the strain). Then, the concentrated phage (purified with CsCl, dialyzed, and filtered) was added to the host culture to reach the final multiplicity of infection ∼10. Samples were collected before and after infection at 5-min (phiAGATE, Bastille, SIOphi) or 15-min (phiNIT1) intervals. Time intervals were determined based on the duration of the replication cycle of each phage. At each timepoint, 9 ml of culture was mixed with 1 ml of stop-solution (10% phenol in ethanol, −20°C). The mixture was then centrifuged (9500 rpm, 5 min, 4°C, Sigma 3–16PK centrifuge) and the obtained pellet was resuspend in 100 μl of lysis buffer (FastRNA Lysis Buffer, FastRNA™ SPIN Kit for Microbes). To avoid RNA degradation, the sample was immediately frozen in liquid nitrogen and stored in −80°C until RNA isolation.

RNA was isolated using the FastRNA™ SPIN Kit for Microbes (MP Biomedicals) with a modified protocol. Briefly, samples were placed on ice and 700 μl of FastRNA Lysis Buffer was added to the resuspended pellet and mixed by pipetting. Next, lysates were transferred on a zirconium silicate Lysing Matrix and homogenized using the FastPrep-24™ Instrument (6 m/s, 1 min, MP Biomedicals). The samples were loaded into SPIN columns and purified. RNA was eluted with 20 μl of DNase/RNase-free water with RiboLock RNase Inhibitor (Thermo Scientific™).

RNA isolated from each sample was reverse-transcribed to cDNA that was used as a template for the PCR reaction (protocol described above). PCR products from all timepoints were run on a 1% agarose gel to compare their sizes and estimate the time of intron splicing.

## Results

### Bioinformatic screening for gpI introns in genomes of *Bastillevirinae* phages

**Figure 1.**
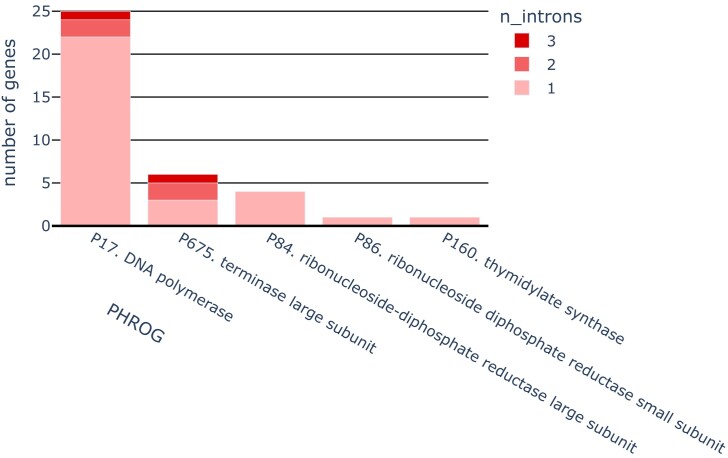
Incidence of the detected introns in different gene families.

In the analyzed phage genomes, we detected 72 local alignments to gpI intron models. Structural analysis of 37 neighboring genes grouped these alignments into 45 distinct introns, with a mean length of 802 ± 342 bases. Most of the intervening sequences reside in DNA polymerase genes. Nearly half of the genes from this family are split, some by two or even three introns. Presence of the intervening sequences in terminase large subunit genes is not that frequent (5/52), but some of them are also split by two or three introns. Few remaining introns were found in thymidylate synthase and both large and small subunits of ribonucleoside-diphosphate reductase (rNDPL). For more details about distribution of introns in genes and genomes, see Fig. 1 and Supplementary Table S4.

Most of the discovered introns displayed some similarity to the IA subgroup of gpI (models for IA2 and IA3 were responsible for 55/72 hits). In most (32/45) introns, the ribozyme sequence was disrupted by a loop encoding a homing nuclease. In some of these cases, the 5′ part of the intron core was aligned to a different subgroup than the 3′ part. In 10 cases, the ribozyme signature could not be found on one side of the loop at all. The predicted intron structures are presented in Supplementary Table S5.

The majority of the loop-encoded proteins were classified into one of three PHROG clusters of “HNH endonucleases” (see Table 2). The remaining CDSs encoded “endonucleases”, “homing endonucleases”, or “homing endonucleases with LAGLIDADG motif”. Most of these protein families carry domains typical for known homing endonucleases (HNH, GIY-YIG, or LAGLIDADG homing endonuclease domains and DNA-binding domain of intron-encoded endonucleases superfamily or NUMOD4 DNA-binding motif). However, in one nuclease group, we found a less typical signature of VSR (Very Short patch Repair endonuclease) superfamily. Finally, we could not detect any conserved domain in a group of proteins that the PHROG database defines as “endonucleases”.

**Table 2.** Intron-encoded proteins

PHROG	Name	CDSs	InterPro_domains	Genes	Genera
P59	HNH endonuclease	12	**HNH_nuc** [IPR003615] (12), **His-Me finger endonucleases** [IPR044925] (12), **unnamed** [G3DSA:3.90.75.20] (12), **NUMOD4** [IPR010902] (11), **WH-like_DNA-bd_sf** [IPR036388] (10), **DNA-binding domain of intron-encoded endonucleases** [SSF64496] (10), **I-HmuI_NUMOD-like** [PF22083] (8), **Intron_nuc_1_rpt** [IPR003647] (4)	**phrog_17**. DNA polymerase (10), **phrog_84**. rNDPL large subunit (1), **phrog_86**. rNDPL small subunit (1)	*Wphvirus* (5), *Bastillevirus* (3), *Nitunavirus* (1), *Agatevirus* (1), *Matervirus* (1), *Eldridgevirus* (1)
P314	homing endonuclease	2	**GIY-YIG_endonuc** [IPR000305] (2), **DNA-binding domain of intron-encoded endonucleases** [SSF64496] (4), **NUMOD3** [IPR003611] (2), **Intron_endoG1** [IPR006350] (2), **GIY-YIG_endonuc_sf** [IPR035901] (2), **GIY-YIG_HE_I-TevI_like** [cd10437] (2), **I-TevI_DNA-bd [**IPR048681] (1)	**phrog_84**. rNDPL large subunit (1), **phrog_160**. thymidylate synthase (1)	*Nitunavirus* (1), A*gatevirus* (1)
P559	HNH endonuclease	4	**VSR endonuclease** [G3DSA:3.40.960.10] (4)	**phrog_675**. terminase large subunit (4)	*Moonbeamvirus* (2), *Nitunavirus* (1), *Caeruleovirus* (1)
P2081	HNH endonuclease	9*	**DNA-binding domain of intron-encoded endonucleases** [SSF64496] (9), **WH-like_DNA-bd_sf** [IPR036388] (8), **HmuI_NUMOD-like** [PF22083] (5), **Intron_nuc_1_rpt** [IPR003647] (2)	**phrog_17**. DNA polymerase (9)	*Wphvirus* (3), *Nitunavirus*(2), *Agatevirus* (2*), *Moonbeamvirus* (2*)
P2340	endonuclease	7	**no conserved domain detected**	**phrog_17**. DNA polymerase (7)	*Caeruleovirus* (3), *Nitunavirus* (1), *Agatevirus* (1), *Moonbeamvirus* (1), *Wphvirus* (1)
P11023	homing endonuclease with LAGLIDADG motif	1	**LAGLIDADG_2** [IPR004860] (1), **Homing_endonucl** [IPR027434] (1)	**phrog_17**. DNA polymerase (1)	*Nitunavirus* (1)

Numbers in the first columns are identifiers from the PHROG database. The asterisk “*” denotes counts that include multiple CDSs representing partial proteins.

Most introns contained either one internal CDS or none. Nonetheless, three intervening sequences contained multiple distinct PHROG alignments. In the case of the phage phiNIT1 DNA polymerase intron, this seems to be a result of sequential colonization of a single gene by two distinct introns containing different types of homing nucleases. The situation looks different for introns within DNA polymerases of phages phiAGATE and Moonbeam. In each of these introns, we found two partial alignments to the P2081 HNH endonuclease family that are likely fragments of the same gene split by an internal frameshift.

**Figure 2.**
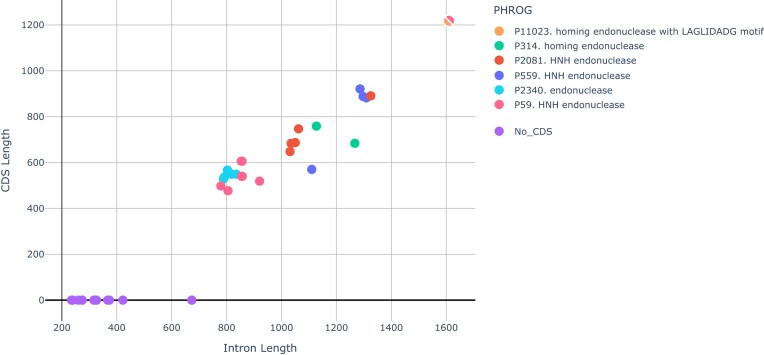
Lengths of introns encoding different types of homing nucleases. Split pointer represents an intron carrying two internal CDSs that belong to different families (P59 and P11023).

The types of the internal protein seem to be closely related to the length of the intron. As expected, the intron size is proportional to the sum of lengths of the embedded CDSs (Pearson correlation coefficient 0.96, see Fig. 2) and non-gene-coding sequences span only 350 ± 108 bases.

All intron-carrying genes were found in phages from 9 of the 15 analyzed genera (see Table 3). Among Wphviruses infecting *B. cereus*, we identified as many as 11 genomes with split genes, but this may simply be the result of a large number of known strains. Within the intron-invaded genera the prevalence of intervening sequences was also uneven, with half or more genomes harboring at least one.

**Table 3.** The taxonomic distribution of discovered introns

Genus	Invaded genomes	Invaded genes	Introns
*Wphvirus*	11/16	11	11
*Caeruleovirus*	3/6	4	4
*Bastillevirus*	3/4	3	3
*Agatevirus*	2/4	6	7
*Nitunavirus*	3/3	7	8
*Matervirus*	1/1	1	1
*Moonbeamvirus*	1/1	2	4
*Eldridgevirus*	1/1	1	1
*Siophivirus*	1/1	2	6

### Evolutionary and structural analysis of the predicted introns

To identify conserved parts of the gpI ribozyme structure, we aligned the predicted introns to the RFAM gpI covariance model. Almost all introns from bastilleviruses and representatives of subgroups IA, IB, and IC aligned well, with a median coverage of the reference model exceeding 60%. On the other hand, less studied subgroups like ID or IE remained largely unaligned (median coverage of 37.8% and 3.6%, respectively). This is unsurprising, as no introns from these subgroups were included in the RFAM seed alignment. Thus, pruning poorly aligned sequences removed 50% examples of ID and nearly 84% of IE. For detailed analysis of the alignment see Supplementary Tables S7–S12.

**Figure 3.**
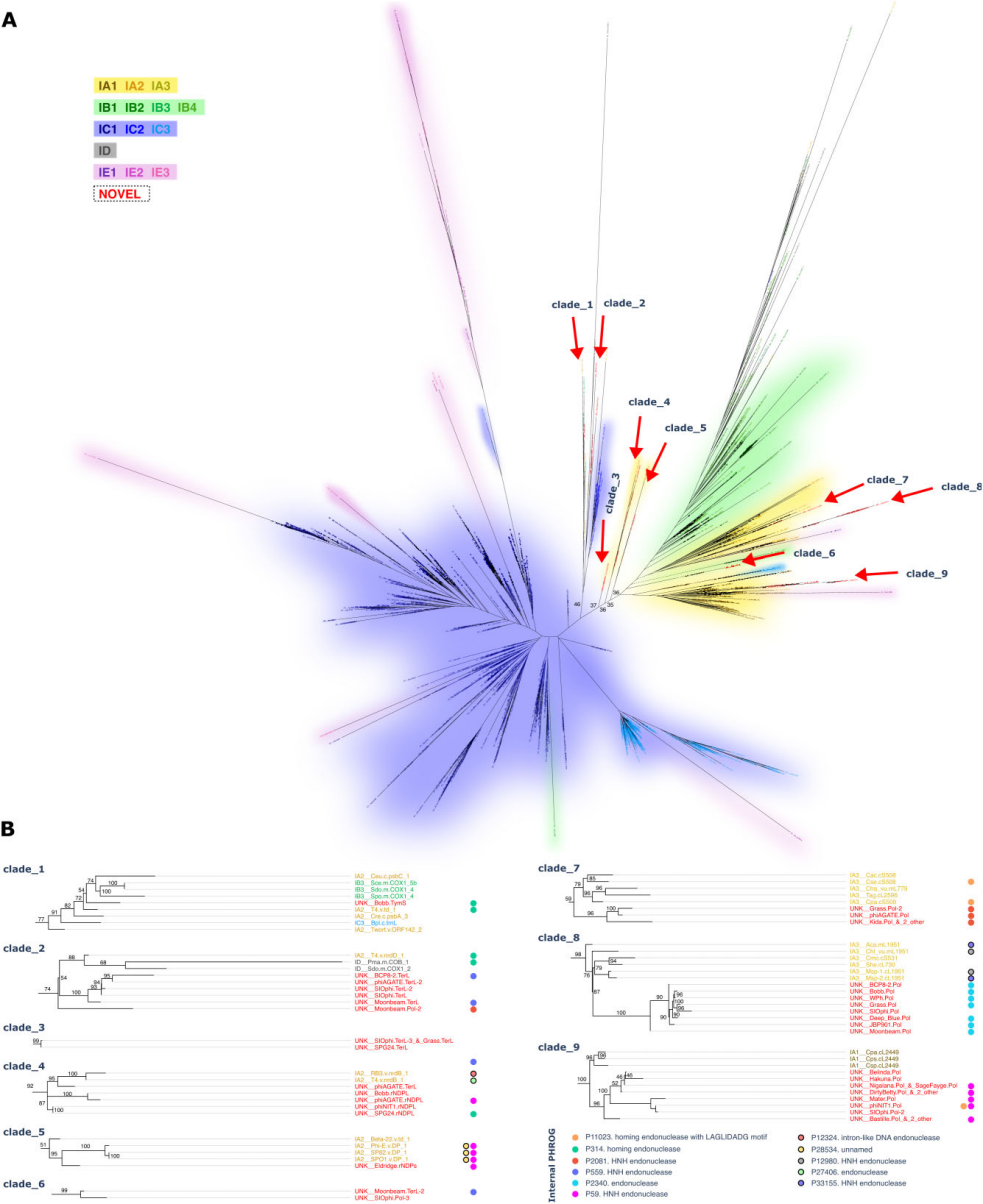
Consensus phylogenetic tree of intron catalytic cores, constructed from the alignments to RFAM gpI covariance model. (**A**) The complete tree, colored by the intron subgroup as indicated in the legend on the left. Arrows highlight clades containing *Bastillevirinae* introns. (**B**) Magnified view of these clades. Leaf labels are colored according to the subgroup scheme presented in panel (A), circular markers to the right indicate the presence of specific families of protein-encoding genes within the introns (if applicable). Numbers labeling branches in both panels represent bootstrap values.

The maximum-likelihood tree based on the alignment (Fig. 3 and Supplementary Fig. S1) generally reconstructed known intron relationships with a notable exception that the mentioned ID and IE subgroups were partially displaced within other clusters.

The tree revealed that *Bastillevrinae* introns segregate into nine distinct clades. With the exception of clades 5 and 7, all these clades find strong support in the bootstrap analysis (>70%). Five of them include introns from other bacteriophage taxa, like T4-related phages (clades 1, 2, and 4) or *Twortwirinae* (clade 1), and *Okubovirinae* (clade 5) that are sister subfamilies of bastilleviruses. Many of those introns were previously classified to subgroup IA2 that is traditionally associated with bacteriophage introns [33–36]. Surprisingly, this subgroup appears heterogeneous—it is composed of several distinct branches with barely detectable sequence similarity and association poorly supported by bootstrap values (<46%). This suggests that the diversity of phage introns may extend beyond the currently available subgroups. Indeed, some phage subgroups, like clade 1, seem equally distant to examples of subgroup IA2 from *Chlamydomonas* chloroplasts, intervening sequences from *Bryopsis* chloroplasts included in IA3 and *Saccharomyces* mitochondrial introns annotated as IB3. Finally, clades 7 and 8 emerged as internal branches of the IA3 subtree, and clade 9 nested within the IA1 cluster. According to our best knowledge, these groups were not previously observed in phage genomes.

Analysis of the intron sequences indicates a decoupling between the intron subgroup and the associated nuclease gene. While some branches of the tree predominantly contained specific endonuclease types, others included nuclease-free sequences or a mixture of different nuclease types. Notably, some nonphage gpI sequences (e.g. IA3 introns from algae and amoebae) encoded homologues of phage homing nucleases detectable by HMM models in the PHROG database.

Predicting the secondary structures of the *Bastillevirinae* introns and their relatives was challenging due to their considerable and variable size (173–3279 bases), overlap between conserved gpI elements and nuclease genes, complex and segmented catalytic cores as well as the unclear role of peripheral elements on the final structure. Nevertheless, most predicted structures exhibited some basic characteristic gpI intron motifs as well as a few clade-specific features (Supplementary Fig. S2). Notably, the interaction between the exon and the internal guide sequence was usually successfully identified. On the other hand, the conserved P7/P7′ helix was rarely predicted.

### *In vitro* splicing assay

**Figure 4.**
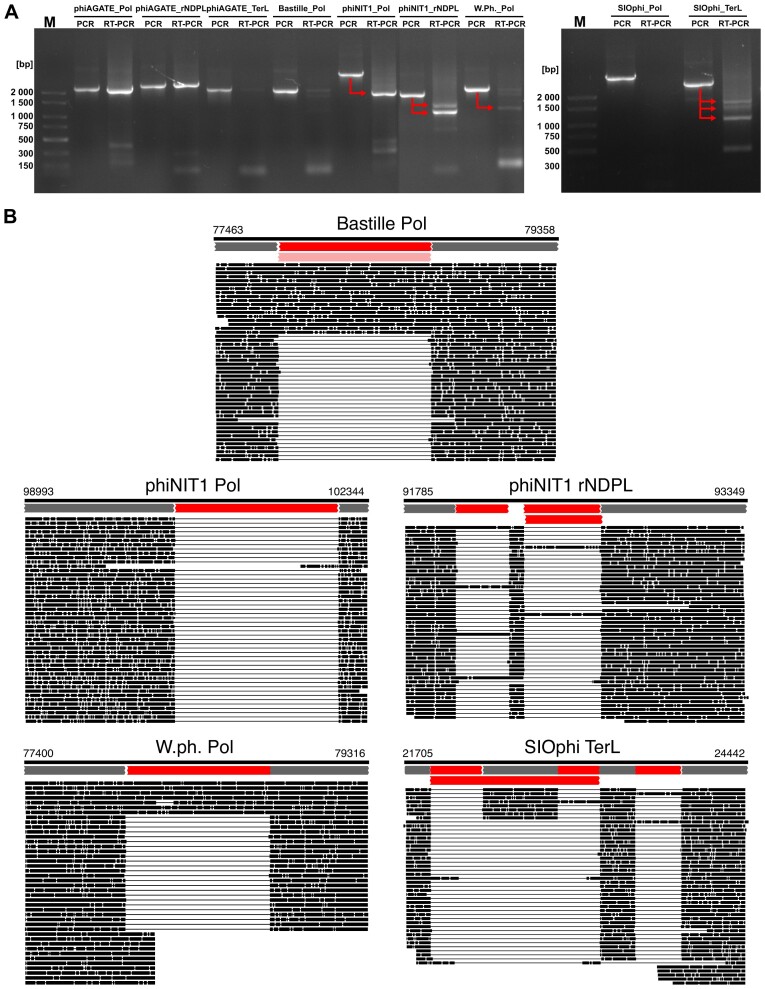
Results of the phage *in vitro* splicing assay. Panel (**A**) shows the analysis of the PCR products by an agarose gel electrophoresis. Each pair of lanes includes a PCR product obtained from the phage genomic DNA (PCR) and cDNA (RT–PCR). Arrows lead from full-length gene sequences to their presumed splicing products. The phage and gene name are indicated above the line. The lengths of the DNA ladder bands (M) are shown on the left. Panel (**B**) contains results of the nanopore sequencing of cDNA amplicons mapped to their original genomic sequences. Gray bars, at the top of each picture, represent exon positions predicted by alignment of PHROG HMMs to the genome. Red bars show positions of the introns inferred based on splits observed in the mapped reads. Complete mapping results and intron annotations are available as bam and gff files in the Figshare repository (see “Data availability” section).

After the bioinformatic screening, we selected eight genes with predicted introns and two genes containing known introns and tested their ability to self-splice. To assess this, we amplified fragments of these genes that enclosed putative introns, transcribed them *in vitro*, then left them to excise themselves from the parent RNA. At the end of the experiment, the total RNA in the mixture was reverse-transcribed to cDNA, which was in turn used as a template for the PCR reaction. A comparison of the full-length genomic and cDNA amplicons after the *in vitro* splicing assay is presented in Fig. 4.

After the *in vitro* assay, we observed truncated cDNA for the phiNIT1 and W.Ph. polymerases as well as the large subunits of phiNIT1 rNDPL and the SIOphi terminase (TerL). These results allow us to assume that introns within the abovementioned sequences are autocatalytic ribozymes that excise themselves from the RNA molecules without any additional factors.

In general, nanopore sequencing confirmed that the observed shortening of the PCR products was a result of intron splicing (Fig. 4B.). Additionally, it confirmed splicing of an intron in the Bastille DNA polymerase. Thus, cDNA analysis revealed the presence of 8 introns in 5 out of 9 of the studied genes (namely Bastille, phiNIT1, W.Ph. polymerases as well as phiNIT1 rNDPL, and SIOphi TerL). Interestingly, a detailed analysis of the mapped reads revealed an additional small exon between two introns in a large subunit of phiNIT1 rNDPL. Even more puzzling was the discovery that in the dominant splicing variant of the phage SIOphi terminase, one of the internal exons is skipped. This results in the joining of the 5′ splice site of the first intron directly with the 3′ site of the second.

It is worth stressing that the applied splicing assay successfully recognized two known introns which we chose as positive controls—one in the DNA polymerase of the phage Bastille (described above) and the other in a homologous gene of the related phage SPO1 (see Supplementary Table S6). This suggests that the adopted experimental approach can indeed reveal self-splicing of phage introns.

### *In vivo* splicing dynamics

*In vitro* tests confirmed that at least some of the *in silico-*predicted introns self-splice, but they might fail to detect sequences that require additional factors or specific intracellular conditions to excise themselves. Additionally, the *in vitro* assay does not provide evidence that the same introns are spliced during viral replication.

To gather such evidence, we studied changes in the length of intron-carrying transcripts in the course of a phage infection. For the *in vivo* assay, we chose sequences that self-spliced *in vitro* (polymerases of Bastille and phiNIT1 as well as rNDPL from the latter and TerL from SIOphi) and some that yielded negative results (phiAGATE polymerase, terminase, and rNDPL genes and SIOphi polymerase).

**Figure 5.**
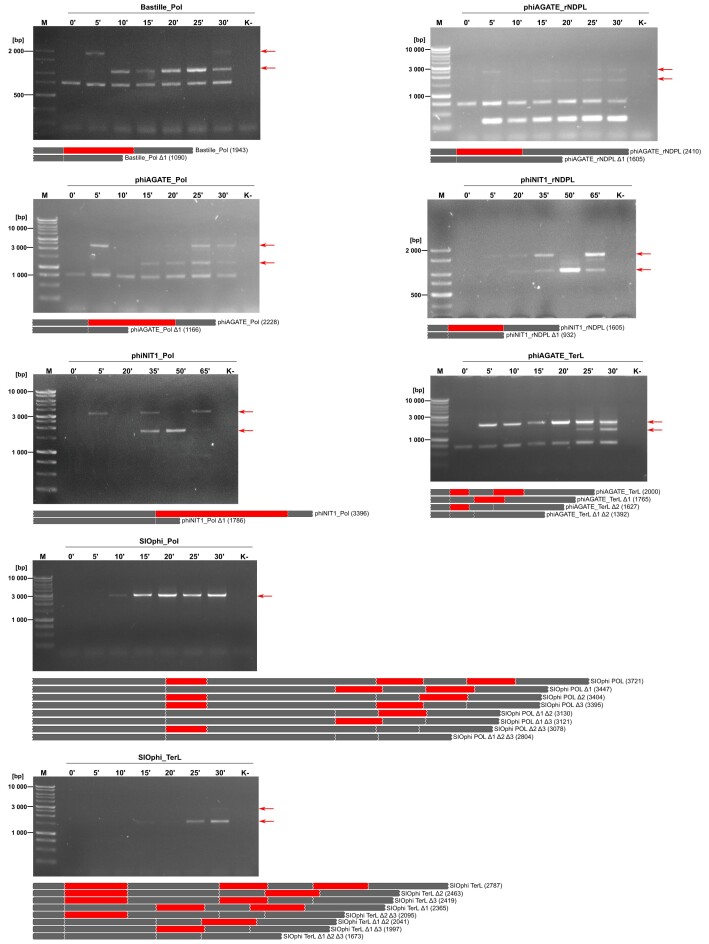
Results of the phage *in vivo* splicing assay. Each panel represents an analysis of different transcript during the phage replication cycle. Arrows indicate PCR products which correspond to the full-length gene sequences or presumed splicing product(s). Graphical representations of the analyzed sequences are below each photo. The gray bars represent exons, red—introns. The length of the sequences with and without intron(s) are in brackets. K- is a negative control, where water was used as a template for reverse transcription. The lengths of the DNA ladder bands (**M**) are shown on the left.

Interestingly, in the course of the viral replication cycle, we observed splicing for all tested fragments, even those that were not spliced *in vitro* (Fig. 5). This suggests that while some introns are capable of self-splicing in laboratory conditions, others may require factors that are present in the cellular environment to excise effectively.

For all studied genes, transcription was observed as early as 5–10 min post infection. In the case of the SIOphi polymerase and terminase genes, we observed only spliced versions of transcripts. In other cases mature transcripts were assembled later in the cycle. The first splicing products of the Bastille Pol gene were observable as early as 10 min after phage addition to the culture. In the middle of the cycle, introns are excised from the phiNIT1 polymerase DNA sequence but also from the phiAGATE DNA polymerase and rNDPL. In the second half of the cycle, splicing occurs in the case of the TerL gene of phiAGATE.

In some amplicons, we observed unexpected short DNA fragments present in all of the timepoints (both before and after infection) that may be nonspecific products of amplification (PCR artefacts).

## Discussion

Our knowledge of the distribution of gpI introns in bacteriophage genes is fragmentary at best. To mitigate this problem, we conducted an in-depth analysis of introns that colonize genomes of *Bastillevirinae* phages. In a single phage subfamily, we discovered 45 distinct intervening sequences within 37 genes that belong to four orthologous clusters.

Four of intron-carrying genes that were tested splice without the support of any additional factors. For the remaining four, we observed excision in phage-infected cultures. It is unclear whether those sequences require some specific cellular factor(s) to function properly or the physico-chemical conditions of the reaction were simply not suitable to support the ribozyme activity.

One of our most important findings is that one of studied genes seems to produce a heterogeneous population of splicing products. More than 47.2% of the transcripts from the polymerase gene of phage SIOphi skipped the entire exon. While alternative splicing is not a phenomenon commonly associated with bacteriophages, an example of a similar exon-skipping mechanism was observed in “orf142” of the bacteriophage Twort (a gene that later turned out to encode a tail tube protein [34]).

A central question that intrigued us throughout our investigation was why did introns colonize only the core genes involved in nucleotide and DNA metabolism, such as rNDPLs, DNA polymerases, and terminases. Most split genes in phages also fall within this category (see Supplementary Table S1). For example, a nearly identical catalog of intron-invaded sequences was observed for *Twortvirinae*—the sister subfamily of *Bastillevirinae* [6]. One possible explanation of such a skewed distribution is that introns may be “molecular-on-switches” that allow a swift activation of enzymes that carry out consecutive stages of bacteriophage replication. First, the rNDPL starts to convert ribonucleotides to deoxyribonucleotides. This reduces energy flow to transcription and translation, but simultaneously makes nucleotides available for another enzyme—the DNA polymerase that makes new copies of the viral genome. Finally, products of the polymerase are packaged into virions by the terminase, which is also an ATPase. This “molecular-on-switches” hypothesis would be consistent with the fact that gpI introns require GTP to splice. Hence, they are sensitive to the concentration of this molecule that may serve as a proxy of cellular energy level. A somewhat similar idea was previously proposed based on the dynamics of T4 rNDPL intron splicing [11, 37]. Excision of this intron is inhibited by the stationary phase of the host as well as chloramphenicol-induced arrest of translation and disruption of the upstream exon by the stop codon. Thus, the splicing process seems to be dependent on the ongoing translation. Authors of both papers [11, 37] argue that this is the adaptation that limits dNTP production in the stationary phase, redirecting energy to cell survival until more favorable conditions are encountered.

An alternative explanation of the curious tendency of introns to inhabit core genes may be connected with their indispensability for the virus. It was hypothesized that intron-embedded nucleases might cleave and inactivate homologous genes of closely related but intronless competitor phages to disrupt their replication. Such a phenomenon was observed for *Pseudomonas* phages ΦPA3 and ΦKZ. Both these viruses are related, but only ΦPA3 has an intron in the gene for RNA polymerase. This intron encodes an HNH nuclease that nicks and inactivates the polymerase gene of ΦKZ providing competitive advantage during a co-infection. This would explain why some phage taxa are heavily colonized by intervening sequences, while others are apparently intron-free. In such a scenario, a biased intron distribution may stem from the pressure exerted by the original recipient of the homing nuclease on related phages. They are left with an evolutionary dilemma: accept the intron, mutate the cleavage site, or lose the race for the host. This raises a question—what is the balance between the competitive advantage provided by the intron and cost of its retention? Another enigma is when, how, and why primary intron colonization occurred? At the moment, resolving these issues seem beyond our reach. The only clear thing is that colonization does not seem to be connected with the host specificity (at least within the studied phage set). Both intronless and intron-rich genera that infect the same bacterial species can be easily found.

It may be worth noting that the hypotheses that introns act as “molecular switches” or “anti-competitor weapons” are not mutually exclusive. One focuses on the regulatory role of the intron ribozyme, while the other emphasizes the importance of the loop-embedded nuclease. Considering that both gpI introns and homing endonucleases often occur independently of each other, it is likely that both elements provide some kind of advantage and they are just more effective together.

The main limitation of our study is that we could only identify sequences similar to known introns (represented by RFAM and GISSD models). Thus, we often observed alignments to different intron subgroups on each side of the nuclease loop or could not find any similarity on one side at all. This raises the question of how *Bastillevirinae* introns fit into the current taxonomy of gpI introns. Our evolutionary analysis placed them into nine distinct clades, most of which are linked to one of the three IA subgroups. However, a close inspection indicates that distances between the clades are comparable to distances between currently accepted subgroups, suggesting that available gpI subclassification may be insufficient to accommodate the true diversity of bacteriophage introns. The original division into four major (IA, AB, IC, and ID) and 10 minor subgroups was developed in 1990 [38] and survived to this day with only a few minor adjustments (such as the addition of group IE [39]). The initial groupings were based on comparison of covariance patterns of 87 gpI ribozymes known at the time, and rest on the assumption that the secondary and tertiary structure of each subgroup is highly conserved. Since that time, the number of well-annotated gpI introns has grown >20 times [22] and these elements were found in different genes, new domains of life, and various viral lineages [6, 33, 40]. It would not be surprising to encounter introns that defy the classification scheme that was developed three decades ago. One might expect that the subgroups that were based almost exclusively on analysis of eukaryotic sequences may not be suitable for bacterial, archeal, and phage specimens. Indeed, results of our analyses strongly suggest that the current taxonomy of gpI introns requires a thorough revision. We expect this revision would result in a comprehensive, fine-grained classification system that accounts for local and global similarities of the ribozyme structure, evolutionary trajectories of intron groups, and the mosaic patterns of intron colonization by HNH nucleases. Without such an update, we risk forcing newly discovered introns into an outdated framework that no longer reflects their true diversity.

Another issue is the underrepresentation of certain intron groups in databases and consequently in the covariance models used to recognize those RNA structures. The current RFAM model of gpI is clearly incomplete as its seed alignment does not include any sequence from ID or IE subgroups. Furthermore, even databases dedicated to gpI introns like GISSD are biased toward eukaryotic sequences, and models trained on their alignments may fail to recognize phage variants of autocatalytic introns. Additionally, they are skewed toward certain intron subgroups, like IC that account for ∼65% of the records in the current version of the database. Thus, constructing the valid classification and representative models of different subgroups may require comprehensive, iterative screening for introns that still hide in bacterial, archeal, and phage genomes as well as the thorough review of evidence of their splicing in literature, databases, and experimental data.

To sum up, we identified numerous introns and experimentally confirmed that some of them are indeed spliced. We also showed that the excision of these introns may give rise to alternative variants of the spliced transcript. Finally, we performed phylogenetic and structural analyses highlighted the diversity of gpI introns and placed of bacteriophage introns within a broader context. Together, our findings expand the knowledge on distribution and evolution of gpI introns, shed new light on the neglected aspect of phage transcriptomics, and demonstrate how nanopore sequencing can be successfully applied to study prokaryotic splicing.

## Supplementary Material

gkaf121_Supplemental_Files

## Data Availability

The databases (10.6084/m9.figshare.26351830), read mapping results (10.6084/m9.figshare.26348449), annotation gff files (10.6084/m9.figshare.26351959), tree details (10.6084/m9.figshare.28227962), and raw LinearTurboFold structures (10.6084/m9.figshare.28228148) are available in dedicated Figshare repositories. Demultiplexed and trimmed sequencing libraries are available at NCB as SRA records associated with BioProject PRJNA1138733. Scripts used to generate presented results are archived at https://github.com/zwmuam/Bastilevirinae_introns. Other data analyzed during this study are available from the corresponding author upon request. Final code repository is available at Zenodo (doi: https://doi.org/10.5281/zenodo.14780388).
