# Supplementary material for: Self-splicing introns in genes of Bastillevirinae bacteriophages
Source: Nucleic Acids Res. 2025 Feb 27;53(5):gkaf121. doi: 10.1093/nar/gkaf121 (PMC11878797; doi:10.1093/nar/gkaf121)

Supplementary Figure S2.
Structures of phage-related introns predicted using LinearTurboFold.

IA2__Ceu.c.psbC_1

clade_1

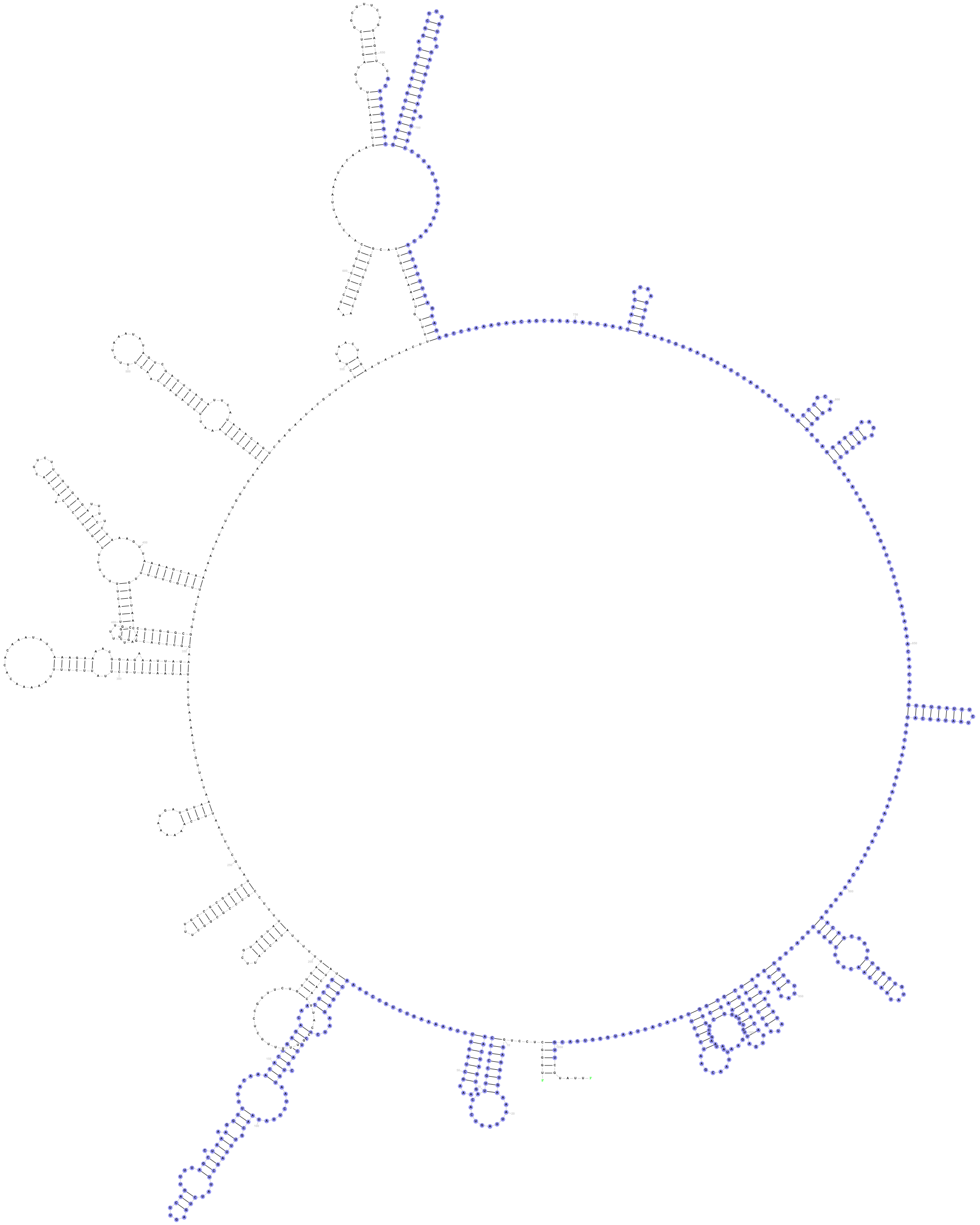

RFAM/GISSD group I CM match
embedded CDS (PHORG match)
overlap (CM and CDS)

IB3__Sce.m.COX1_5b

clade_1

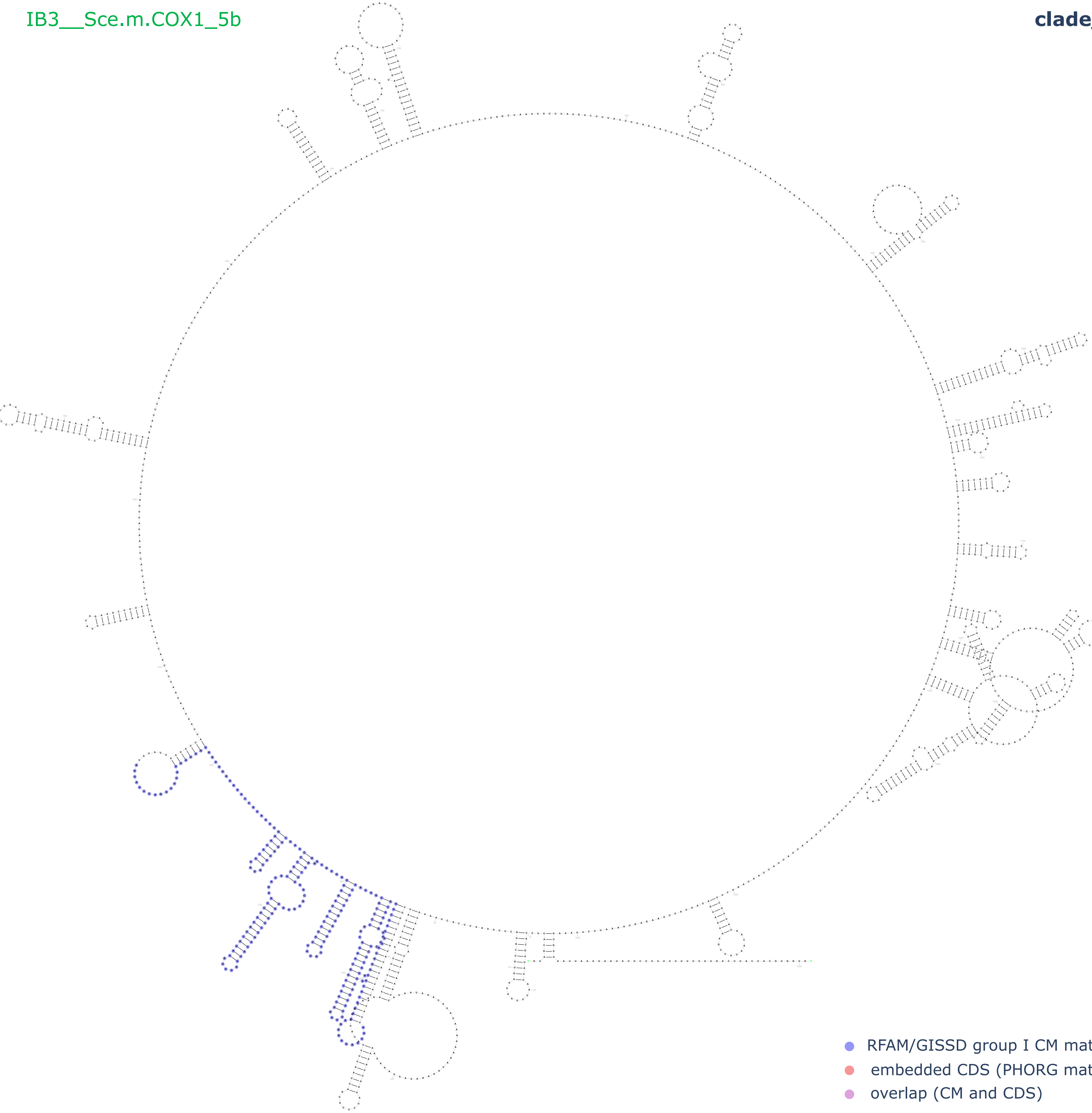

IB3__Sdo.m.COX1_4

clade_1

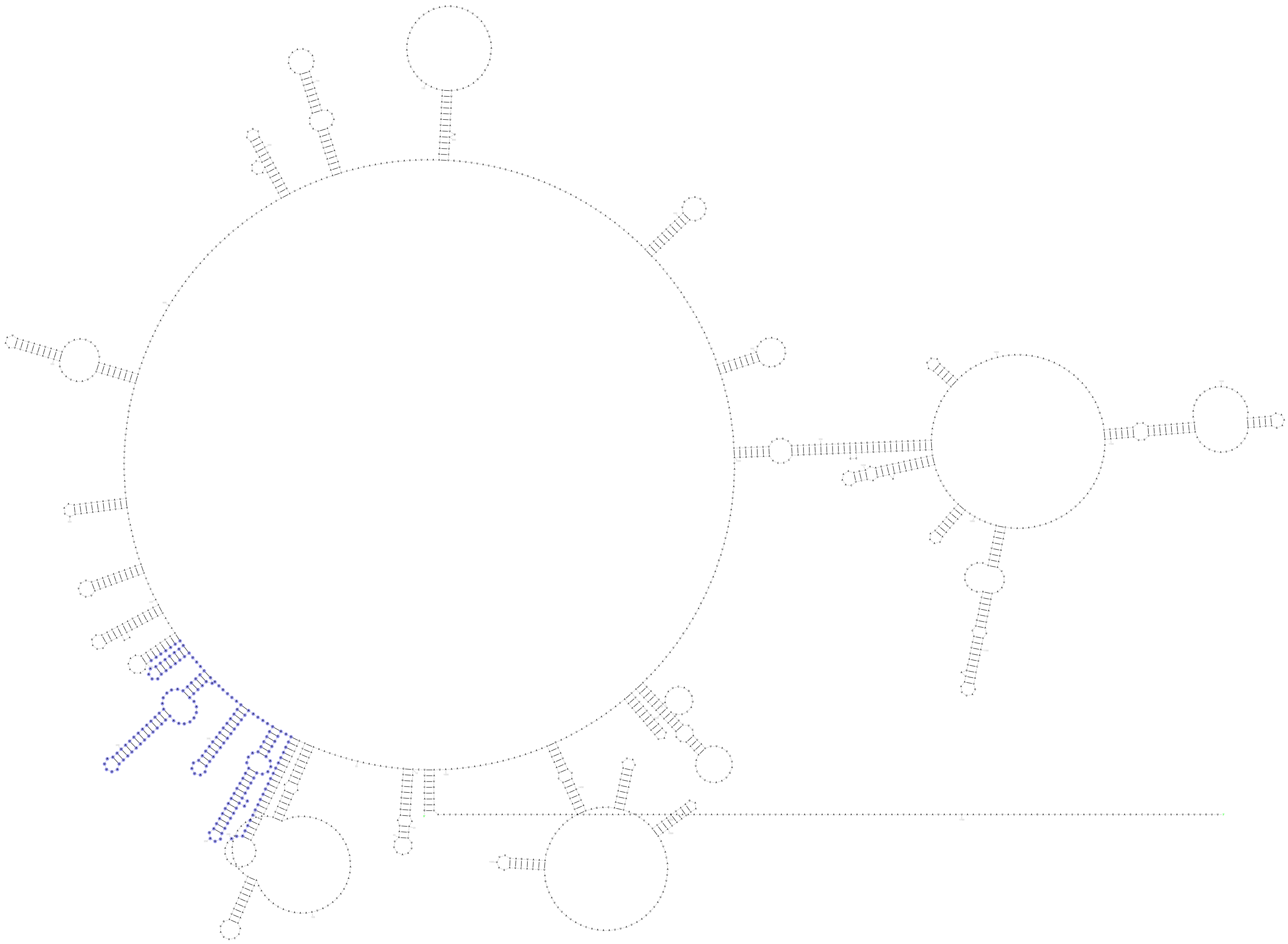

IB3__Spo.m.COX1_4

clade_1

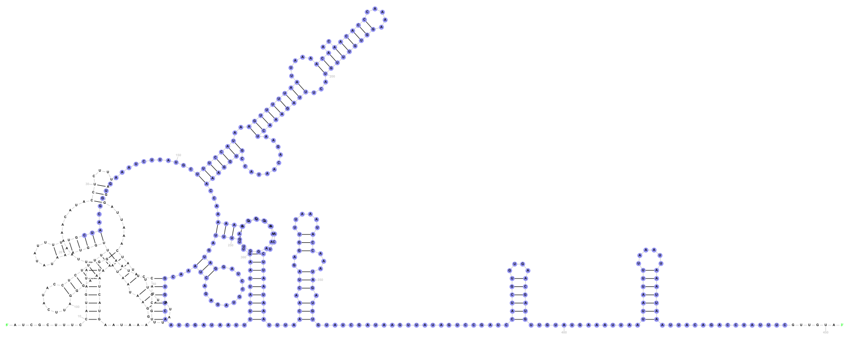

UNK__Bobb.TymS

clade_1

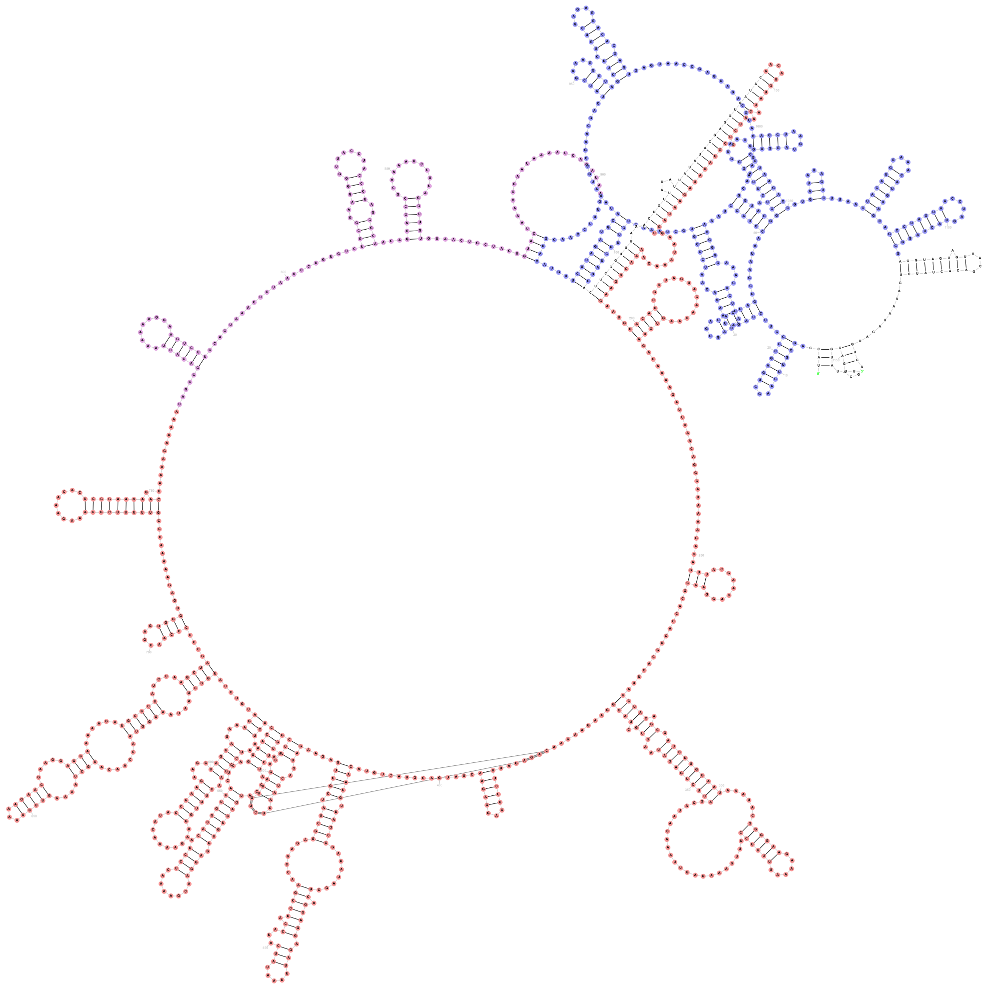

RFAM/GISSD group I CM match
embedded CDS (PHORG match)
overlap (CM and CDS)

IA2__T4.v.td_1

clade_1

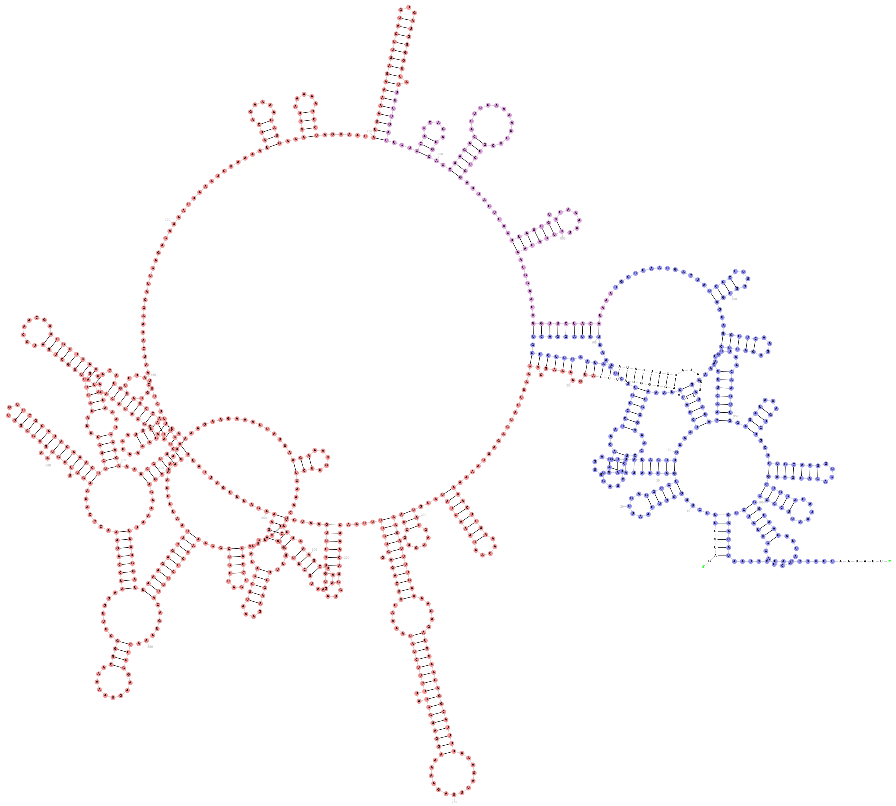

RFAM/GISSD group I CM match
embedded CDS (PHORG match)
overlap (CM and CDS)

IA2__Cre.c.psbA_3

clade_1

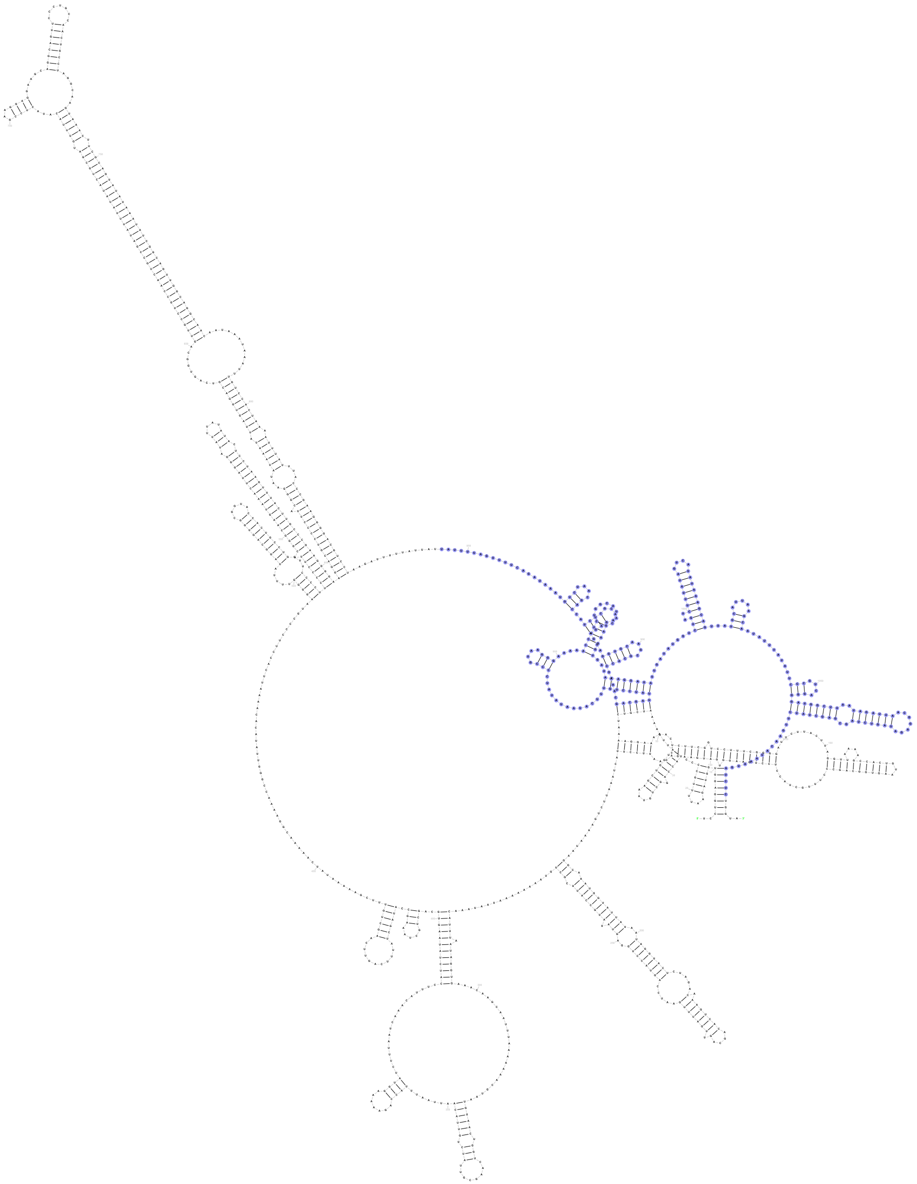

IC3__Bpl.c.trnL

clade_1

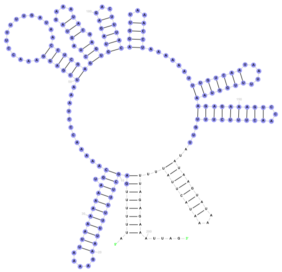

- RFAM/GISSD group I CM match
- embedded CDS (PHORG match)
- overlap (CM and CDS)

IA2__Twort.v.ORF142_2

clade_1

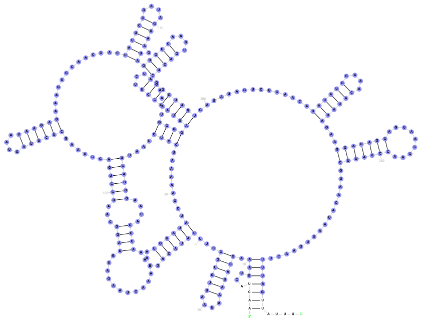

IA2__T4.v.nrdD_1

clade_2

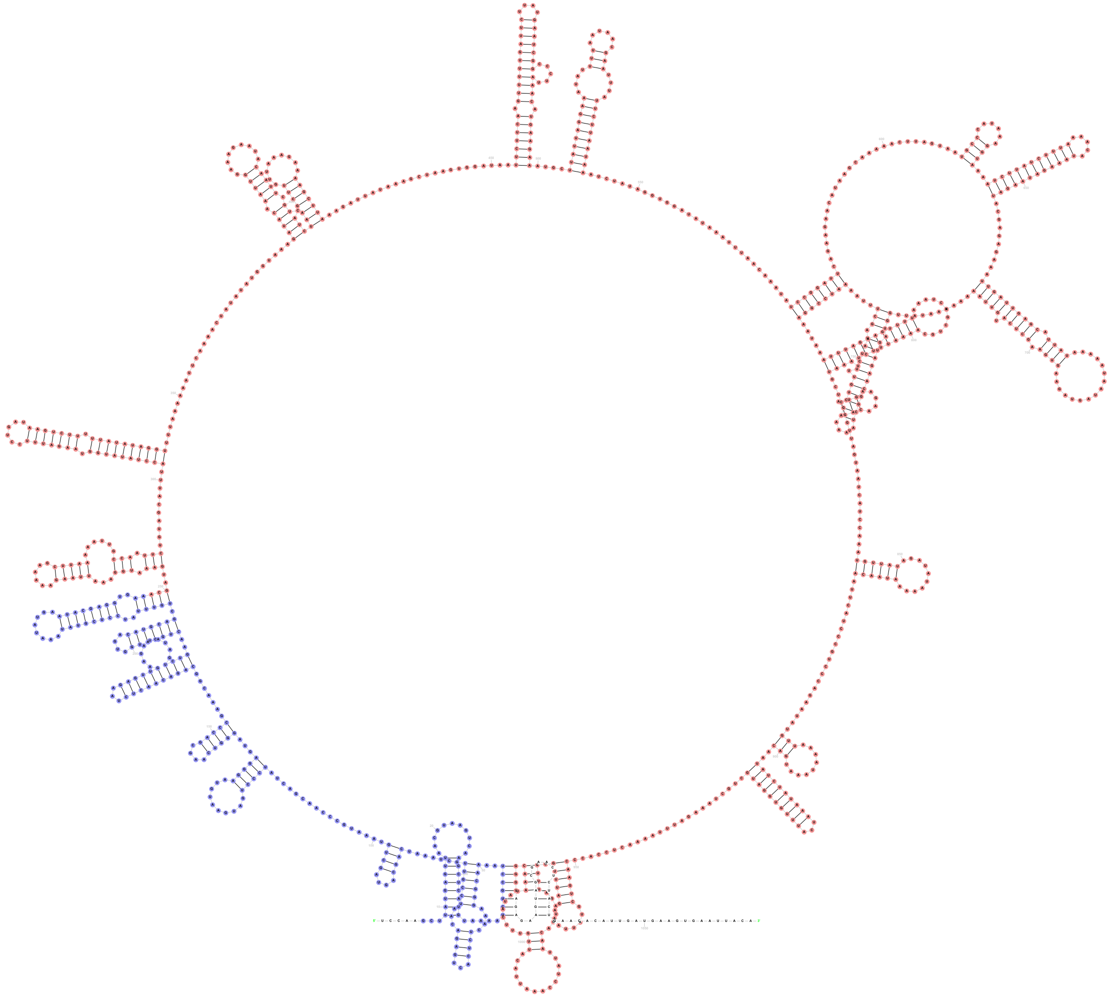

RFAM/GISSD group I CM match

embedded CDS (PHORG match)

overlap (CM and CDS)

ID__Pma.m.COB_1

clade_2

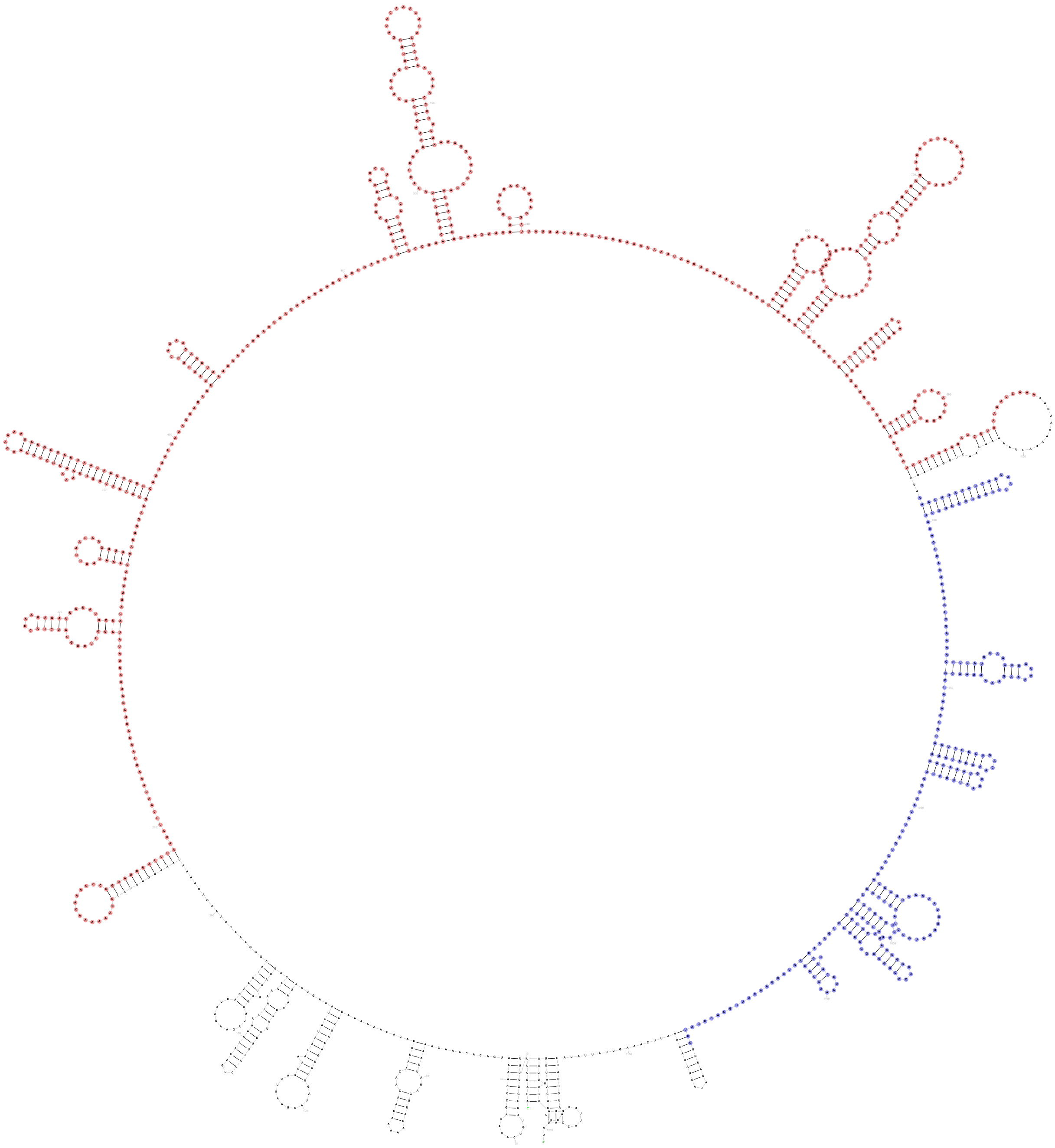

RFAM/GISSD group I CM match
embedded CDS (PHORG match)
overlap (CM and CDS)

ID__Sdo.m.COX1_2

clade_2

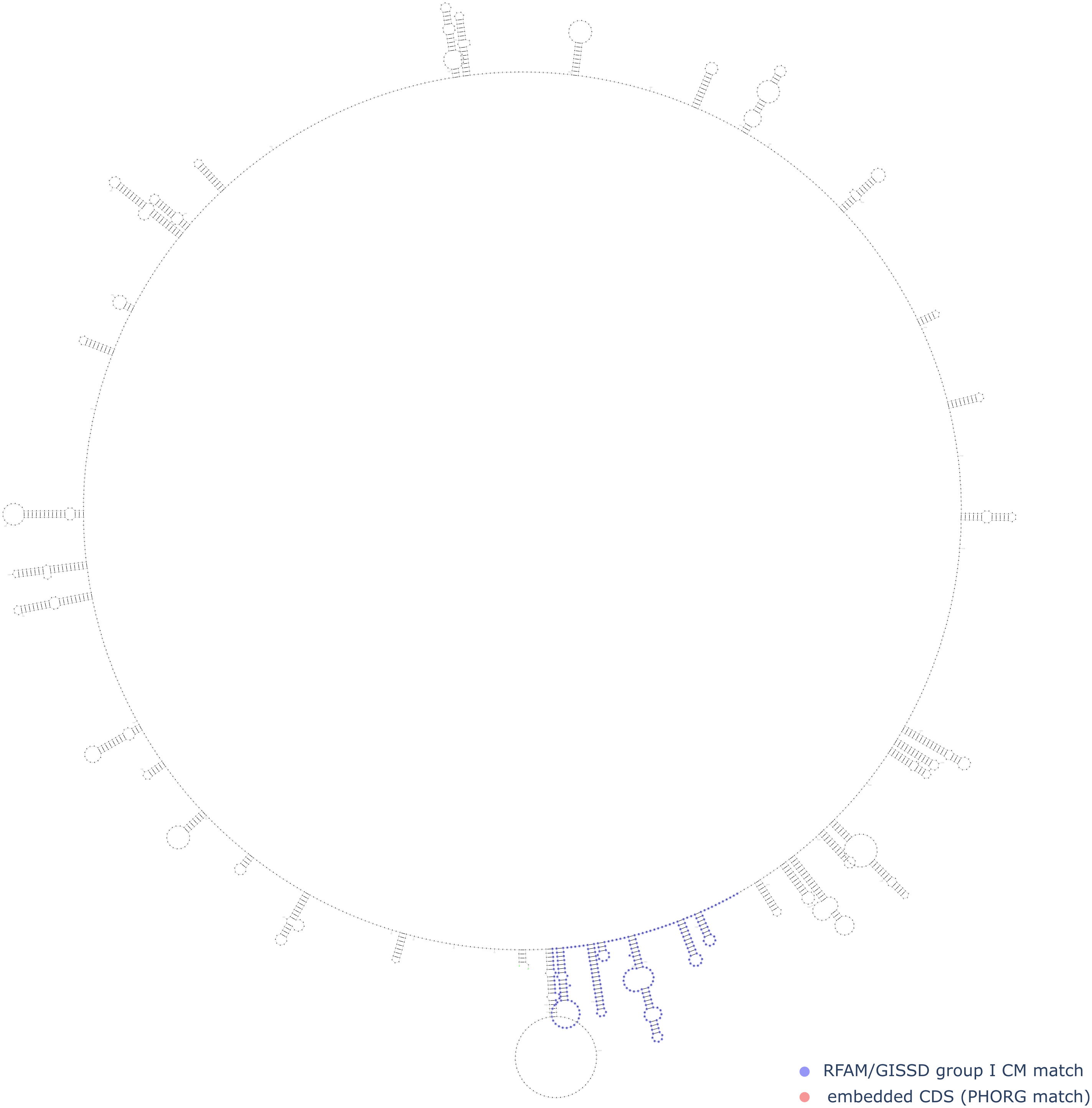

UNK__BCP8-2.TerL

clade_2

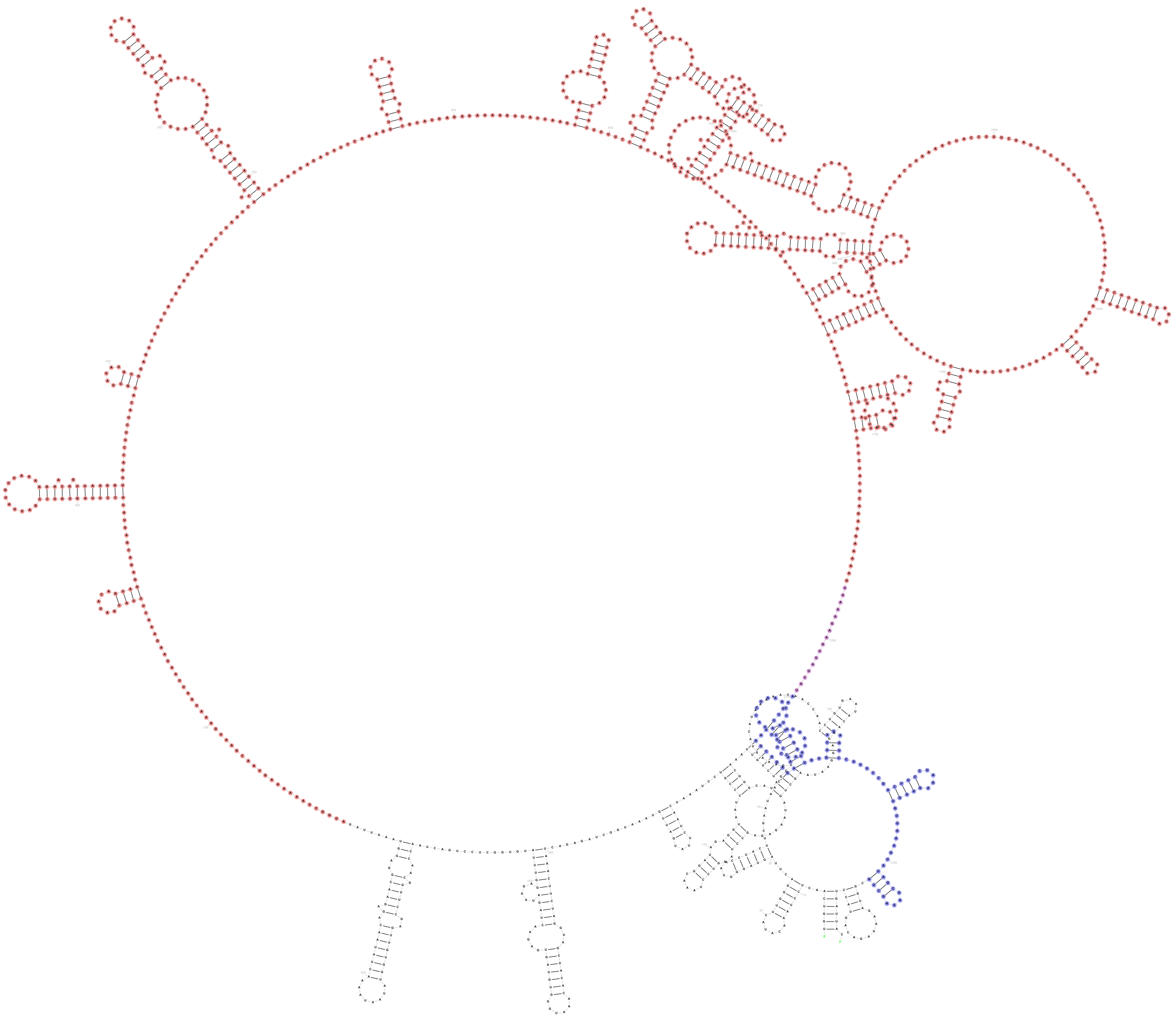

RFAM/GISSD group I CM match
embedded CDS (PHORG match)
overlap (CM and CDS)

UNK__phiAGATE.TerL-2

clade_2

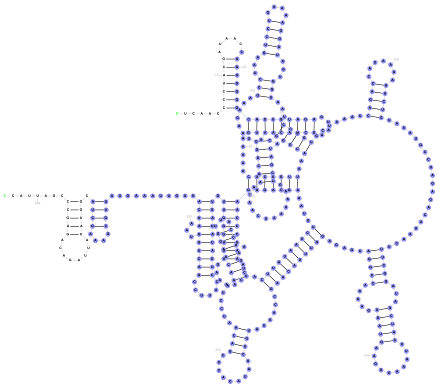

RFAM/GISSD group I CM match
embedded CDS (PHORG match)
overlap (CM and CDS)

UNK__SIOphi.TerL-2

clade_2

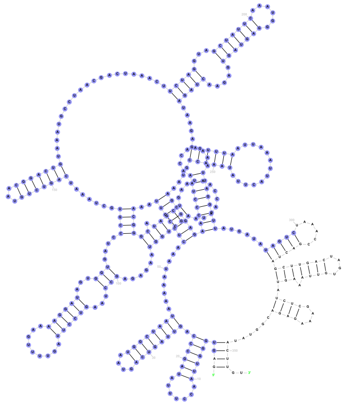

● RFAM/GISSD group I CM match
● embedded CDS (PHORG match)
● overlap (CM and CDS)

UNK__SIOphi.TerL

clade_2

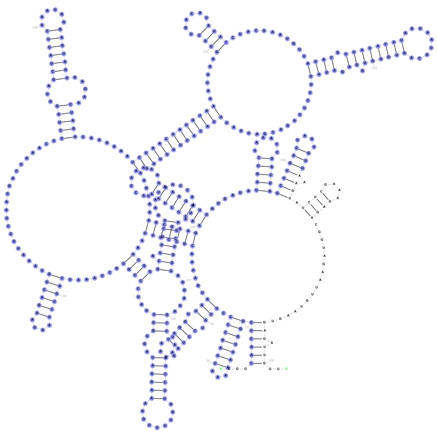

RFAM/GISSD group I CM match
embedded CDS (PHORG match)
overlap (CM and CDS)

UNK__Moonbeam.TerL

clade_2

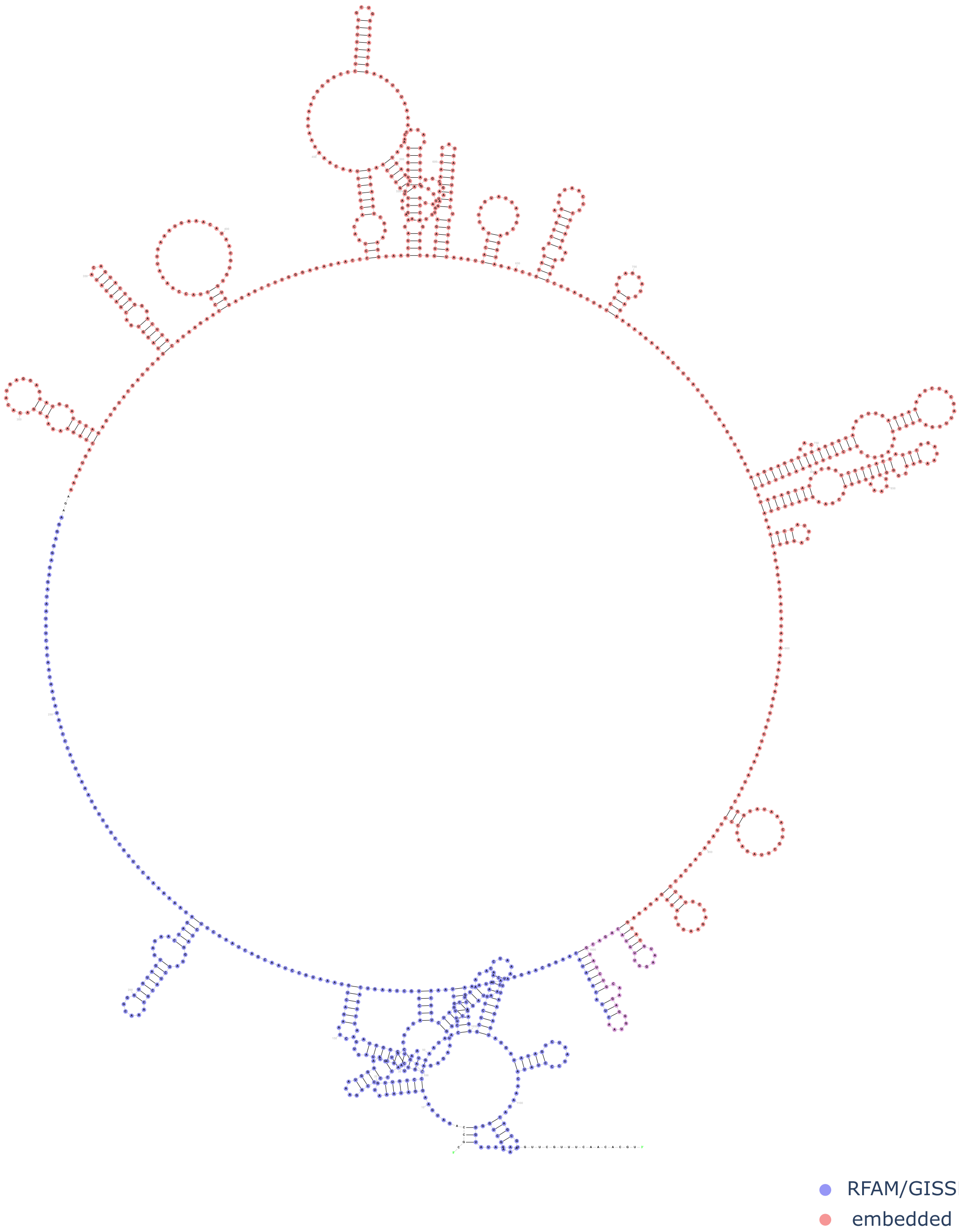

UNK__Moonbeam.Pol-2

clade_2

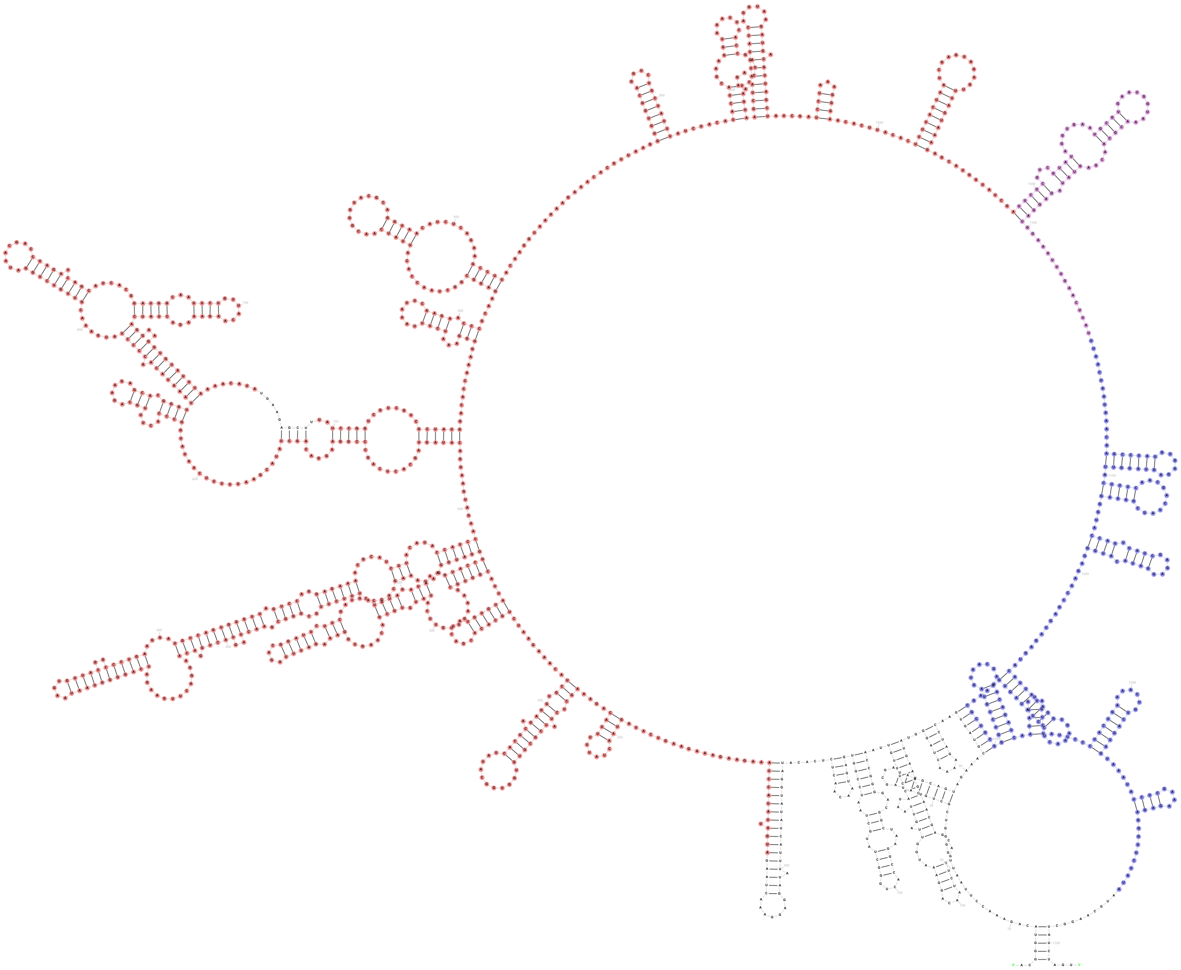

UNK__Grass.TerL

clade_3

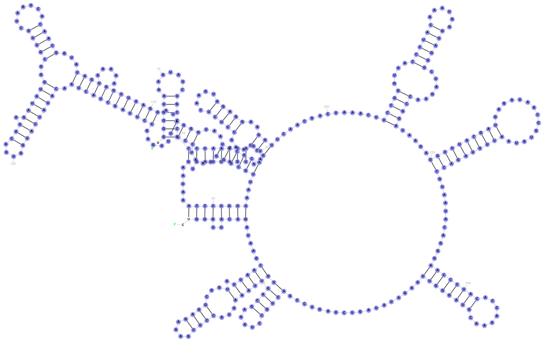

RFAM/GISSD group I CM match
embedded CDS (PHORG match)
overlap (CM and CDS)

UNK__SIOphi.TerL-3

clade_3

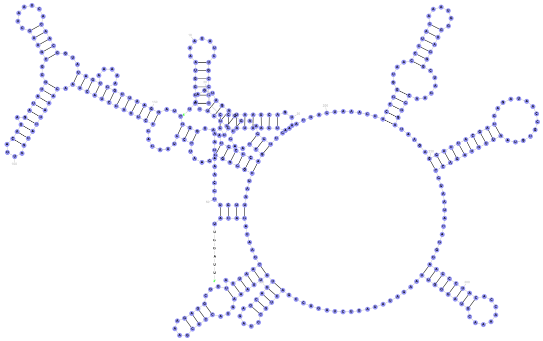

RFAM/GISSD group I CM match
embedded CDS (PHORG match)
overlap (CM and CDS)

UNK__SPG24.TerL

clade_3

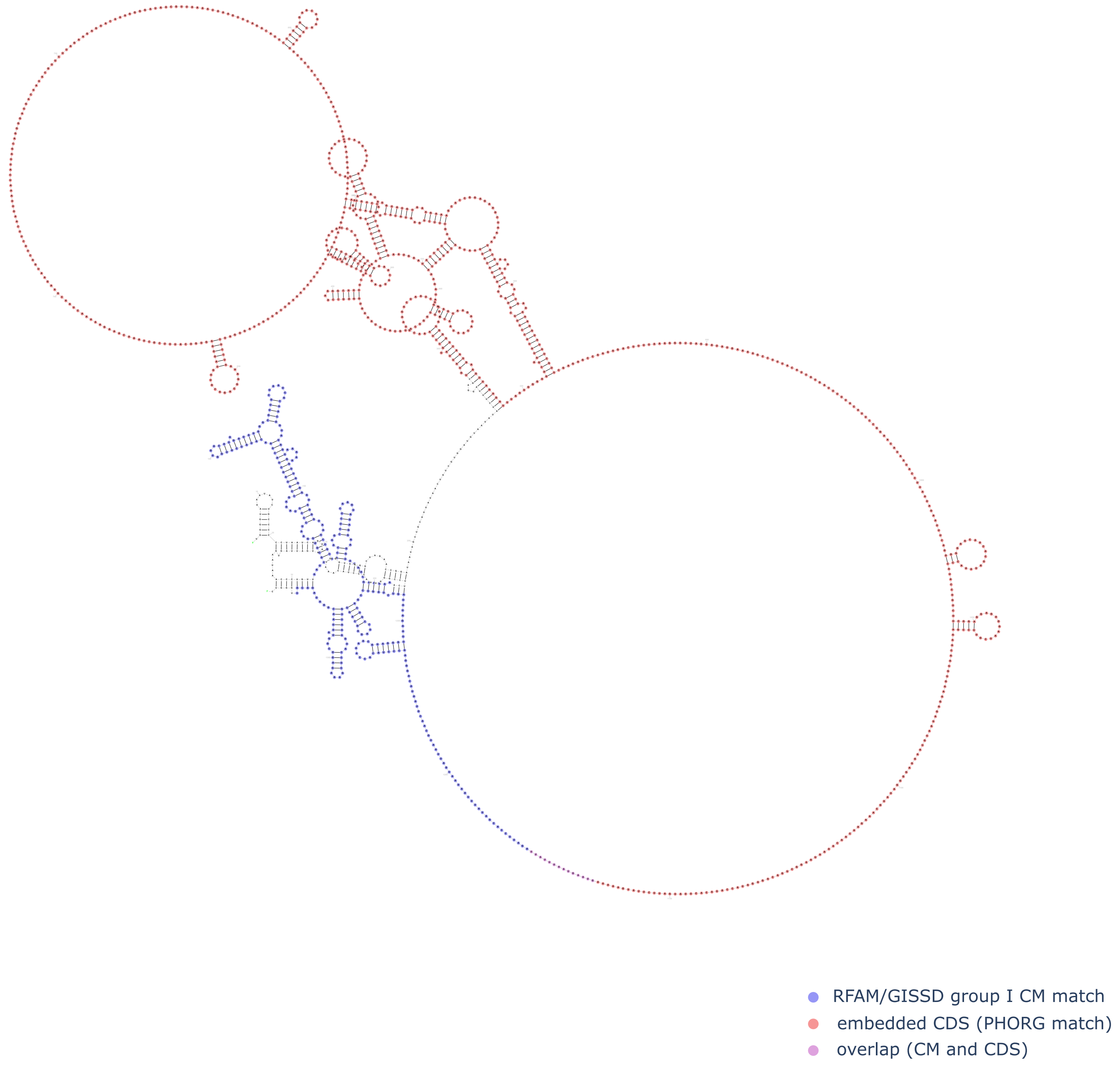

IA2__RB3.v.nrdB_1

clade_4

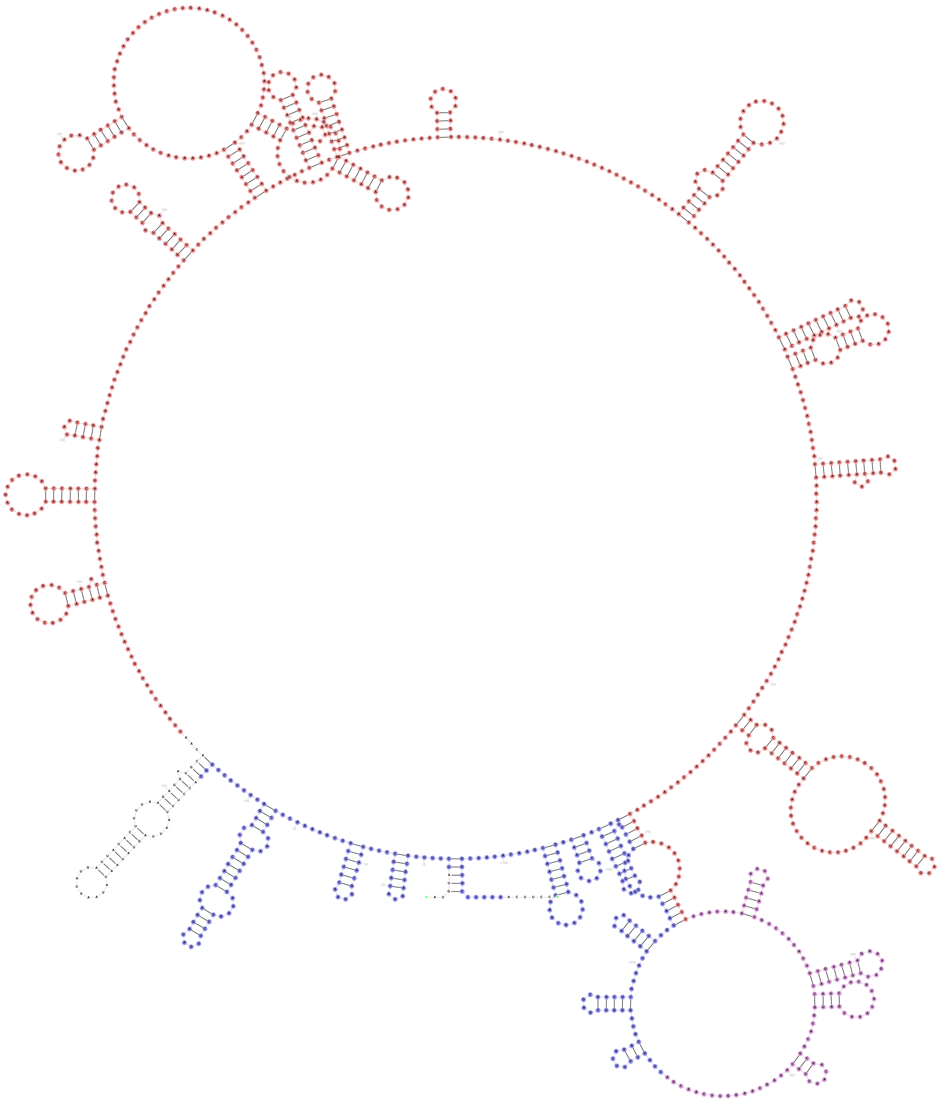

IA2__T4.v.nrdB_1

clade_4

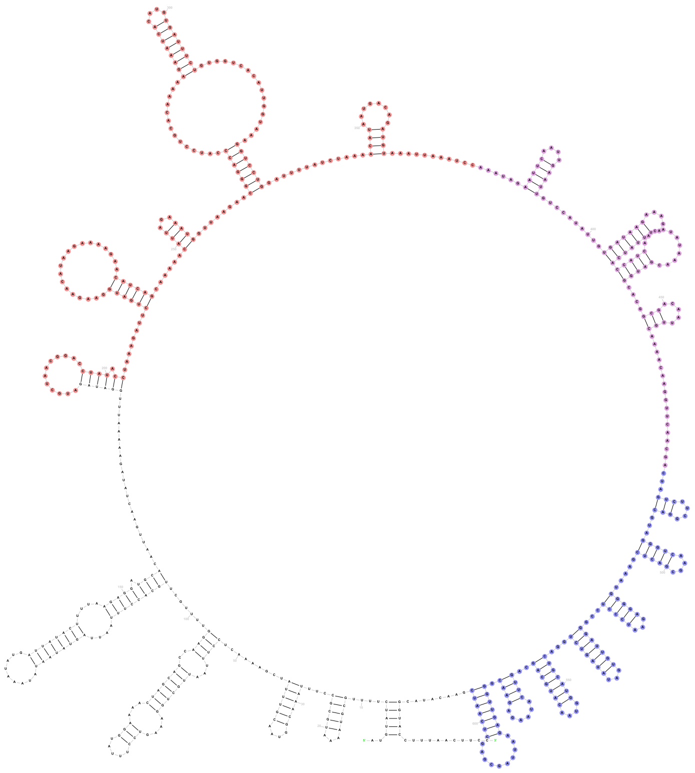

UNK__phiAGATE.TerL

clade_4

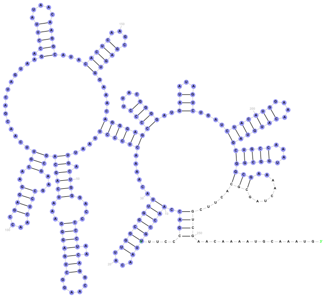

- RFAM/GISSD group I CM match
- embedded CDS (PHORG match)
- overlap (CM and CDS)

UNK__Bobb.rNDPL

clade_4

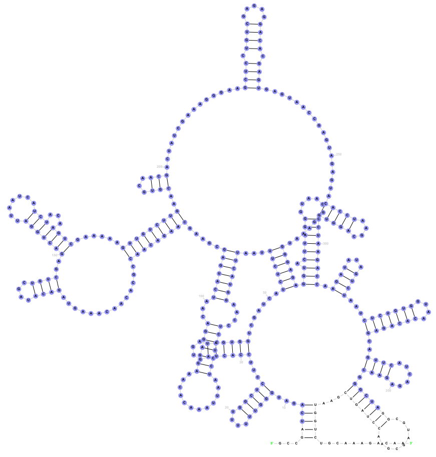

RFAM/GISSD group I CM match
embedded CDS (PHORG match)
overlap (CM and CDS)

UNK__phiAGATE.rNDPL

clade_4

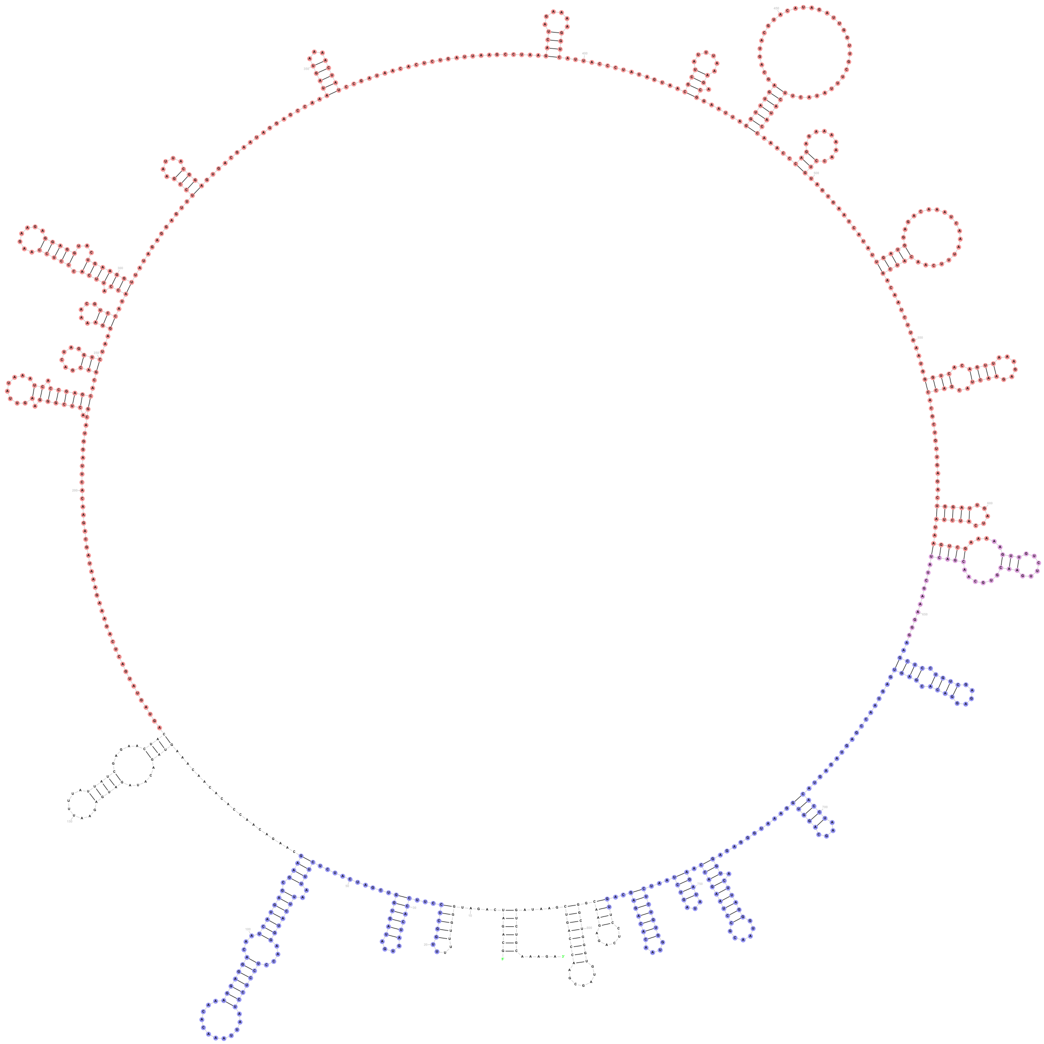

RFAM/GISSD group I CM match
embedded CDS (PHORG match)
overlap (CM and CDS)

UNK__phiNIT1.rNDPL

clade_4

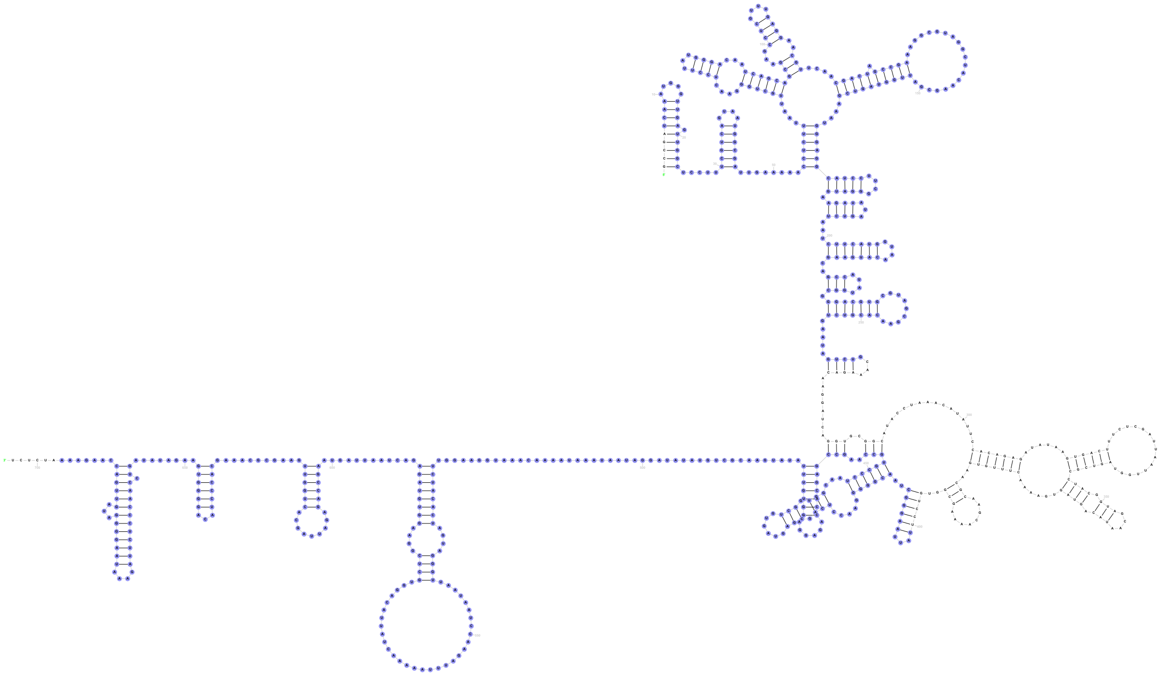

- RFAM/GISSD group I CM match
- embedded CDS (PHORG match)
- overlap (CM and CDS)

clade_4

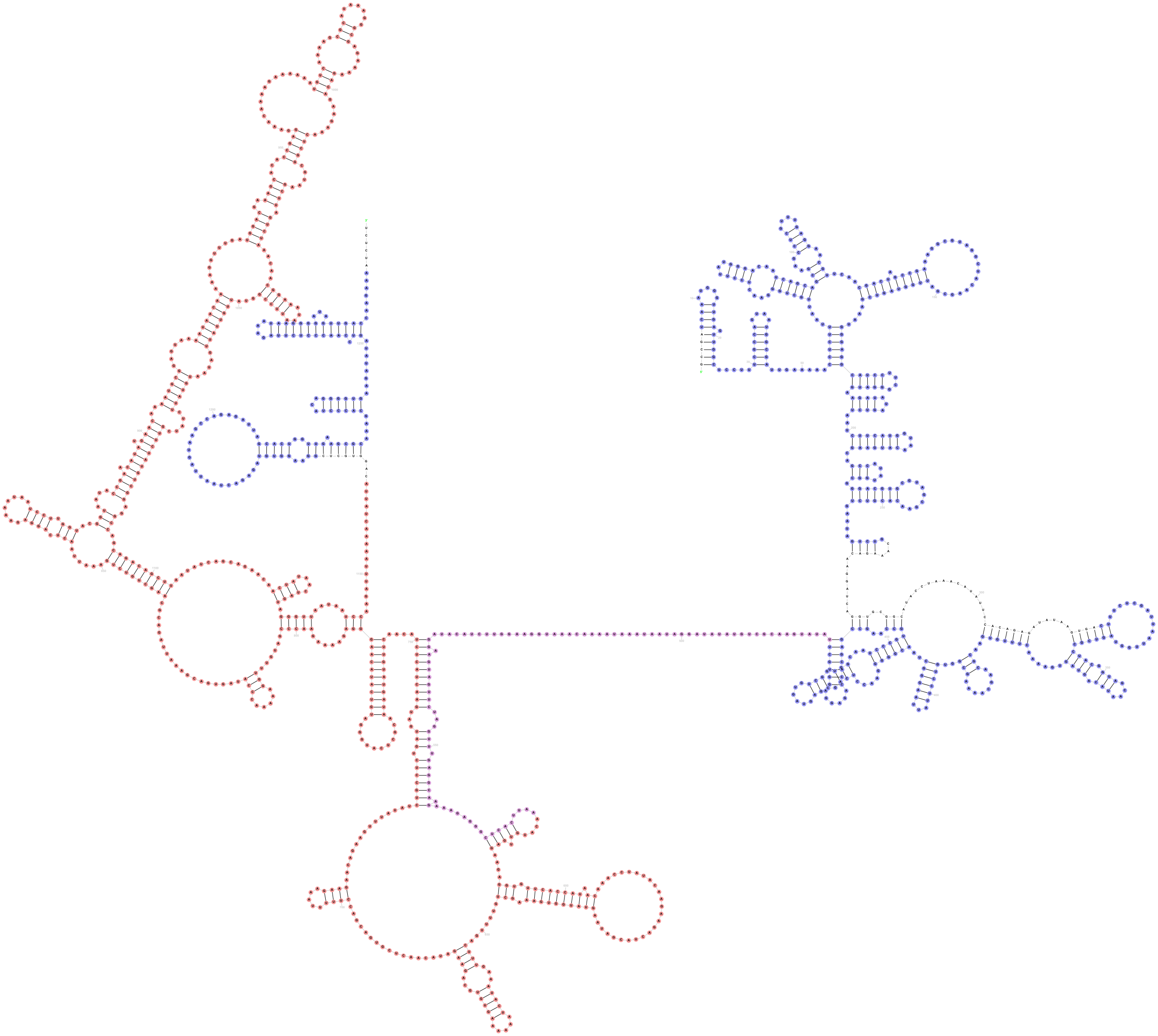

RFAM/GISSD group I CM match
embedded CDS (PHORG match)
overlap (CM and CDS)

IA2__Beta-22.v.td_1

clade_5

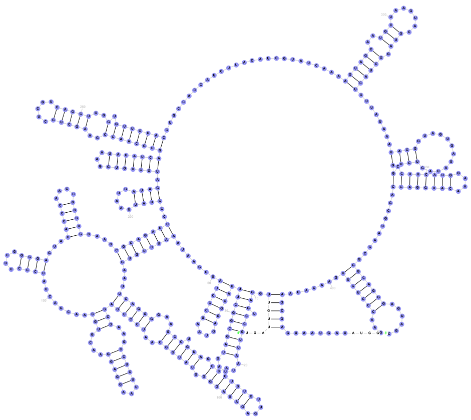

RFAM/GISSD group I CM match
embedded CDS (PHORG match)
overlap (CM and CDS)

IA2__Phi-E.v.DP_1

clade_5

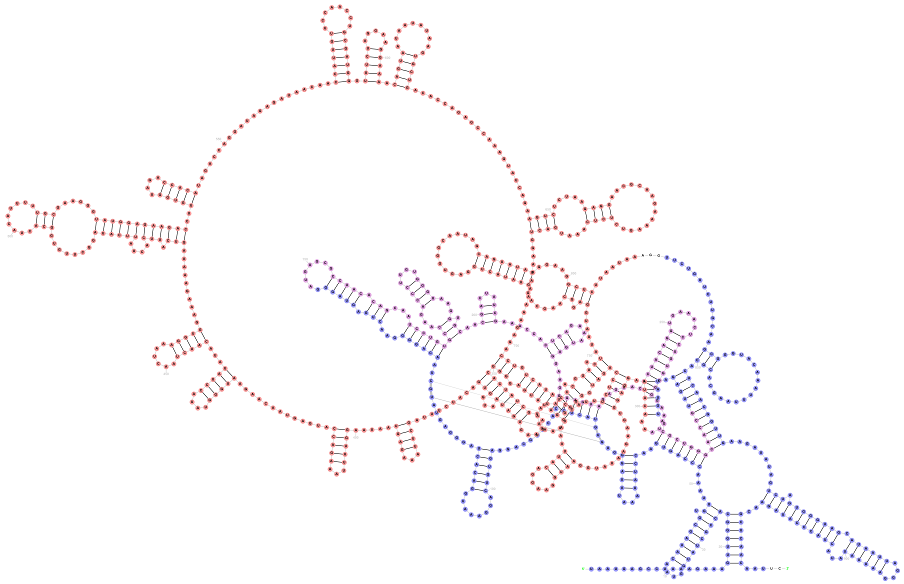

RFAM/GISSD group I CM match
embedded CDS (PHORG match)
overlap (CM and CDS)

IA2__SP82.v.DP_1

clade_5

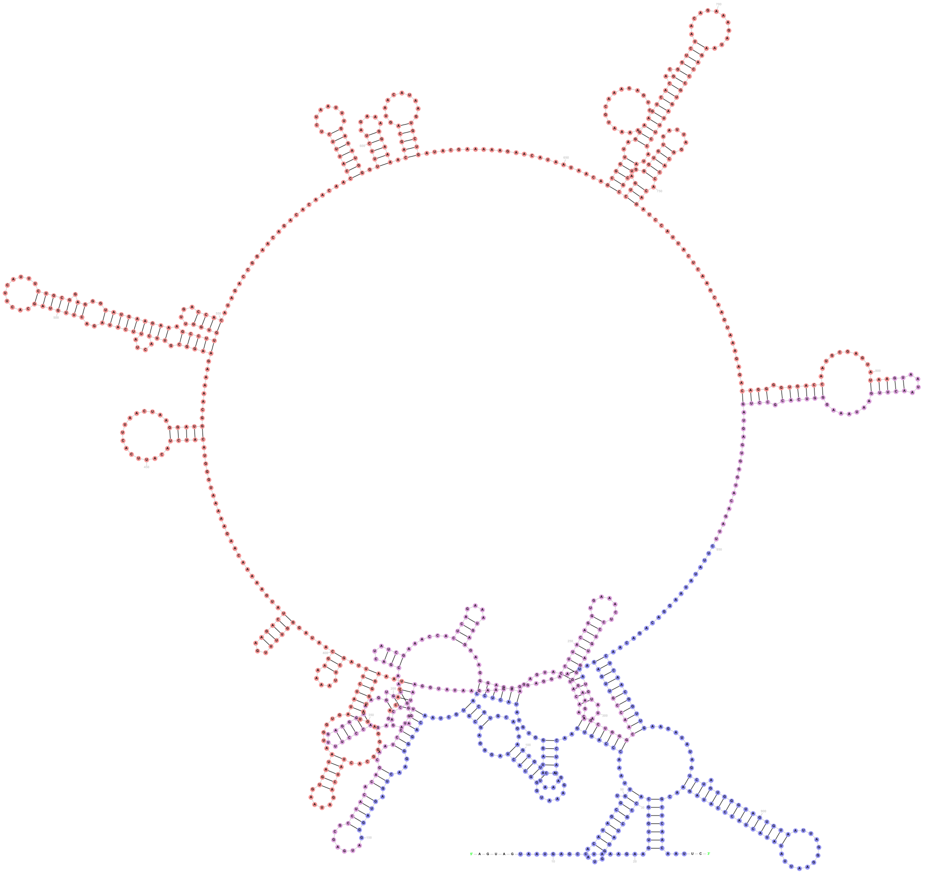

IA2__SPO1.v.DP_1

clade_5

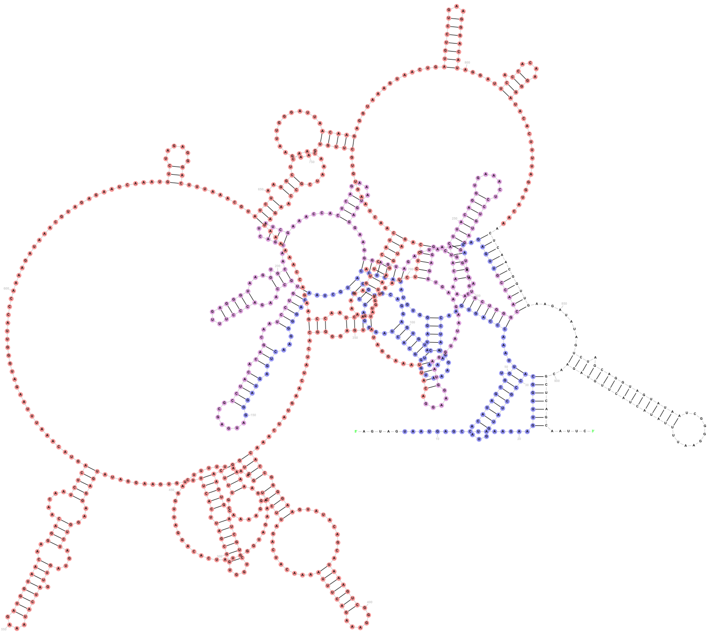

- RFAM/GISSD group I CM match
- embedded CDS (PHORG match)
- overlap (CM and CDS)

UNK__Eldridge.rNDPs

clade_5

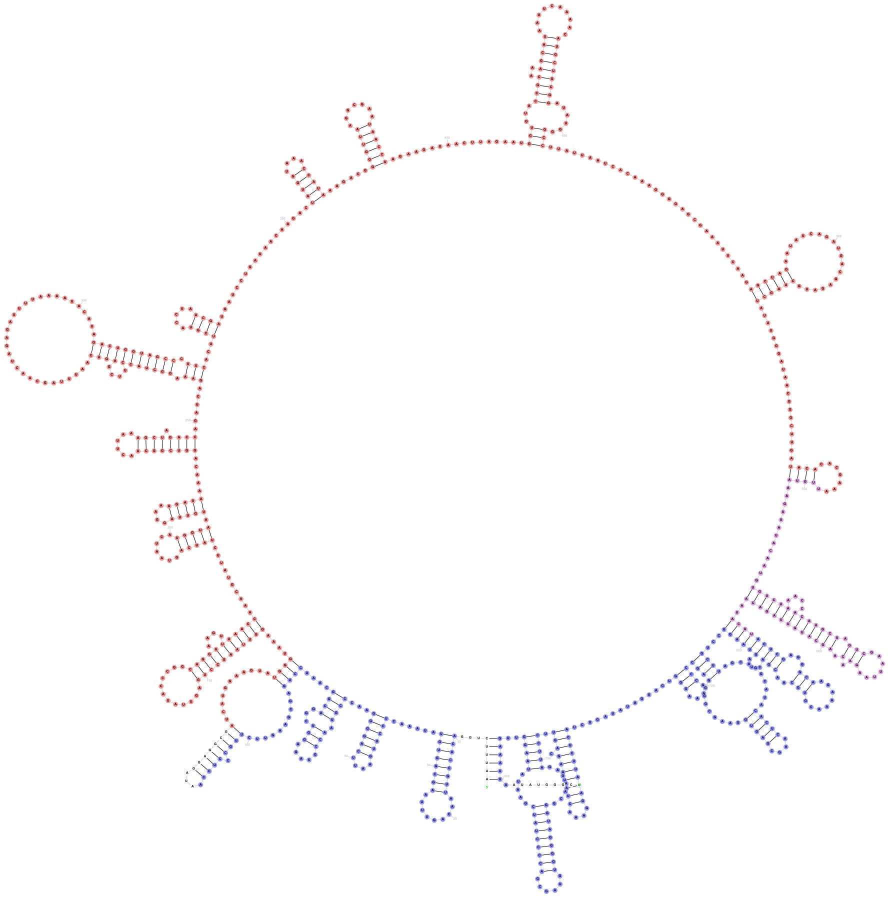

UNK__Moonbeam.TerL-2

clade_6

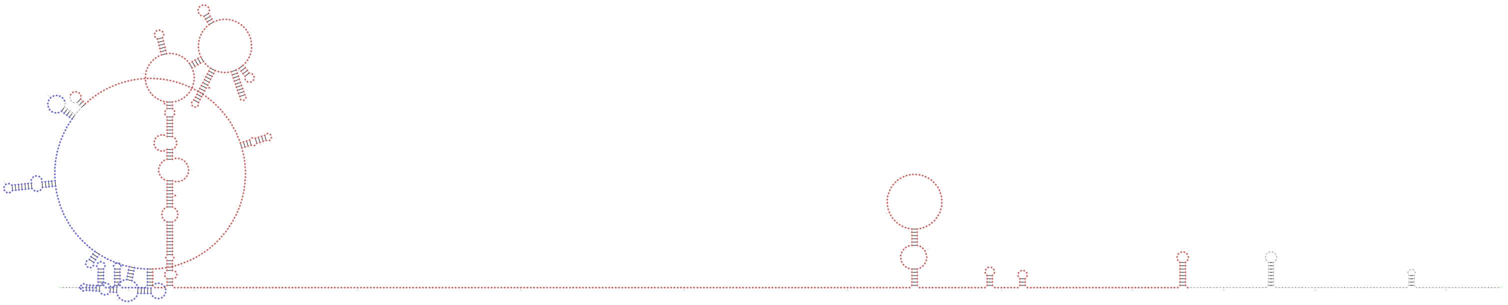

UNK__SIOphi.Pol-3

clade_6

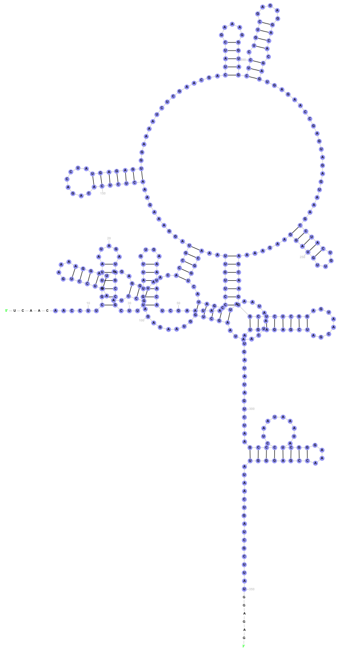

RFAM/GISSD group I CM match
embedded CDS (PHORG match)
overlap (CM and CDS)

IA3__Cac.cS508

clade_7

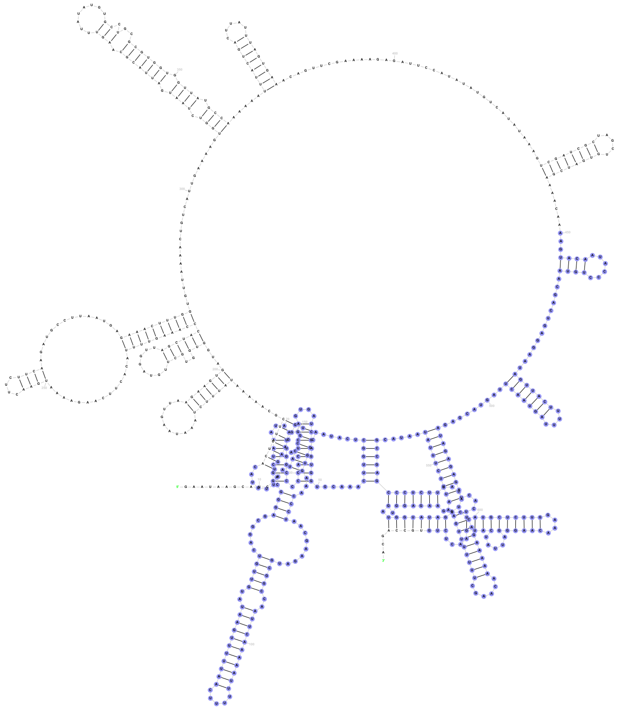

IA3__Cse.cS508

clade_7

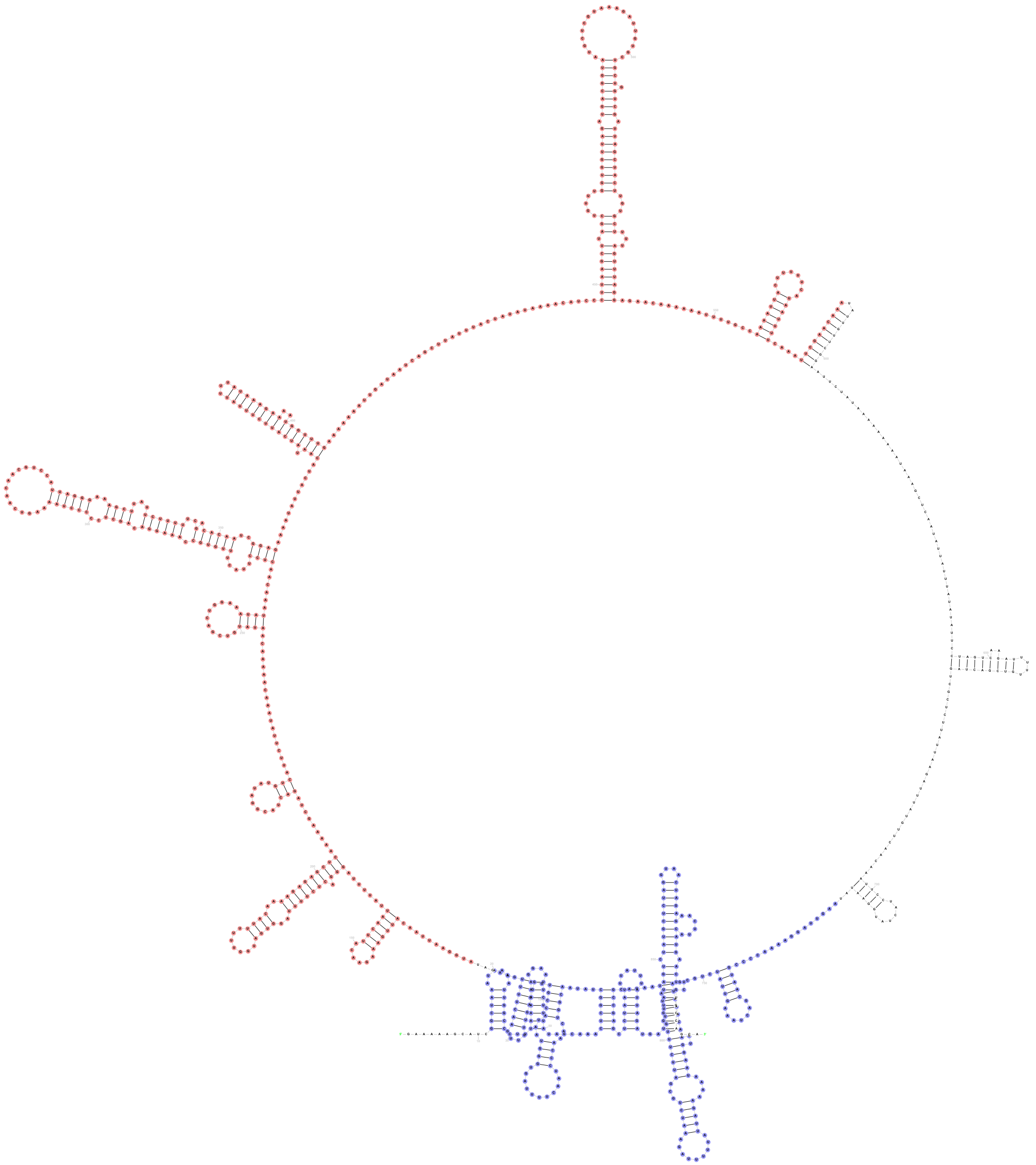

RFAM/GISSD group I CM match
embedded CDS (PHORG match)
overlap (CM and CDS)

IA3__Cha_vu.mL779

clade_7

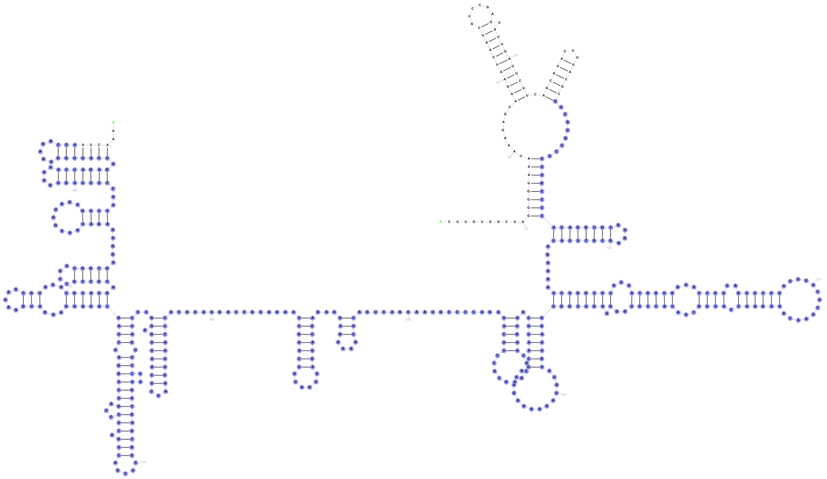

RFAM/GISSD group I CM match
embedded CDS (PHORG match)
overlap (CM and CDS)

IA3__Tag.cL2596

clade_7

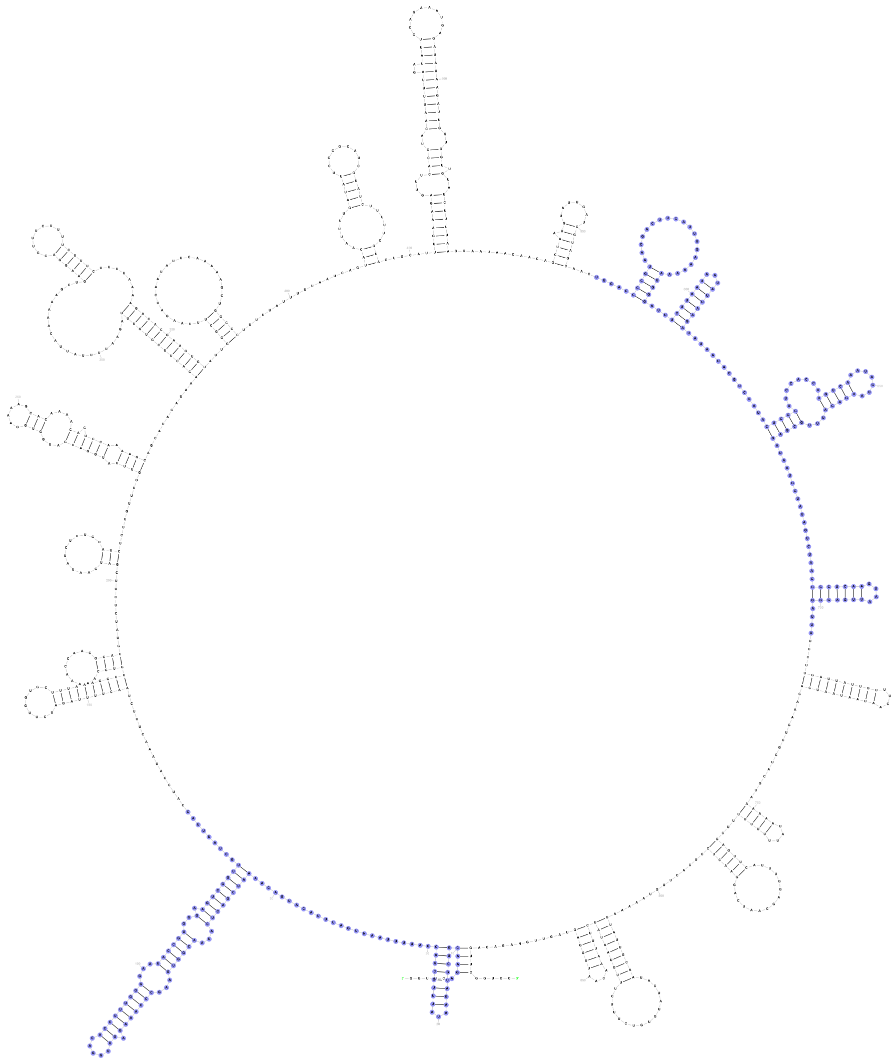

IA3__Cpa.cS508

clade_7

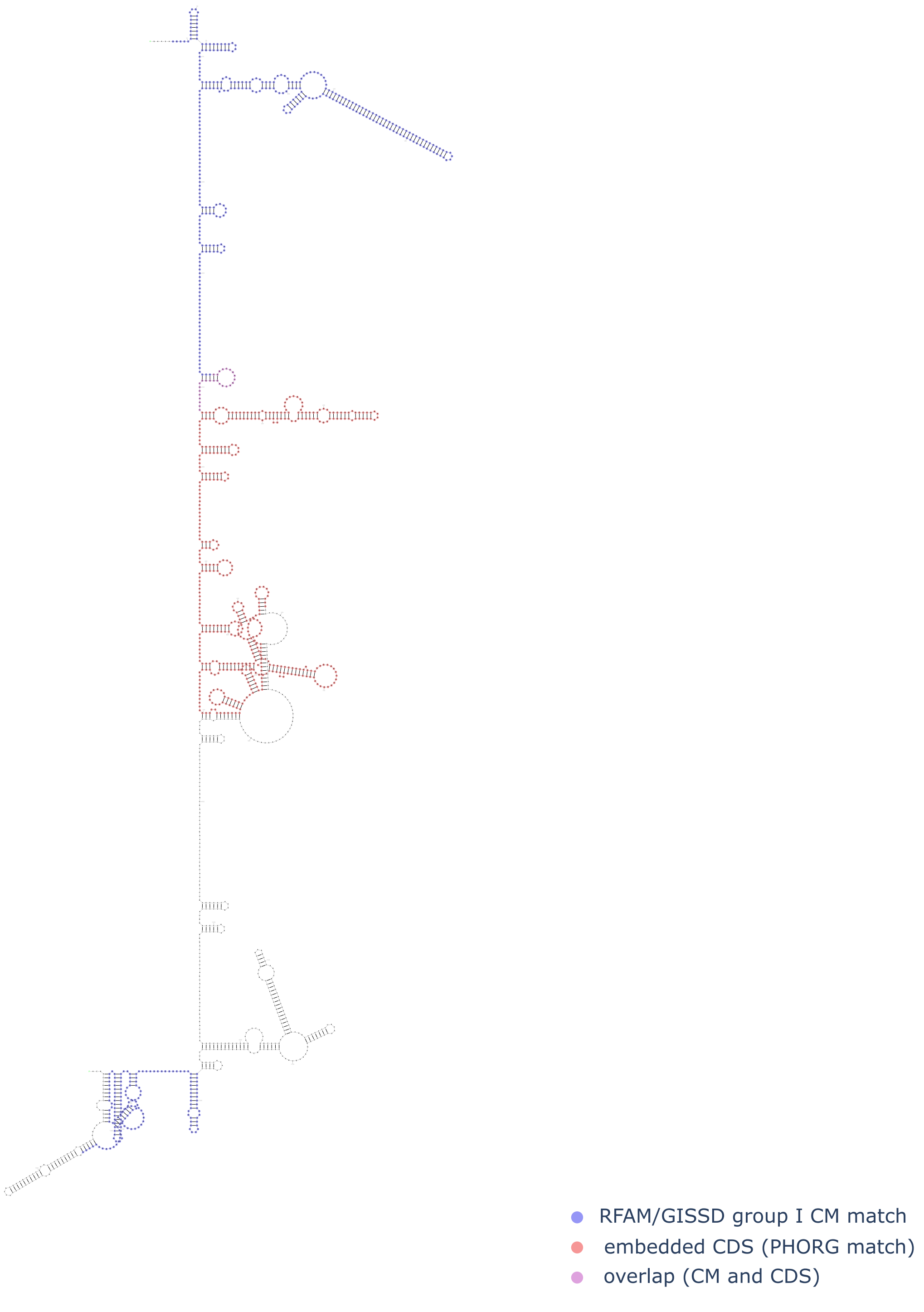

UNK__Grass.Pol-2

clade_7

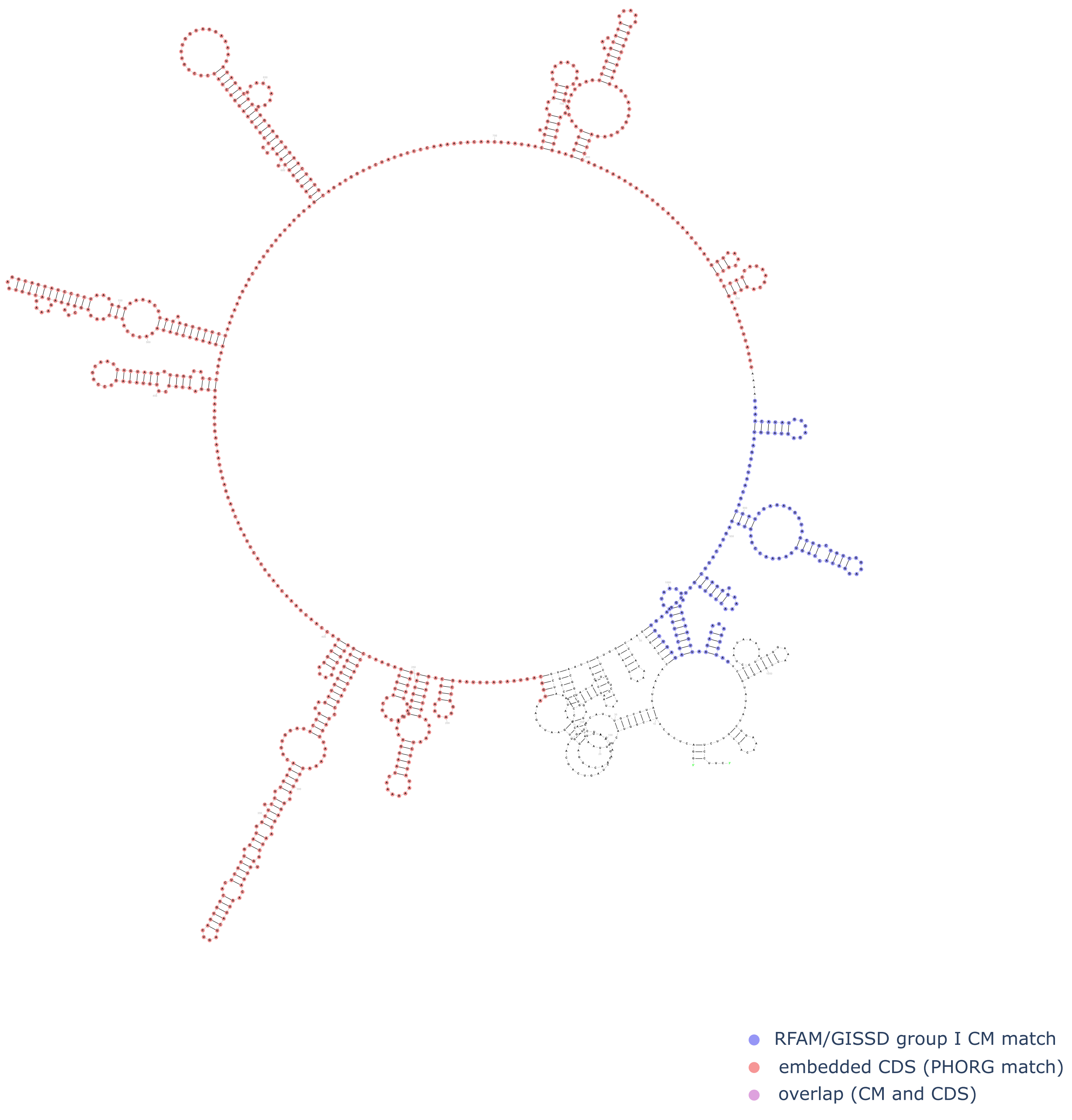

UNK__phiAGATE.Pol

clade_7

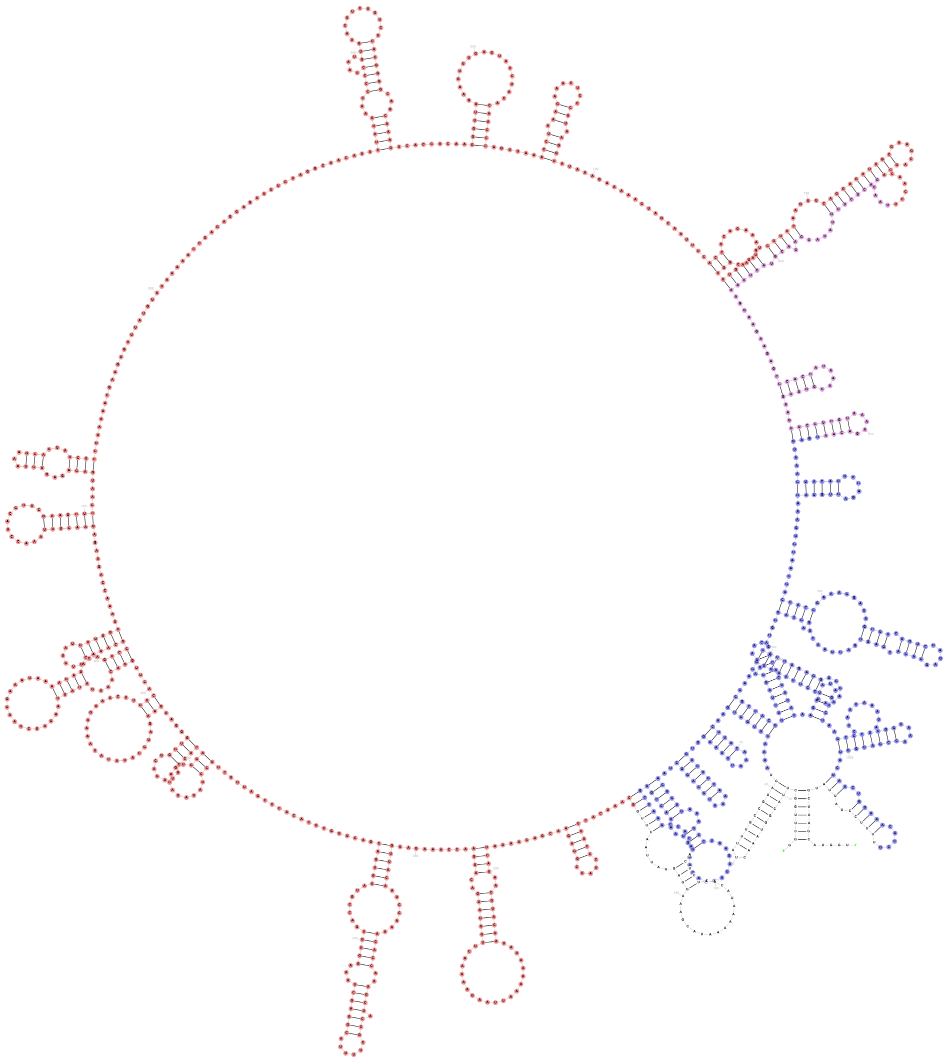

UNK__Kida.Pol

clade_7

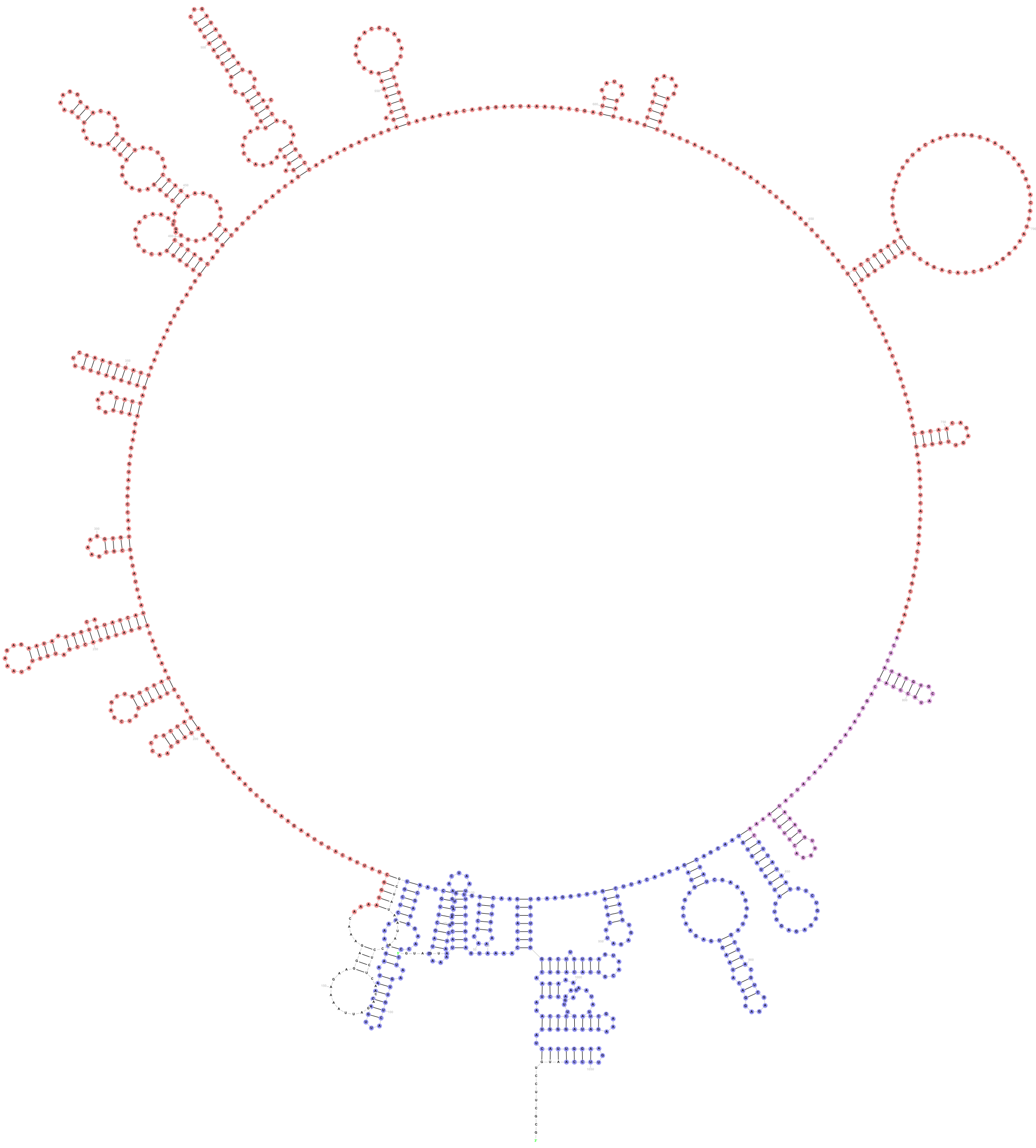

RFAM/GISSD group I CM match
embedded CDS (PHORG match)
overlap (CM and CDS)

UNK__Nemo.Pol

clade_7

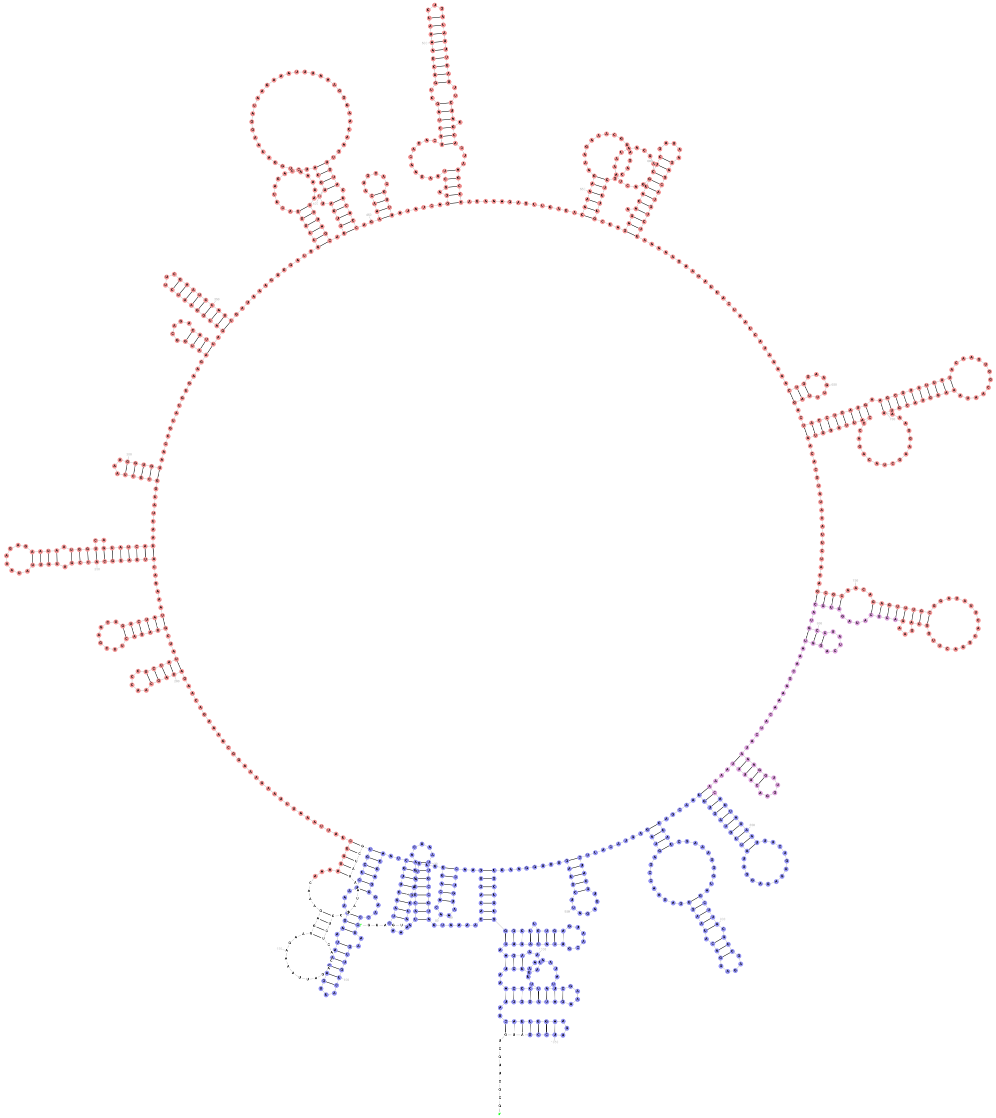

RFAM/GISSD group I CM match
embedded CDS (PHORG match)
overlap (CM and CDS)

UNK__KNotTheCreek.Polr

clade_7

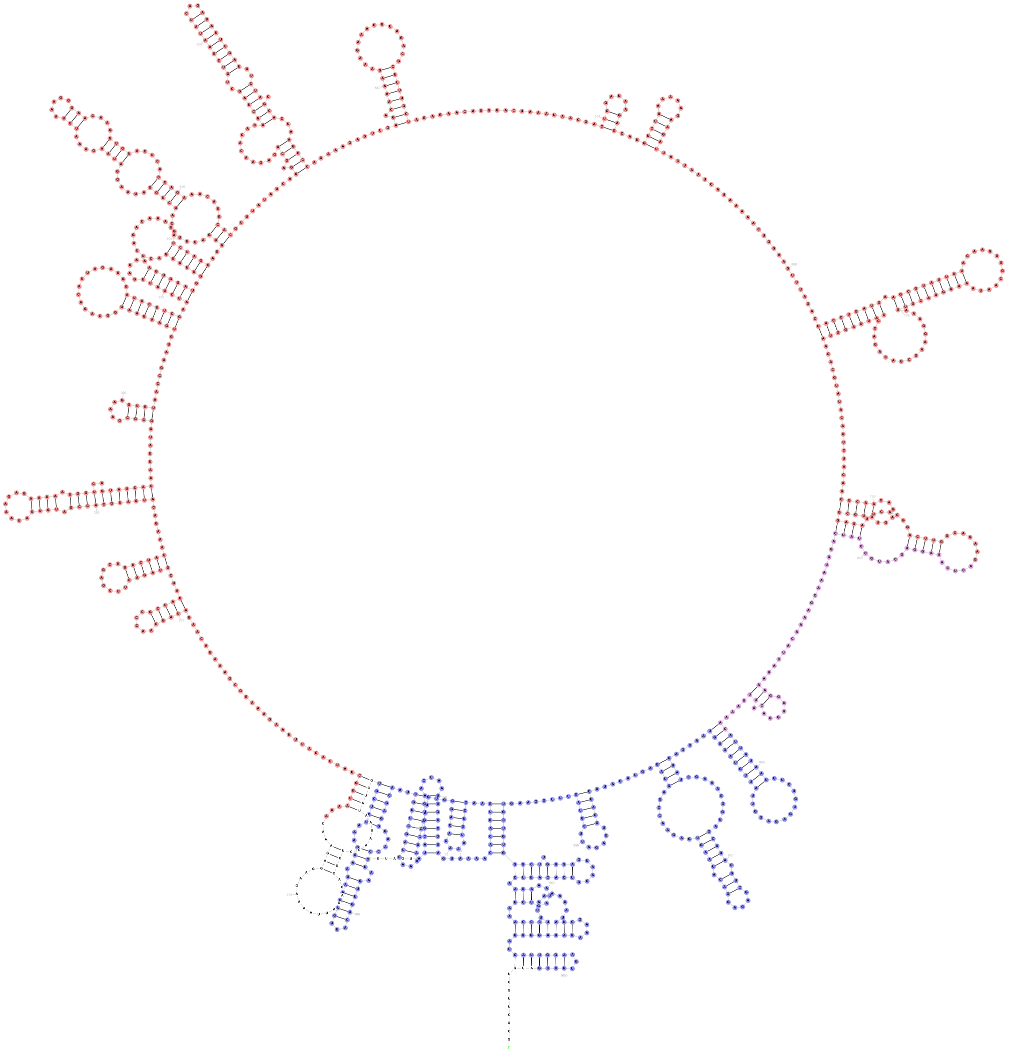

IA3__Aca.mL1951

clade_8

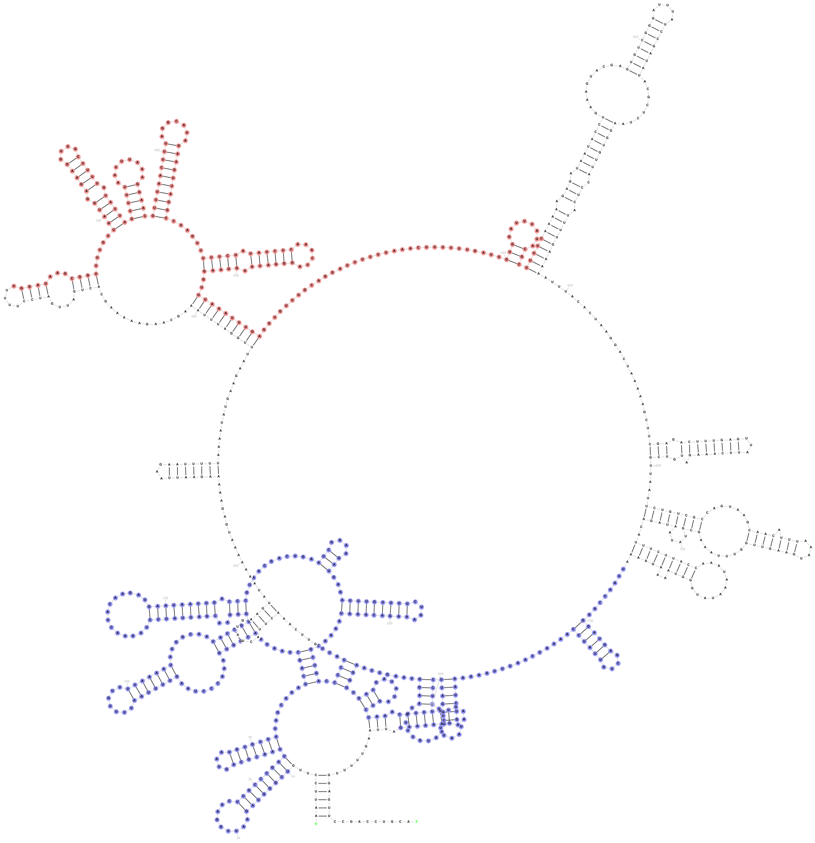

RFAM/GISSD group I CM match
embedded CDS (PHORG match)
overlap (CM and CDS)

IA3__Chl_vu.mL1951

clade_8

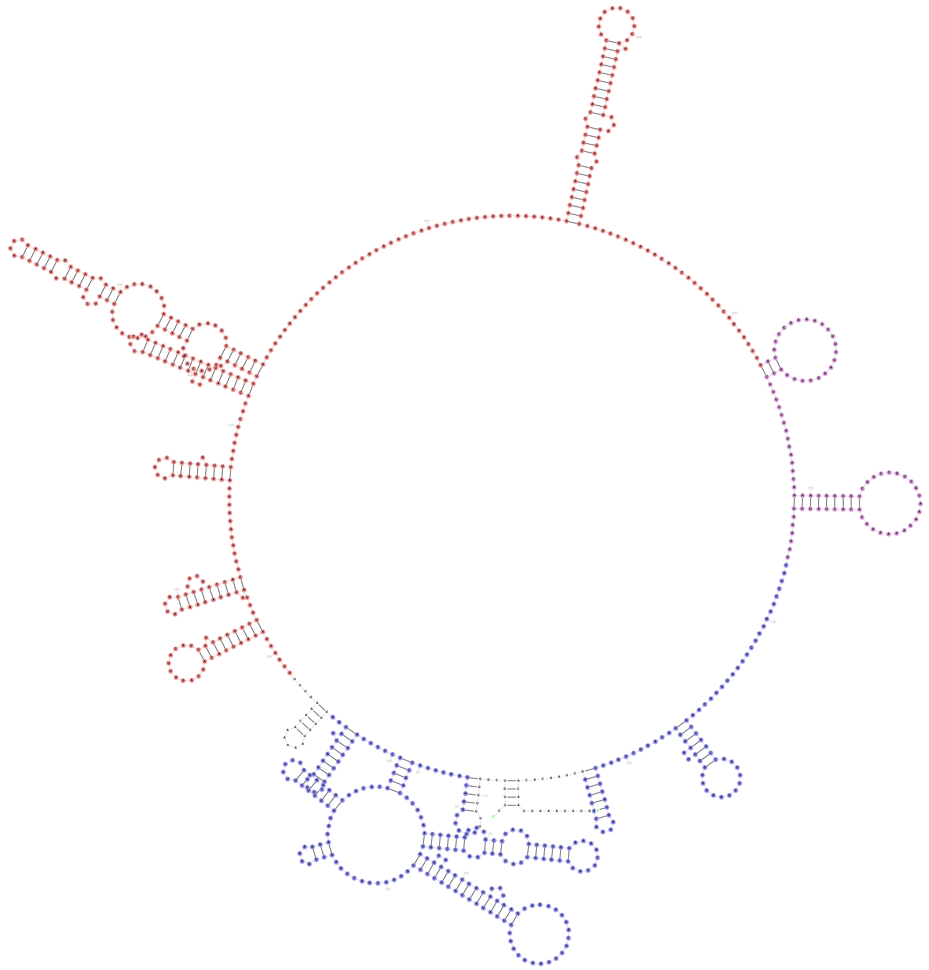

IA3__Cmo.cS531

clade_8

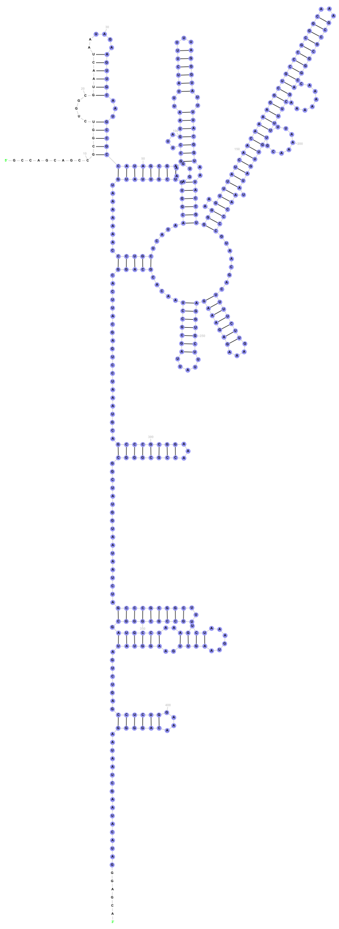

- RFAM/GISSD group I CM match
- embedded CDS (PHORG match)
- overlap (CM and CDS)

IA3__She.cL730

clade_8

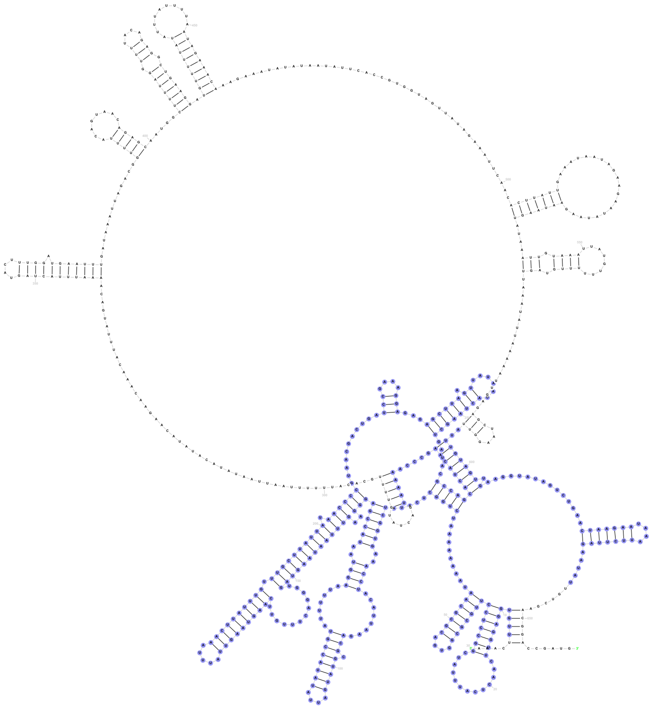

RFAM/GISSD group I CM match
embedded CDS (PHORG match)
overlap (CM and CDS)

IA3__Msp-1.cL1951

clade_8

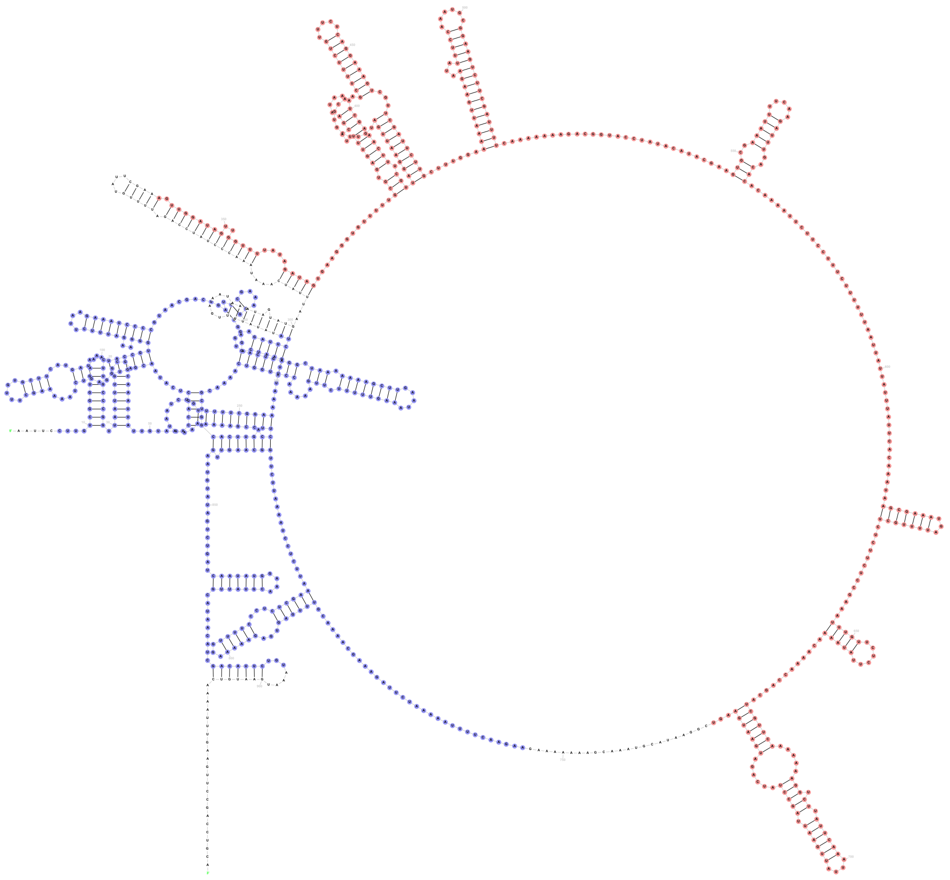

IA3__Msp-2.cL1951

clade_8

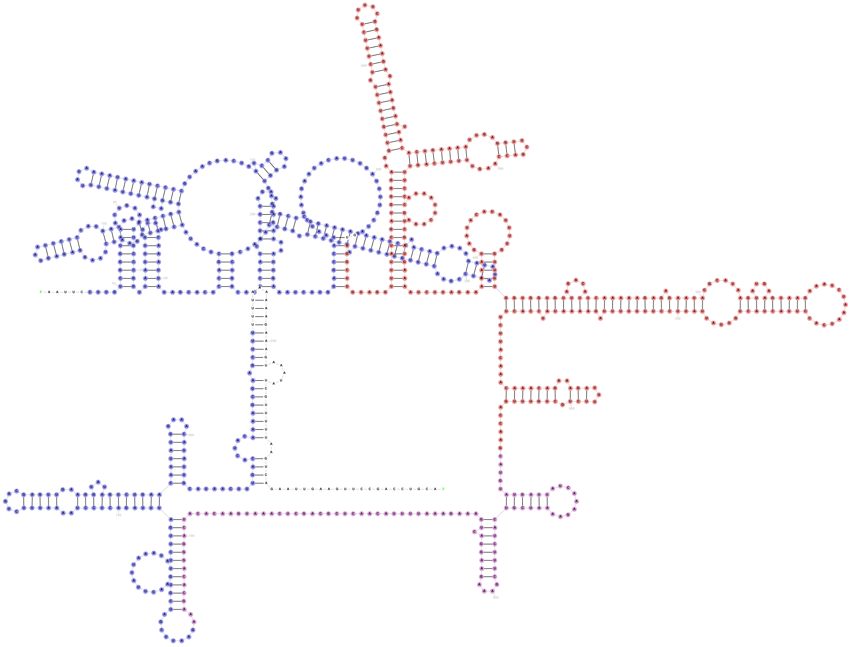

RFAM/GISSD group I CM match
embedded CDS (PHORG match)
overlap (CM and CDS)

UNK__BCP8-2.Pol

clade_8

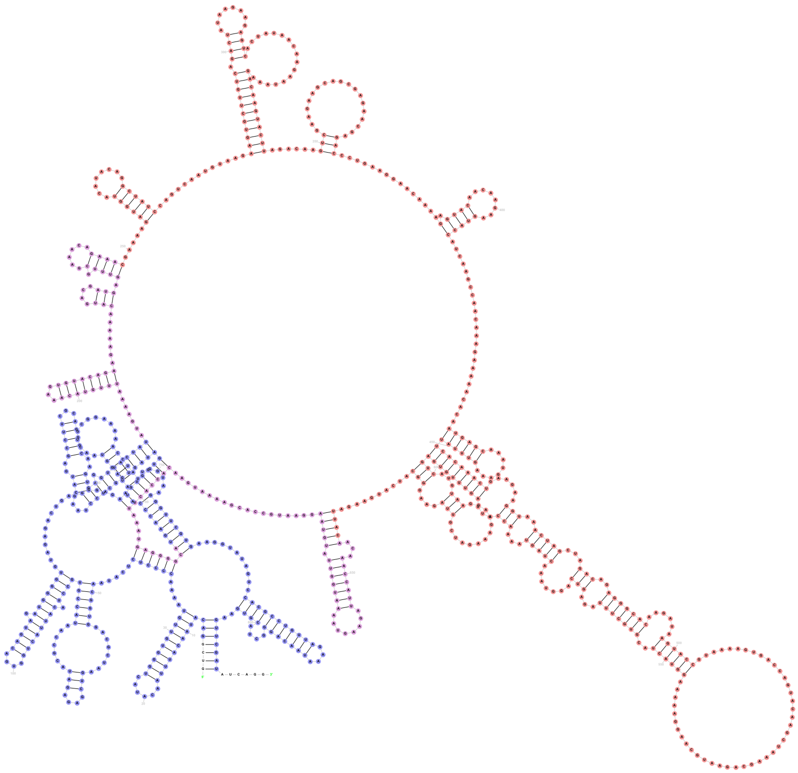

RFAM/GISSD group I CM match
embedded CDS (PHORG match)
overlap (CM and CDS)

UNK__Bobb.Pol

clade_8

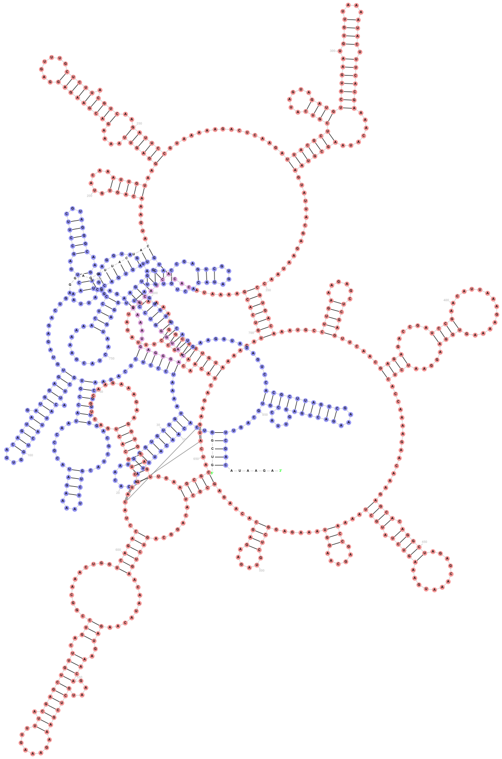

RFAM/GISSD group I CM match
embedded CDS (PHORG match)
overlap (CM and CDS)

UNK__WPh.Pol

clade_8

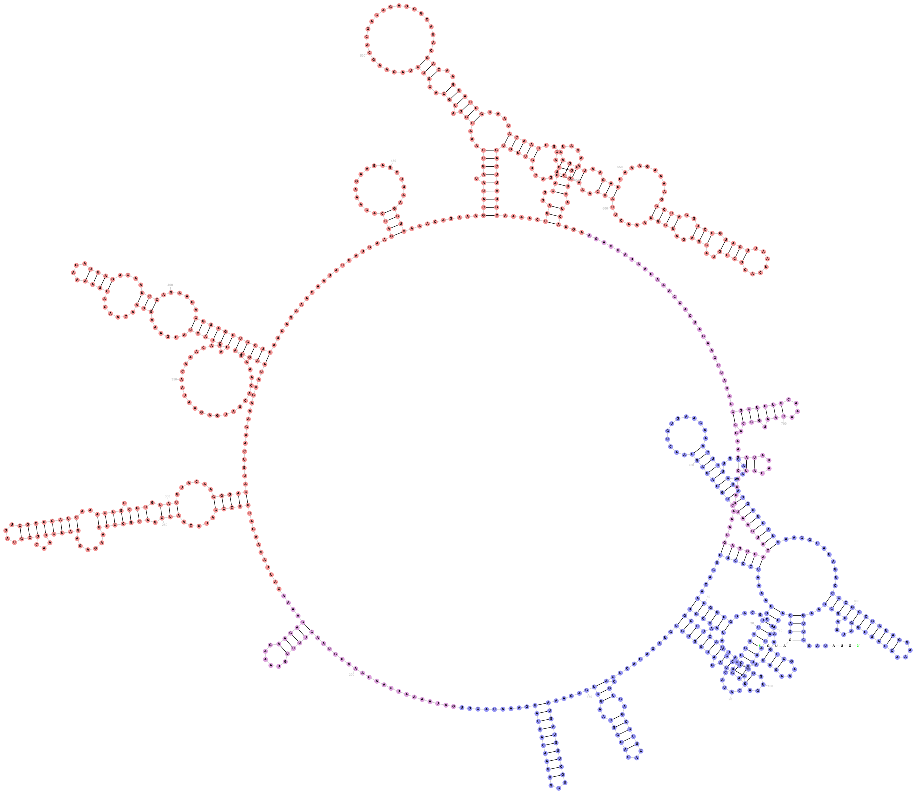

RFAM/GISSD group I CM match
embedded CDS (PHORG match)
overlap (CM and CDS)

UNK__Grass.Pol

clade_8

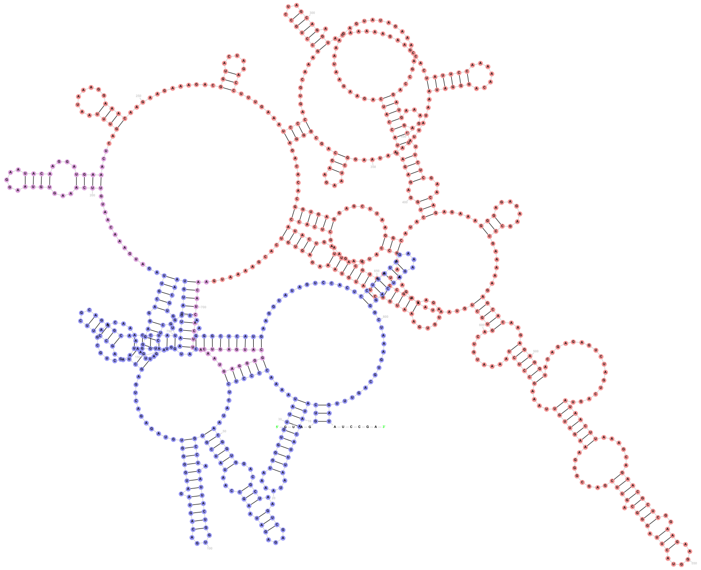

RFAM/GISSD group I CM match
embedded CDS (PHORG match)
overlap (CM and CDS)

UNK__SIOphi.Pol

clade_8

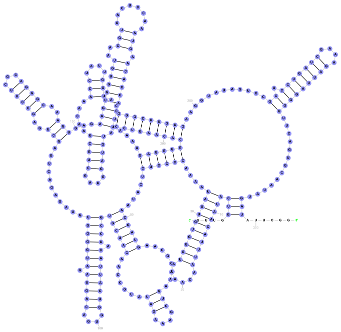

RFAM/GISSD group I CM match
embedded CDS (PHORG match)
overlap (CM and CDS)

UNK__Deep_Blue.Pol

clade_8

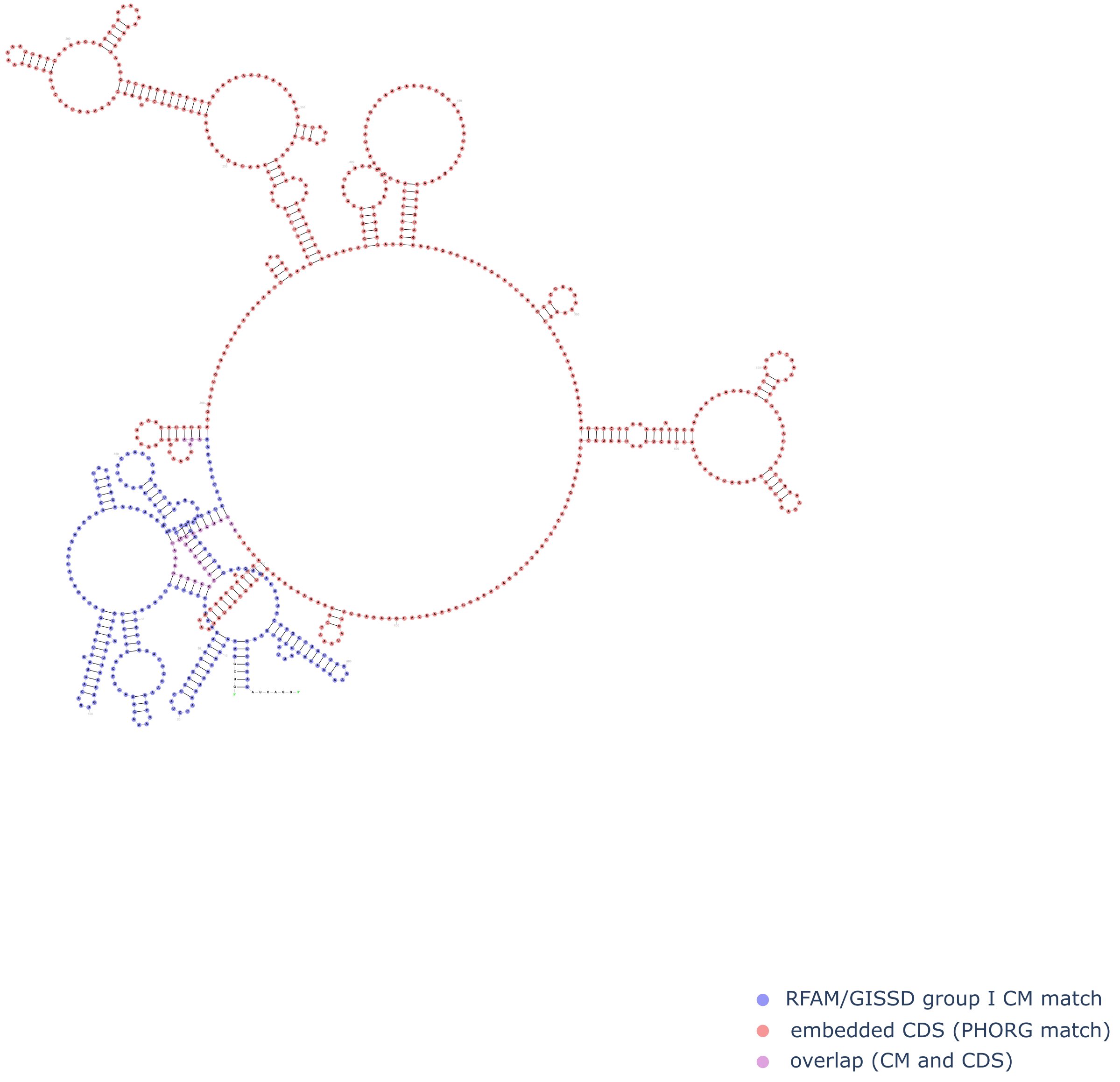

UNK__JBP901.Pol

clade_8

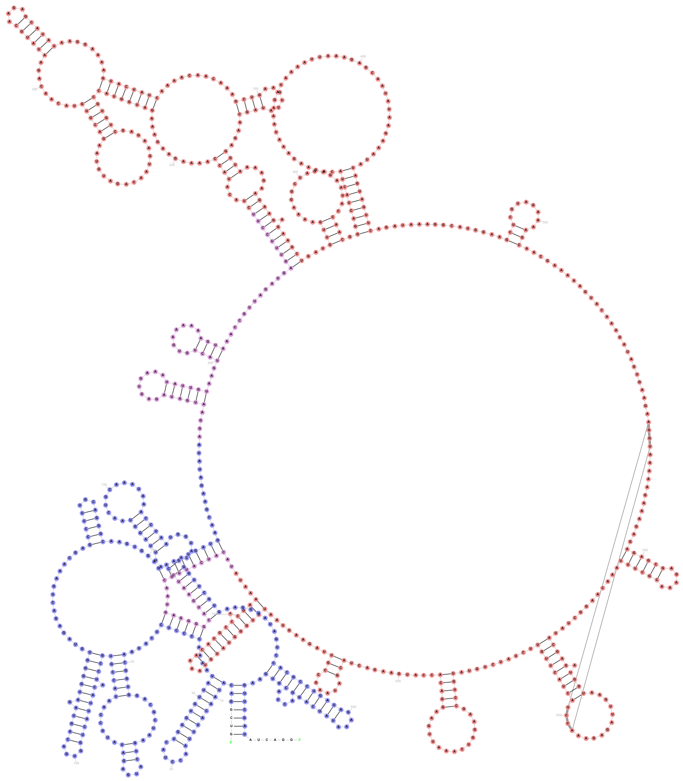

UNK__Moonbeam.Pol

clade_8

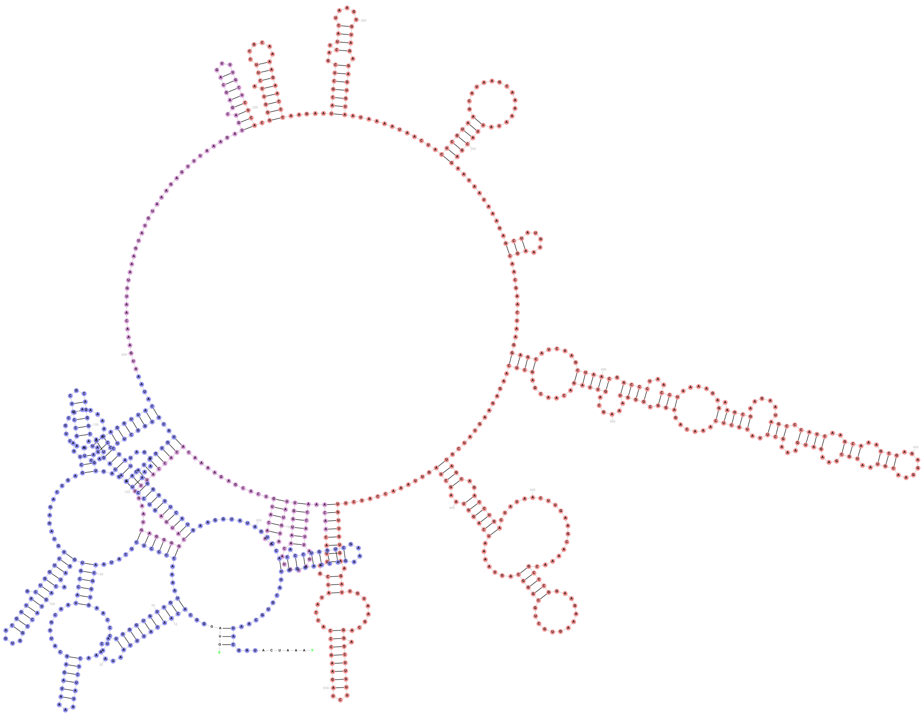

RFAM/GISSD group I CM match
embedded CDS (PHORG match)
overlap (CM and CDS)

IA1__Cpa.cL2449

clade_9

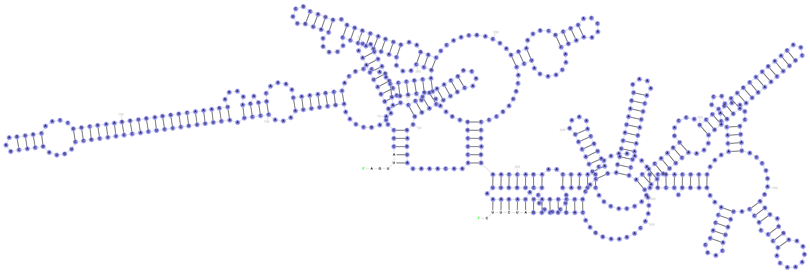

- RFAM/GISSD group I CM match
- embedded CDS (PHORG match)
- overlap (CM and CDS)

IA1__Cps.cL2449

clade_9

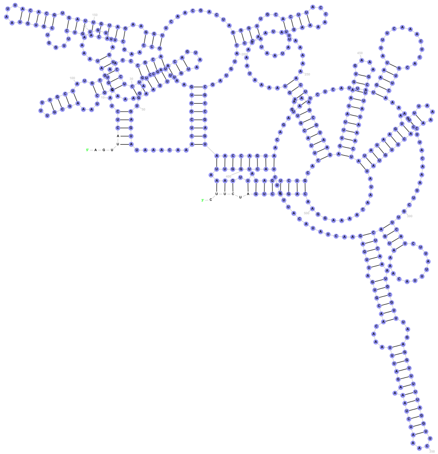

RFAM/GISSD group I CM match
embedded CDS (PHORG match)
overlap (CM and CDS)

IA1__Csp.cL2449

clade_9

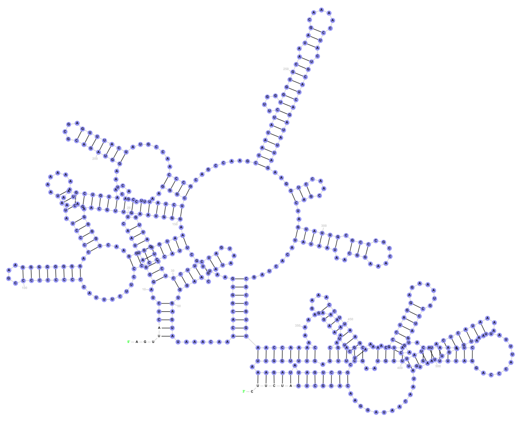

- RFAM/GISSD group I CM match
- embedded CDS (PHORG match)
- overlap (CM and CDS)

UNK__Belinda.Pol

clade_9

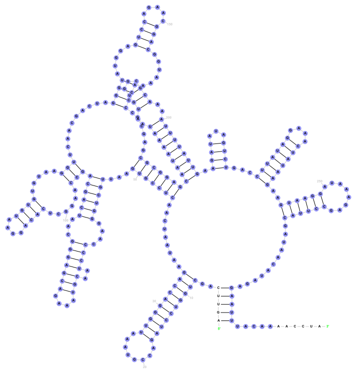

RFAM/GISSD group I CM match
embedded CDS (PHORG match)
overlap (CM and CDS)

UNK__Hakuna.Pol

clade_9

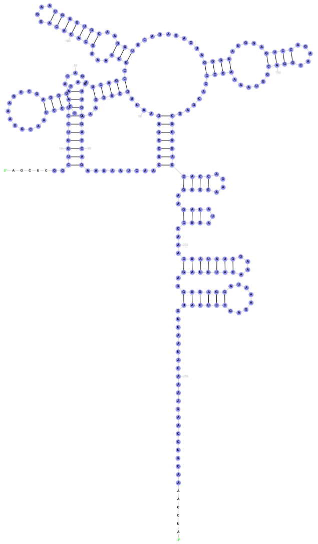

- RFAM/GISSD group I CM match
- embedded CDS (PHORG match)
- overlap (CM and CDS)

UNK__Nigalana.Pol

clade_9

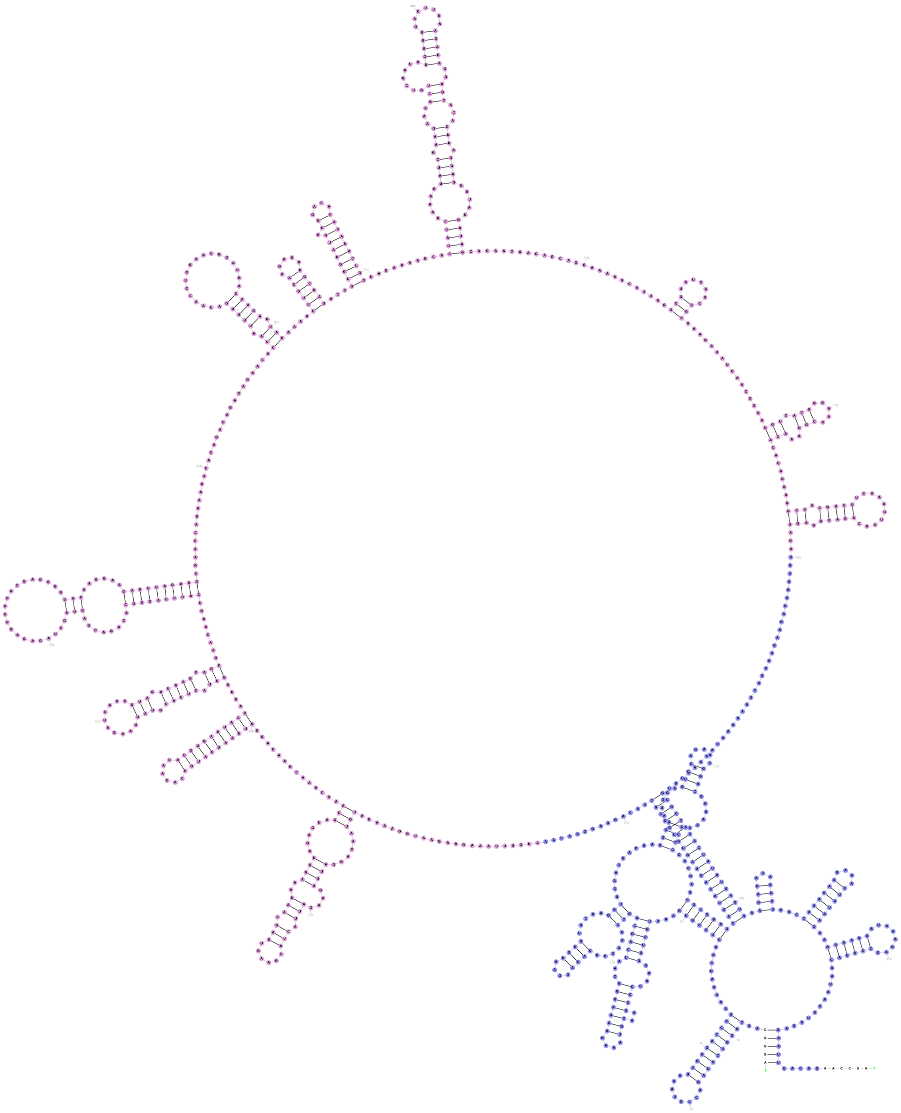

RFAM/GISSD group I CM match

embedded CDS (PHORG match)

overlap (CM and CDS)

UNK__SageFayge.Pol

clade_9

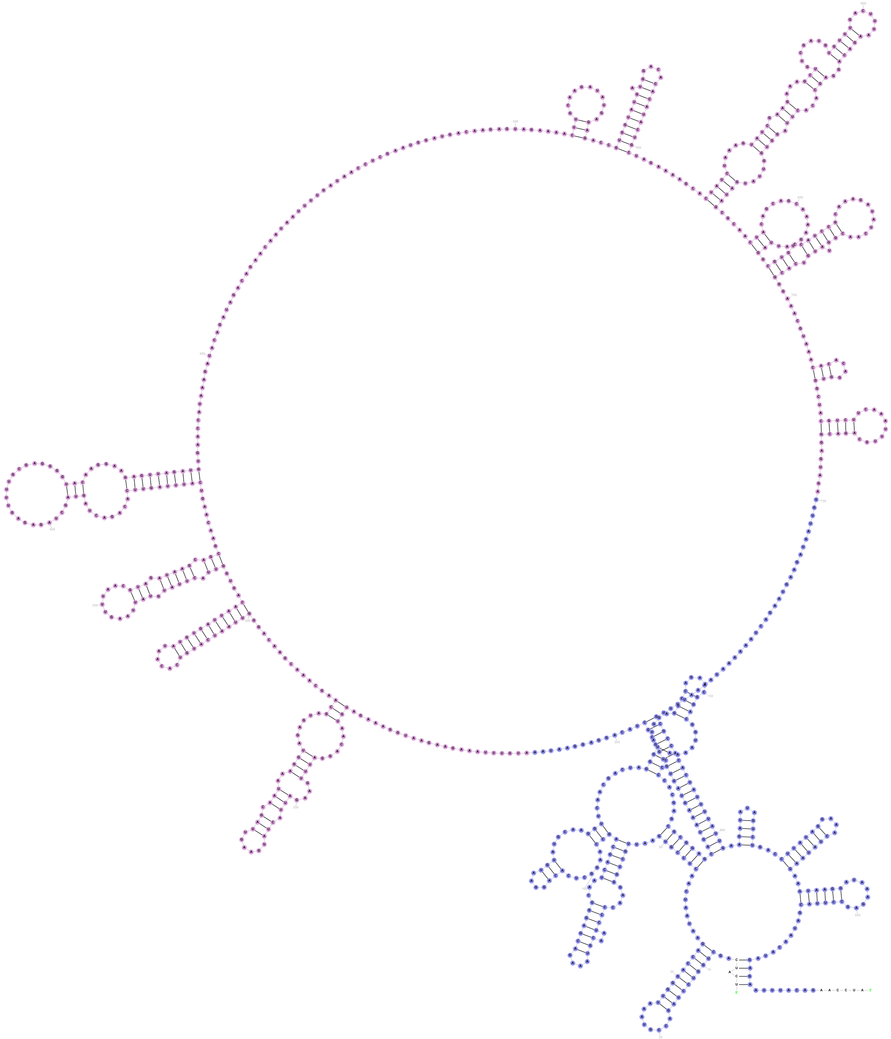

RFAM/GISSD group I CM match
embedded CDS (PHORG match)
overlap (CM and CDS)

UNK__DirtyBetty.Pol

clade_9

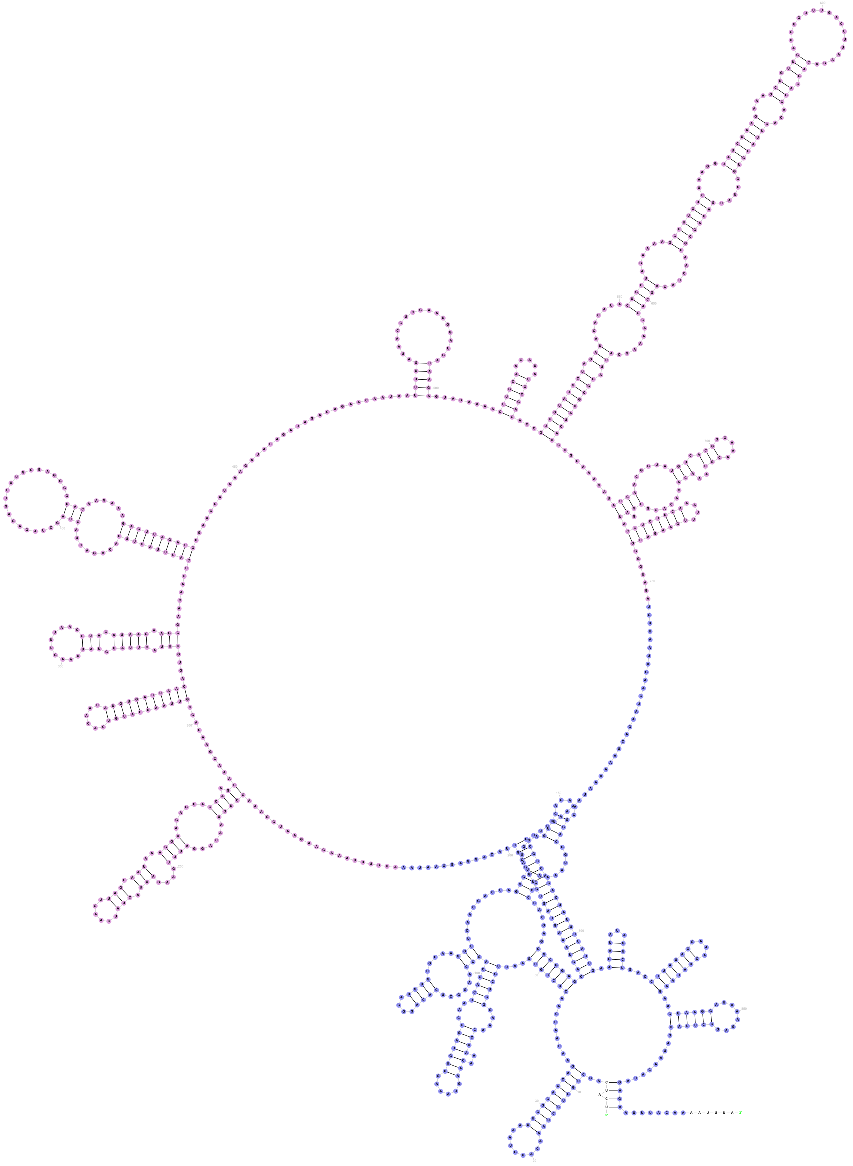

RFAM/GISSD group I CM match
embedded CDS (PHORG match)
overlap (CM and CDS)

UNK__Eyuki

clade_9

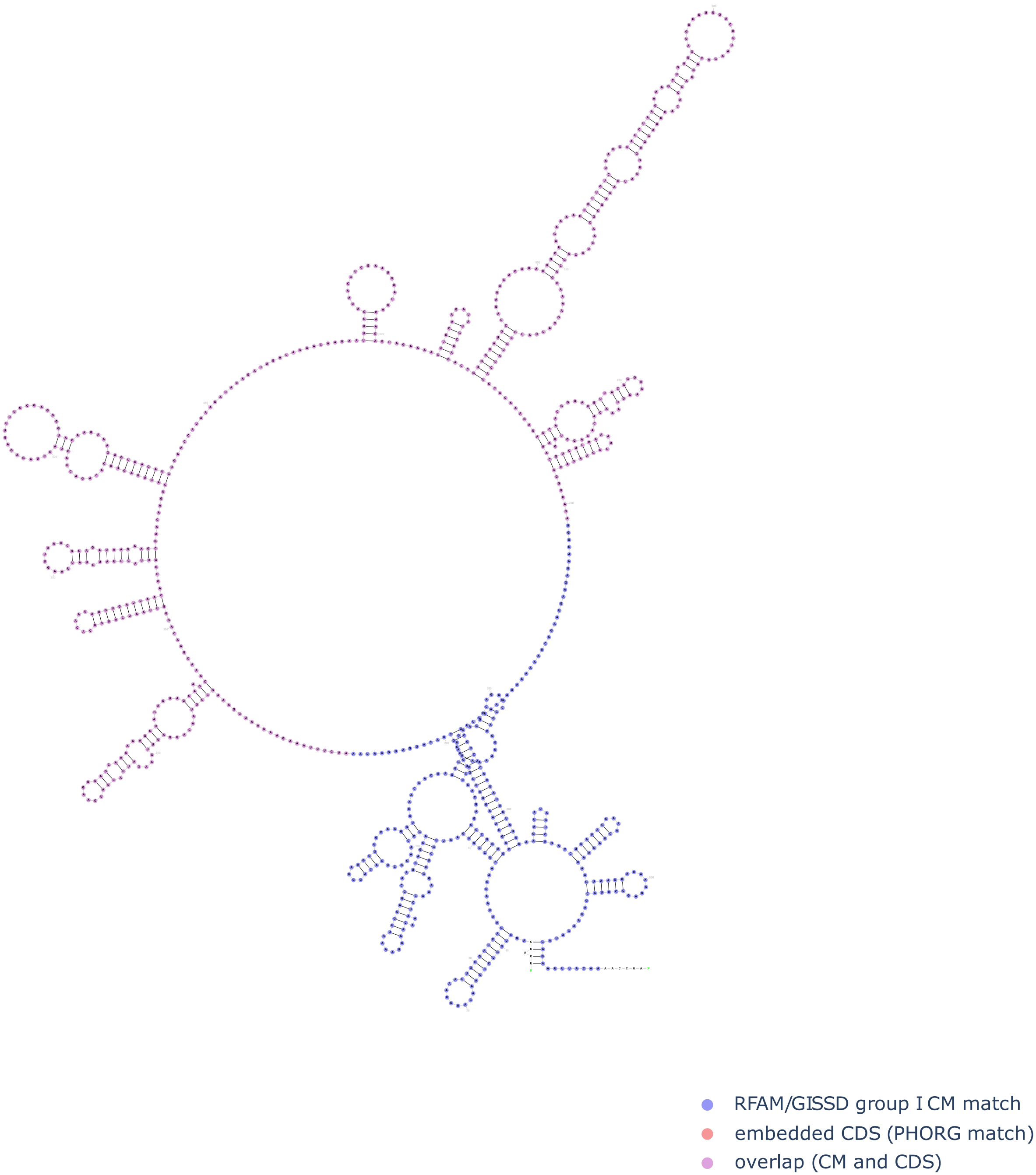

UNK__Megatron.Pol

clade_9

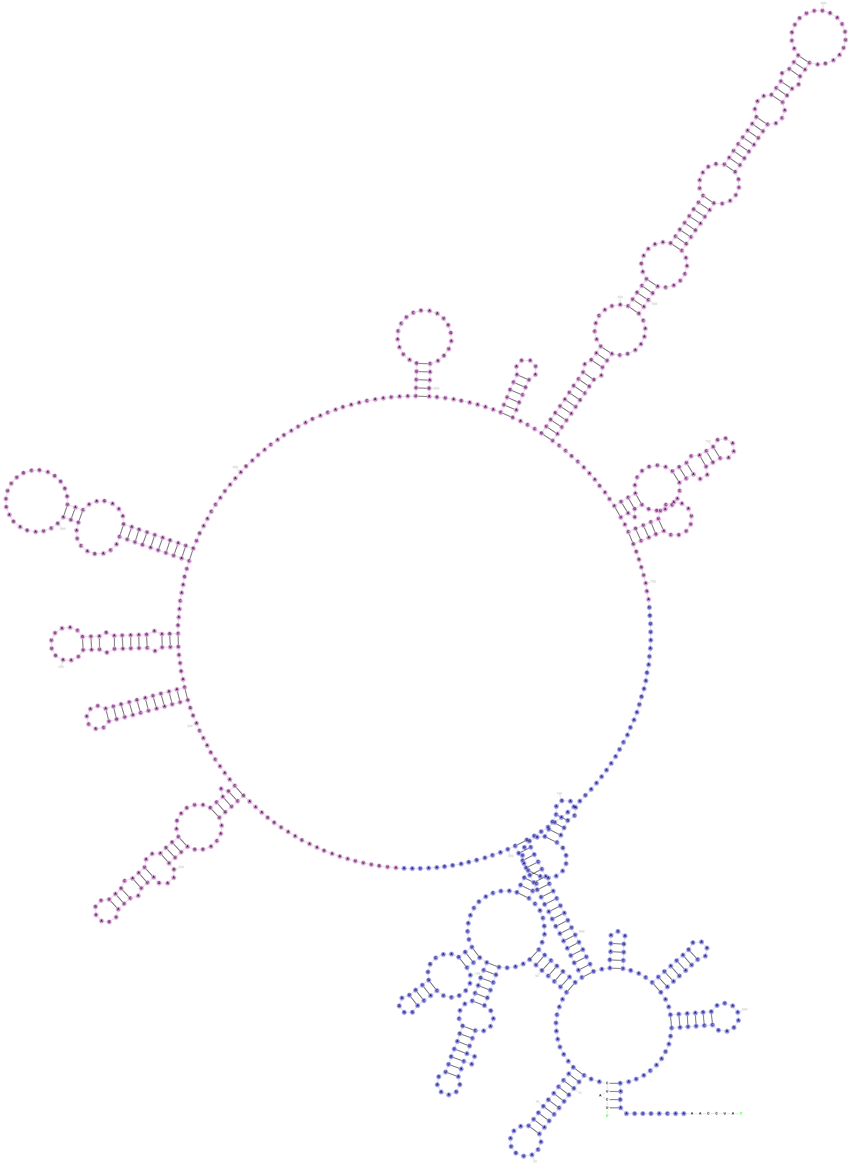

RFAM/GISSD group I CM match
embedded CDS (PHORG match)
overlap (CM and CDS)

UNK__Mater.Pol

clade_9

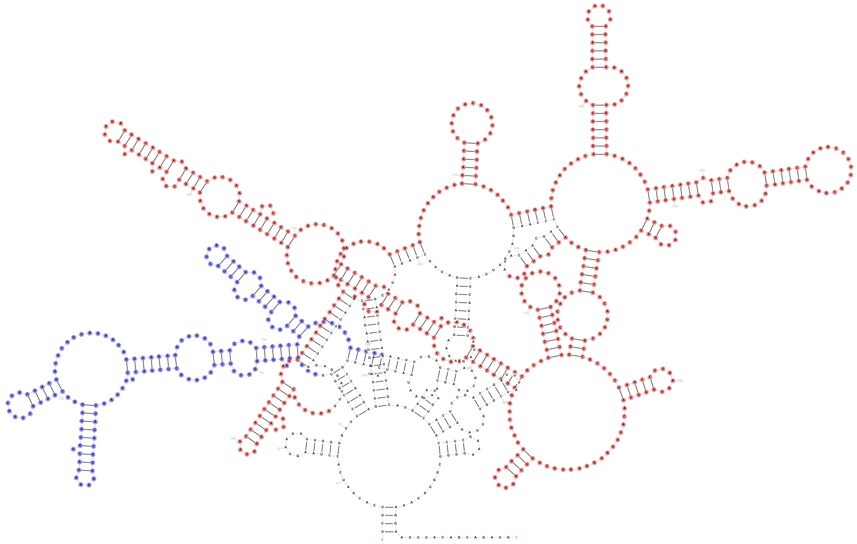

RFAM/GISSD group I CM match
embedded CDS (PHORG match)
overlap (CM and CDS)

UNK__phiNIT1.Pol

clade_9

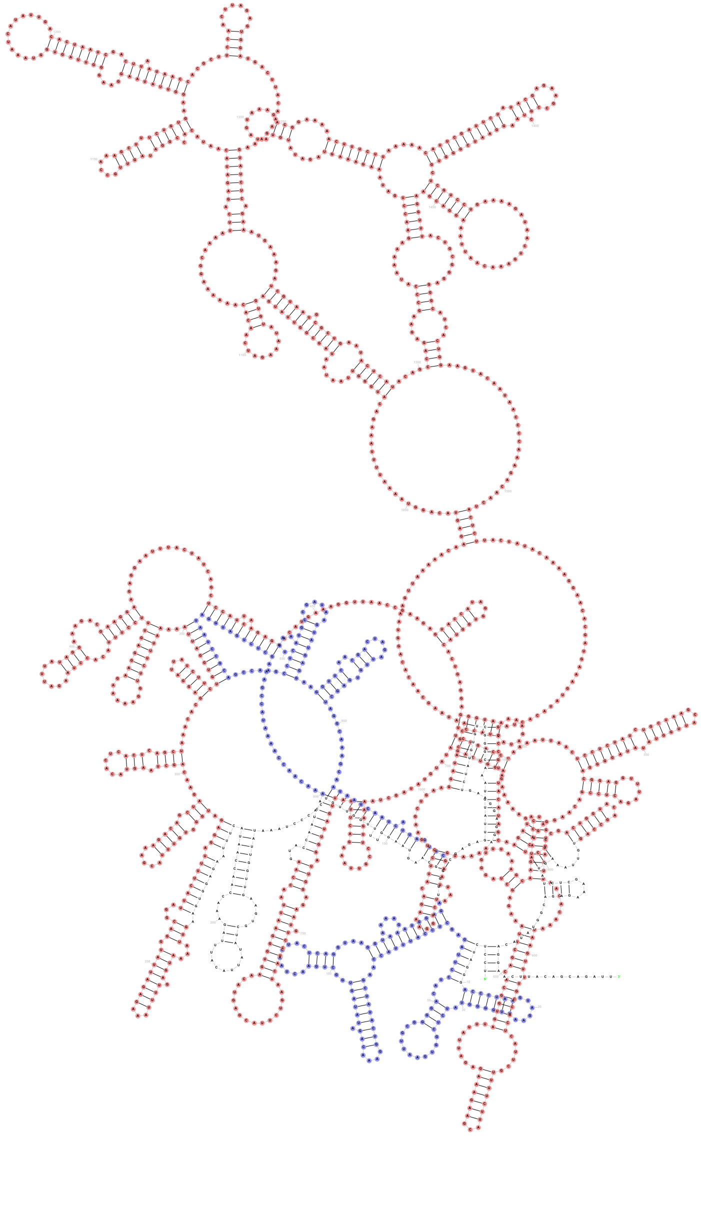

UNK__SIOphi.Pol-2

clade_9

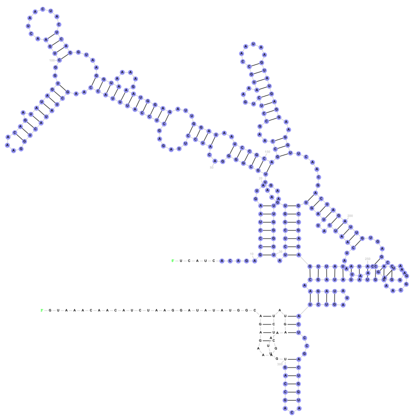

RFAM/GISSD group I CM match
embedded CDS (PHORG match)
overlap (CM and CDS)

UNK__Bastille.Pol

clade_9

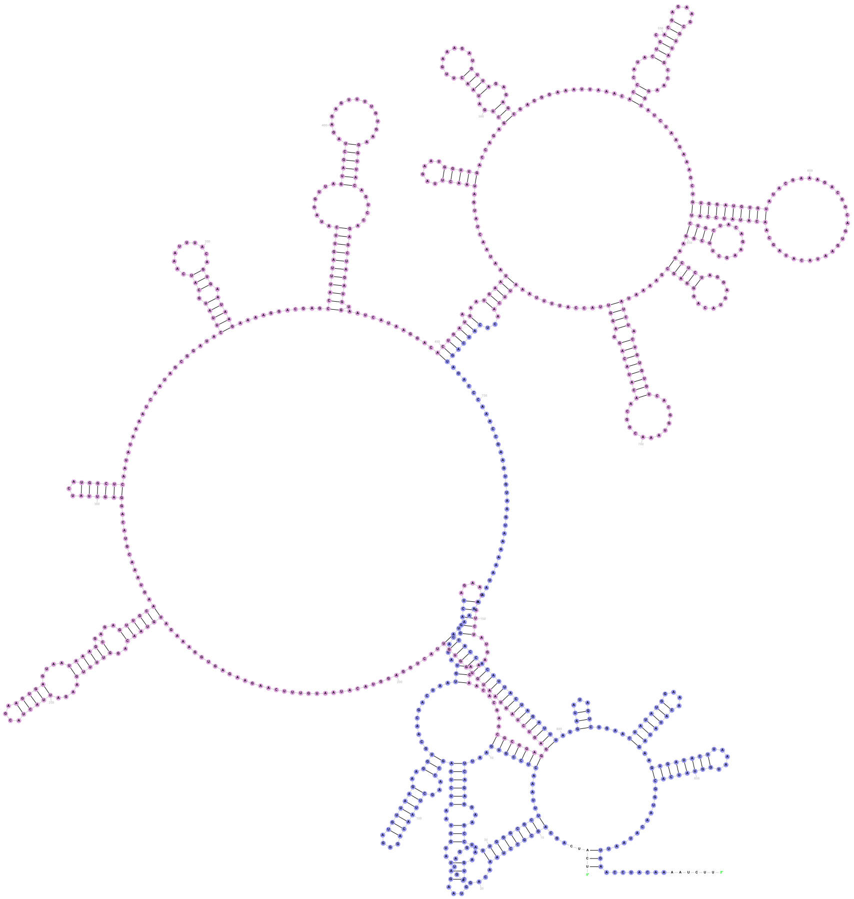

- RFAM/GISSD group I CM match
- embedded CDS (PHORG match)
- overlap (CM and CDS)

UNK__CAM003.Pol

clade_9

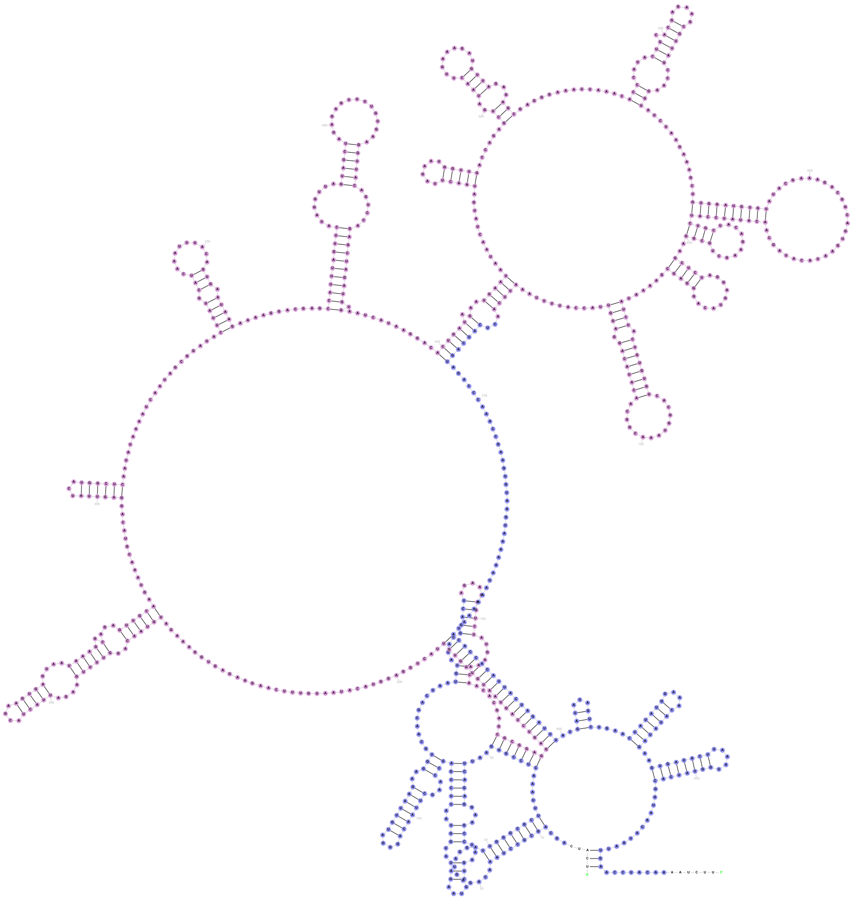

RFAM/GISSD group I CM match
embedded CDS (PHORG match)
overlap (CM and CDS)

UNK__Hoody_T.Pol

clade_9

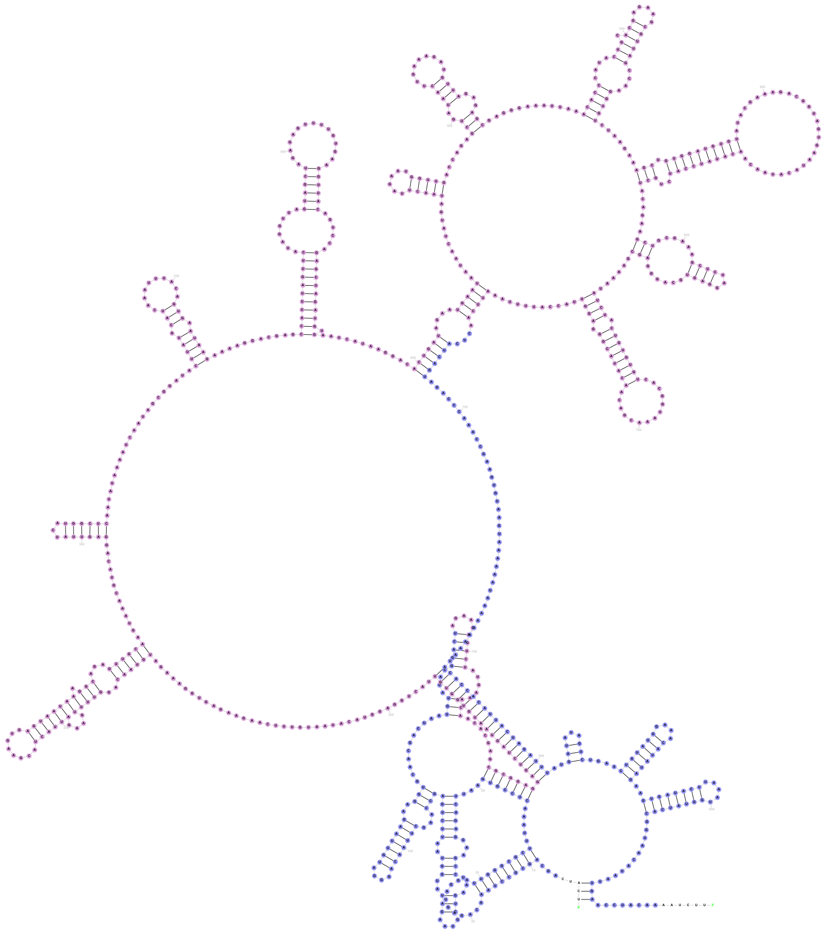

Supplement: gkaf121_Supplemental_Files [file gkaf121_supplemental_files.zip › Supplementary Figure 2.pdf]
