# Supplementary material for: Self-splicing introns in genes of Bastillevirinae bacteriophages
Source: Nucleic Acids Res. 2025 Feb 27;53(5):gkaf121. doi: 10.1093/nar/gkaf121 (PMC11878797; doi:10.1093/nar/gkaf121)

Supplementary Figure S1.

Complete maximum-likelihood tree of intron catalytic cores shown in rectangular layout.

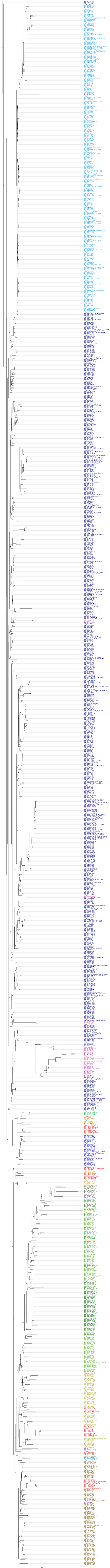

Supplement: gkaf121_Supplemental_Files [file Supplementary Figure 1.pdf › Supplementary Figure 1.pdf]
